# Histone Deacetylase Inhibition Enhances Self Renewal and Cardioprotection by Human Cord Blood-Derived CD34^+^ Cells

**DOI:** 10.1371/journal.pone.0022158

**Published:** 2011-07-18

**Authors:** Ilaria Burba, Gualtiero I. Colombo, Lidia Irene Staszewsky, Marco De Simone, Paolo Devanna, Simona Nanni, Daniele Avitabile, Fabiola Molla, Simona Cosentino, Ilaria Russo, Noeleen De Angelis, Annarita Soldo, Antonella Biondi, Elisa Gambini, Carlo Gaetano, Antonella Farsetti, Giulio Pompilio, Roberto Latini, Maurizio C. Capogrossi, Maurizio Pesce

**Affiliations:** 1 Laboratorio di Biologia Vascolare e Medicina Rigenerativa, Centro Cardiologico Monzino, IRCCS, Milan, Italy; 2 Laboratorio di Genomica Funzionale ed Immunologia, Centro Cardiologico Monzino, IRCCS, Milan, Italy; 3 Dipartimento di Scienze Cardiovascolari, Istituto di Ricerche Farmacologiche Mario Negri, Milan, Italy; 4 Istituto di Patologia Medica, Università Cattolica del Sacro Cuore, Rome, Italy; 5 Laboratorio di Aterotrombosi, Centro Cardiologico Monzino, IRCCS, Milan, Italy; 6 Laboratorio di Patologia Vascolare, Istituto Dermopatico dell' Immacolata, IDI-IRCCS, Rome, Italy; 7 Dipartimento di Oncologia Sperimentale, Istituto Regina Elena, Rome, Italy; University of Minnesota, United States of America

## Abstract

**Background:**

Use of peripheral blood- or bone marrow-derived progenitors for ischemic heart repair is a feasible option to induce *neo*-vascularization in ischemic tissues. These cells, named Endothelial Progenitors Cells (EPCs), have been extensively characterized phenotypically and functionally. The clinical efficacy of cardiac repair by EPCs cells remains, however, limited, due to cell autonomous defects as a consequence of risk factors. The devise of “enhancement” strategies has been therefore sought to improve repair ability of these cells and increase the clinical benefit.

**Principal Findings:**

Pharmacologic inhibition of histone deacetylases (HDACs) is known to enhance hematopoietic stem cells engraftment by improvement of self renewal and inhibition of differentiation in the presence of mitogenic stimuli *in vitro*. In the present study cord blood-derived CD34^+^ were pre-conditioned with the HDAC inhibitor Valproic Acid. This treatment affected stem cell growth and gene expression, and improved ischemic myocardium protection in an immunodeficient mouse model of myocardial infarction.

**Conclusions:**

Our results show that HDAC blockade leads to phenotype changes in CD34^+^ cells with enhanced self renewal and cardioprotection.

## Introduction

Progenitor cells-based ischemic tissues repair is one of the major endpoints in cardiovascular regenerative medicine. To provide substantial clinical benefits, progenitor cells should be able to engraft in sufficient numbers and differentiate into appropriate cardiovascular cell types, primarily endothelial, vascular smooth muscle cells or cardiac myocytes, or to promote ischemic tissue salvage by paracrine interaction with host tissues cells. Since 1997, the cells primarily deputed to fulfill this role are endothelial progenitor cells (EPCs) [1]. Repair efficiency of patient-derived EPCs may be limited by cell autonomous defects caused by cardiovascular risk factors that greatly reduce EPCs tolerance to stress conditions [2], their ability to produce differentiated progenies or to survive into recipient tissues [3]. Various strategies to restore innate EPC biological activity have been therefore sought [4], based on pre-treatment with drugs that restore *pro*-survival pathways, on culture in the presence of chemotactic cytokines promoting EPCs migratory activity, or on use of drugs that limit glucotoxicity or oxidative stress [5], [6].

The relevance of the so called “histone code” [7] for epigenetic control of stem cells differentiation *vs.* self renewal has been highlighted by studies showing involvement of specific histone tails modifications (acetylation, methylation) in establishment of gene expression signatures specific for pluripotent cells [8], lineage-committed cells [9] or adult-derived stem cells [10]. Given the emerging role of epigenetic phenomena as fundamental triggers of (stem) cell differentiation and plasticity, in the present study we studied whether modification of the epigenetic landscape by pharmacologic inhibition of histone deacetylases (HDAC) enzymes affects CD34^+^ cells growth, stemness, phenotype and gene expression; we also assessed whether HDACi-treated CD34^+^ cells have a modified or enhanced regeneration capacity in a mouse model of myocardial infarction.

## Results

### Effects of HDAC inhibition on CD34^+^ cells growth and proliferation

**Figure 1:**
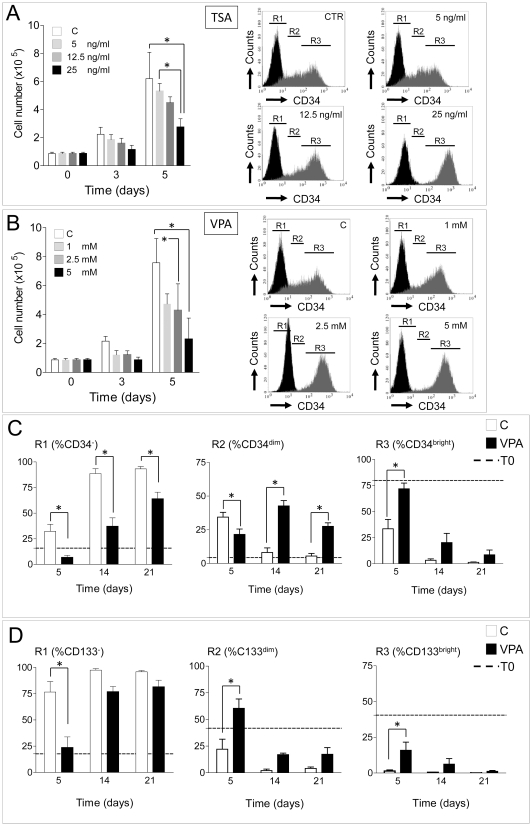
Growth inhibition and stem cell markers (CD34, CD133) expression in control and HDACi-preconditioned stem cells. (A–B) Time course experiment at 3 and 5 days showing that TSA and VPA dose-dependently inhibited cytokine-induced cellular growth and enhanced the expression of CD34 marker. R1, R2 and R3 represent the three regions corresponding to CD34^neg^, CD34^dim^ and CD34^bright^ cells, respectively, as detected by flow cytometry. (C–D) Quantification of CD34^neg^, CD34^dim^, CD34^bright^ cells and CD133^neg^, CD133^dim^ and CD133^bright^ cells at 5, 14 and 21 days of culture in the presence or the absence of 2.5 mM VPA by flow cytometry. * indicate *P*<0.05 by 2 ways ANOVA with Bonferroni post-hoc analysis (n≥4).

In the present study, a serum free culture method was used to expand CD34^+^ cells [11] in the presence of increasing amounts of Trichostatin A (TSA) and Valproic Acid (VPA), two wide range HDAC inhibitors. As shown in Figures S1 and S2, morphology of HDACi-treated cells was dose-dependently affected by treatment with both drugs. To identify whether specific subpopulations were affected by treatment with increasing VPA and TSA concentrations, a flow cytometry analysis was performed by grouping cells into three discrete regions: R1, corresponding to CD34^neg^ cells, R2, corresponding to CD34^dim^ and R3, corresponding to CD34^bright^ cells; in parallel, cellular growth was assessed by counting at two consecutive time points. Figure 1A and B show results of a 5 day time course analysis; increasing doses of TSA and VPA decreased cellular growth with a dose-dependent shift toward a homogeneous CD34^bright^ cell population. To assess the time extension of HDAC inhibition on CD34 and CD133 expression, a flow cytometry analysis was performed in cells cultured in the presence of 2.5 mM VPA at 5, 14 and 21 days (Figure 1C, D; Figure S3).

**Figure 2:**
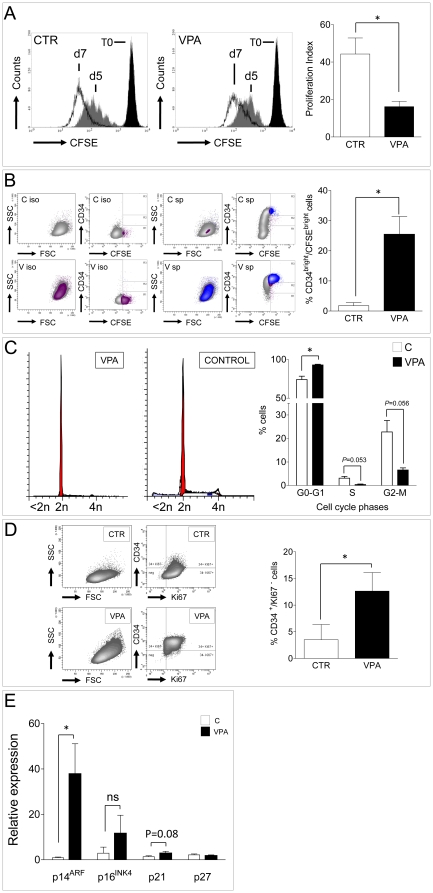
Analysis of CD34^+^ cells proliferation in the presence and the absence of VPA by flow cytometry. (A) CFSE staining profiles of control and VPA-treated cells at 5 and 7 days of culture. Note that the fluorescence intensity reduction as a consequence in cell proliferation was less pronounced in VPA *vs.* CTR cells at both time points. Proliferation index at 7 days was significantly reduced. (B) Seven days VPA treated cells had a higher frequency of slow dividing immature (CD34^bright^) stem cells (blue area in contour plots), as detected by co-staining with CFSE and CD34 antibody; plots on the left indicate the fluorescence profile of cells stained with CFSE and CD34 isotype antibody (iso). (C) Cell cycle analysis in 7 days CTR and VPA-treated cells revealed a higher frequency of cells in the G0–G1 and a lower percentage in either S and G2-M phases. (D) The percentage of cells specifically arrested in G0 was also increased, as detected by co-staining with anti Ki67 and CD34 antibodies at the same time point. * indicate *P*<0.05 by paired t-test (n≥3). (E) Quantification of the relative expression level (2^−ΔΔCt^ method) of small cyclin/CDK inhibitors (p14^ARF^, p16^INK4^, p21^Cip1/waf1^ and p27) by qRT-PCR analysis. * indicates *P*<0.05 by unpaired t-test (n≥3).

TSA was not used in subsequent experiments for the reason that this molecule is not routinely clinically used, such as VPA, for treatment of diseases [12], [13]. On the other hand, dose-response treatments indicated that 2.5 mM VPA had a marked effect on CD34 expression enhancement without producing maximal growth retardation, but it did not have a significant effect on cell death (trypan blue exclusion test in cells cultured for 7 days: CTR *vs.* VPA cells: 13.5%±2.7% *vs.* 15.7%±1.77%; *P* = 0.675 paired t-test) or apoptosis (percent of cells in sub-G1 phase as detected by flow cytometry in PI-stained cells cultured for 7 days: 4.9±3.2 *vs.* 1.2±0.4; CTR *vs.* VPA; mean ± SE, n = 3, *P* = 0.38, paired t-test). VPA 2.5 mM dose was then used in all the following experiments. To clarify the relationship between stem cells generation time and retardation in cell cycle progression, Carboxyfluorescein Succinimidyl ester (CFSE) dye was used to assess CTR and VPA growth progression at 5 and 7 days and to calculate proliferation index and cellular generations number in the presence and absence of VPA [14]. Figure 2A and B show that, at 7 days, proliferation index was significantly reduced in VPA *vs.* CTR cells and that CD34^bright^/CFSE^bright^ cells after VPA treatment were increased. This corresponded to an average 1–2 generations delay in the presence of VPA (Figure S4A) and to a specific growth retardation of CD34^bright^ cells (Figure S4B). Cell cycle analysis and double staining with antibodies recognizing CD34 and Ki67 confirmed that 7 days VPA treatment caused a significant elongation of G1 phase and a slight, but significant, increase in G0 cells (CD34^+^/Ki67^−^; Figure 2 C and D). Finally, as shown in Figure 2E, expression of CDK inhibitors (p14^ARF^, p16^INK4^, p21^Cip1/Waf-1^) was increased at the same time point, although at a different extent, in VPA *vs.* CTR cells.

### Histone hyperacetylation is directly linked to enhanced CD34^+^ expression and increased self renewal

**Figure 3:**
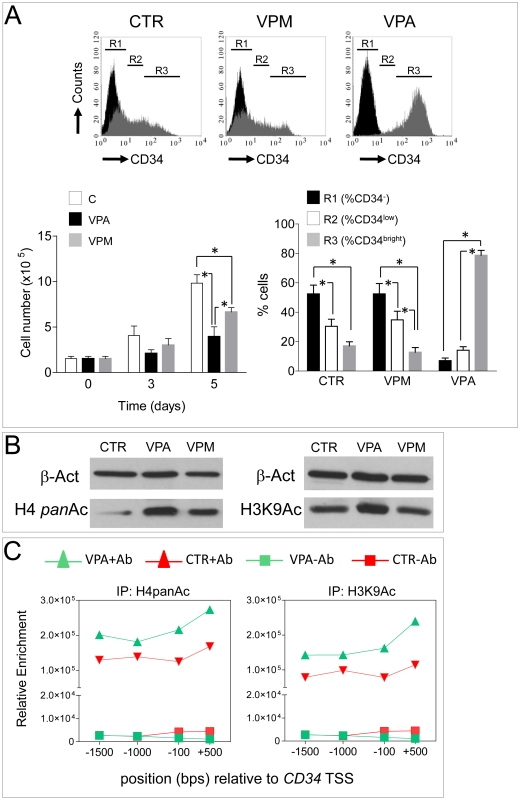
Effect of VPA is directly related to HDAC inhibition. (A) The VPA structural analogue Valpromide (VPM) reduced CD34^+^ cells proliferation at lower extent compared to continuous treatment with VPA in a 5 day time course experiment. By contrast, the CD34 expression profile was identical to that of control cells, as detected by flow cytometry. (B) Western blotting showing that a 7 day VPA treatment induced hyper acethylation on H4 and H3 (at lysine residue 9) histones. * indicate *P*<0.05 by two ways ANOVA with Bonferroni post hoc analysis (n≥3). (C) Representative ChIP experiment showing an increased enrichment of various sites in the *CD34* gene promoter (upstream and downstream of the TSS), as a result of chromatin hyper-acetylation (*pan*-H4Ac, H3K9Ac) due to VPA treatment. Data are expressed as relative enrichment calculated by real time PCR amplification.

To determine whether shifting toward a homogeneous CD34^bright^ phenotype is consequent to histone hyperacetylation resulting from HDAC inhibition, Valpromide (VPM), a non-teratogenic VPA derivative with lower HDAC inhibition activity [15], was used in parallel to VPA in a 5 day time course experiment. VPA substitution with VPM inhibited at intermediate levels cellular growth, while it produced a CD34 expression profile in R1, R2 and R3 regions similar to CTR cells (Figure 3A). Western blotting using *pan*-acetylated histone H4 (H4Ac) and lysine 9 acetylated histone H3 (H3K9Ac) showed that 7 days VPA treatment specifically enhanced histone tails acetylation (Figure 3B). An immunoprecipitation (ChIP) experiment was then performed with chromatin extracted by cells grown for 7 days in the presence or the absence of VPA, using anti-H4Ac and anti- H3K9Ac antibodies, followed by real time PCR to detect enrichment of specific *CD34* promoter regions. As shown in Figure 3C, H4Ac and H3K9Ac association to various regions upstream and downstream of the *CD34* promoter transcriptional start site (TSS) was increased by VPA treatment, suggesting epigenetic regulation of *CD34* expression.

### Phenotype analysis, clonogenic activity and gene profiling of HDACi treated CD34^+^ cells

**Figure 4:**
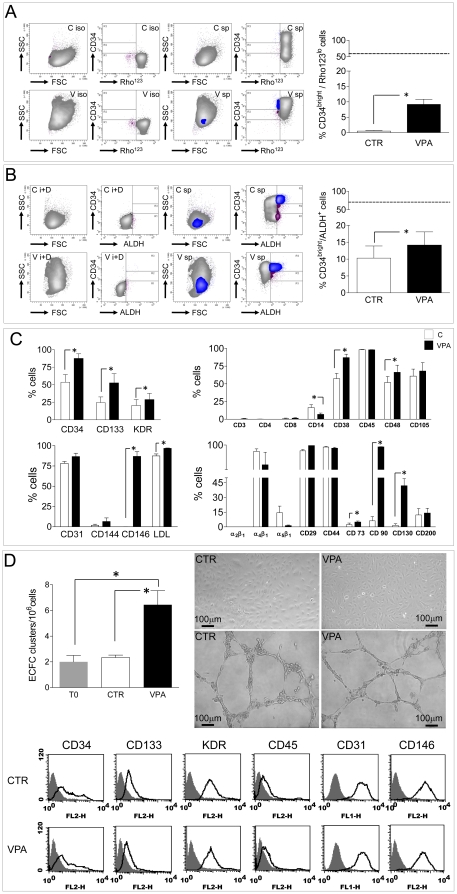
Phenotype and stem cell function in control and 7-days VPA treated cells by flow cytometry. (A) quantification of cells actively extruding Rhodamine123 dye as a result of ABCG2 gene product MDR-1, a typical activity of immature stem cells. Contour plots on the left show the staining profile in the presence of the MDR-1 pump inhibitor Verapamil, plus the CD34 isotype (ISO), while those on the right show the shift toward the left of a CD34^bright^ cells fraction (blue area) in VPA-treated cells (V). Bar graph on the right indicates quantification of CD34^bright^/Rho123^lo^ cells in C and VPA conditions; dotted line indicate the percentage of CD34^bright^/Rho123^lo^ cells immediately after isolating CD34^+^ cells from cord blood. (B) Quantification of ALDH expressing cells. Contour plots on the left show the fluorescence profile of cells treated with the ALDH inhibitor DEAB (used as a negative control) and CD34 isotype antibody (i+D), while those on the right show the results of specific staining with CD34 antibody and fluorescent detection of ALDH activity. Note that in the presence of VPA a higher percentage of CD34^bright^/ALDH^+^ cells was present (blue area), while in control cells ALDH staining was lower in the CD34^bright^ gating and present in CD34^dim/neg^ cells (areas in magenta color). Bar graph on the right indicates quantification of CD34^bright^/ALDH^+^ cells in C and VPA conditions; dotted line indicate the percentage of these cells immediately after isolating CD34^+^ cells from cord blood. * indicate P<0.05 by paired t-test (n = 4). (C) phenotype analysis of control and VPA cells at 7 days of culture by multiparametric flow cytometry experiments. Upregulation of stem cells markers CD34, CD133, CD38 and KDR were found along with enhanced expression of mesenchymal markers CD90, CD146 and CD130. Consistent with an effect of VPA on myeloid differentiation inhibition, CD14 was inhibited. * indicate *P*<0.05 by paired t-test (n≥3). (D) Derivation of ECFCs from fresh, CTR and VPA CD34^+^ cells. Formation of clusters was observed three weeks after plating these cells onto FN coated dishes. Histogram represents number of ECFC clusters observed in three independent experiments; * indicates *P*<0.05 by one way ANOVA with Neuman Keuls post-hoc. Pictures on the upper right show the morphology of ECFCs derived from CTR and VPA CD34^+^ cells and their ability to form capillary-like structures, when plated onto matrigel. The amount of these latter structures formed by either CTR or VPA cells was not different compared with HUVEC cells that were used as a positive control (not shown). Plots in the lower part of the panel show expression of typical ECFC markers [19].

Figure S5 shows the experimental design adopted for phenotypic and molecular characterization of HDACi-treated CD34^+^ cells. Seven days VPA-treated cells were first analyzed to measure the ability to extrude Rhodamine123 (Rho123) drug, a typical primitive stem cell feature relative to the *MDR-1* gene product expression [16], and verify expression of Aldehyde Dehydrogenase (ALDH) [17] (Figures 4A, B and S6). These tests showed that VPA maintained a higher percentage of CD34^bright^/Rho123^lo^ and CD34^bright^/ALDH^+^ cells compared with controls. Antibodies were then used to detect expression of stem cell (CD34, CD133, KDR) hematopoietic (CD3, CD4, CD8, CD14, CD38, CD45, CD48), endothelial (CD31, CD105, CD144, CD146, LDL uptake), mesenchymal (CD73, CD90, CD130, CD200) and integrin (α_2_β_1_, α_4_β_1_, α_5_β1) markers in multiparametric flow cytometry in CTR and VPA cells at 7 days. Results (Figure 4C) indicated that VPA significantly enhanced CD34, CD38, CD48, CD133 and KDR expression, but also caused *de novo* expression of CD90, CD130 and CD146. Data obtained by prolonging the culture time up to 14 days (Figure S7) confirmed increased CD34, CD133 and KDR expression, induction of CD146 and increased CD31, and maintenance of a higher LDL uptake ability. Interestingly, expression of CD14 was significantly inhibited at both time points.

Recently, long term proliferating EPCs from cord blood cells characterized by CD34, CD38, CD45 and CD133 markers have been obtained using clonal culture conditions [18]. These cells resemble those named Endothelial Colony Forming Cells (ECFCs) in previous definitions of human EPCs identity [19]. To assess whether VPA-treated CD34^+^ cells have a higher ability to form ECFC clusters, these cells were plated under ECFC inducing conditions [20]. Results showed that derivation of ECFC clusters was higher from VPA cells compared with CTR and freshly isolated CD34^+^ cells. Despite this, morphologic appearance, expression of ECFC markers and the ability to form capillary-like structures onto Matrigel was not different in CTR *vs.* VPA cells-derived ECFCs (Figure 4D).

**Figure 5:**
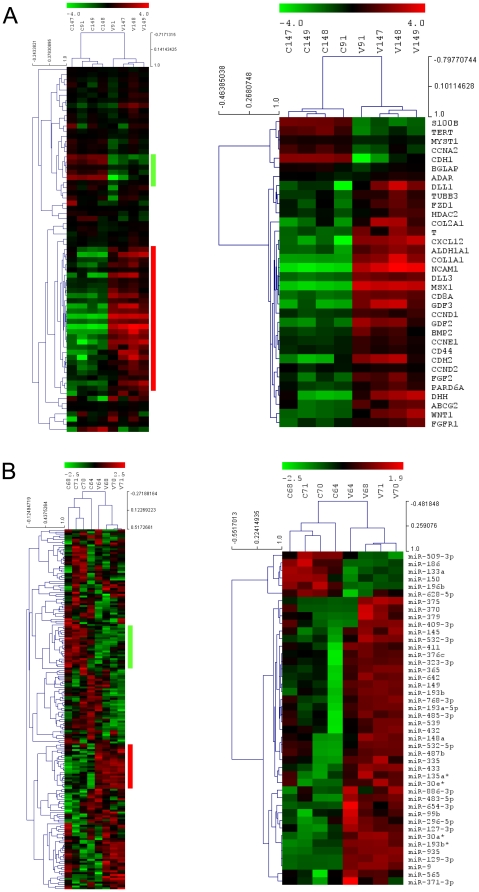
Unsupervised hierarchical cluster analysis and statistical analysis of mRNA and miRNA profiling in CTR *vs.* 7 days VPA treated CD34^+^ cells (A and B, respectively). The analyses were performed initially using the whole datasets of genes (panel A, left heat map) or miRNAs (panel B, left heat map) that passed the quality assurance and filtering criteria (see Supplementary Methods), to assess whether expression profiles discriminates treatment groups. A second round of unsupervised hierarchical clustering was done on differentially expressed genes (panel B, right heat map) or miRNAs (panel C, right heat map), as selected by significance analysis (see Supplementary Methods), to identify biologically relevant co-expressed gene clusters. The mean centered level of expression of each gene/miRNA in each sample is represented with green, black, and red colour scales (green indicates below mean; black, equal to mean; and red, above mean). The dendrograms on top of each heat map display the unsupervised clustering of control and VPA-treated CD34^+^ cells using the whole or the differentially expressed gene/miRNA lists. The dendrograms on the left side of each heat map show the unsupervised clustering of the genes. Correlation coefficients are reported for both. See Supplementary Methods for data transformation and adjustments, distance metrics and linkage methods.

To investigate possible gene expression changes in 7-days cultured VPA *vs.* CTR cells, a survey of stem cells-specific transcripts expression was performed using qRT-PCR low density arrays (Figure 5A). Unsupervised clustering analysis recognized coherently upregulated or downregulated stem cell-associated genes, allowing discriminate between VPA *vs.* CTR cells signatures. Statistical analysis revealed 34 transcripts significantly modified by VPA. Of these, 6 were down-modulated while 18 were up-regulated. Finally, 10 genes that were not detectable or were at detection limit by qRT-PCR, were expressed *de novo* by HDAC inhibition (Table 1). A microRNA (miRNA) profiling was finally performed on high-throughput sorted CTR and VPA CD34^bright^ cellular populations (Figure S8). Remarkably, unsupervised clustering clearly distinguished miRNA profiles of CD34^bright^ cells in VPA *vs.* CTR conditions (Figure 5B). Results from statistical analysis identified 44 miRNAs whose expression was significantly modified. Of these, 6 were downregulated, 31 were upregulated, while 7 were expressed *de novo* (Table 2).

**Table 1:** Identity of stem cell genes coherently modulated by 7-days VPA treatment in CD34^+^ cells.

Gene	Main function	*P* value	Fold Change V *vs.* C	FDR
S100B	Muscle/Neuronal Differentiation	0,000157	−9,62	0,002788
CDH1	E-Cadherin, cell adhesion	0,039466	−8,68	0,084911
TERT	Stemness	0,000418	−5,82	0,004244
CCNA2	Cell cycle	0,008564	−2,97	0,028954
MYST1	HAT	0,007580	−1,70	0,029901
BGLAP	MSCs bone differentiation	0,023935	−1,34	0,054819
ADAR	A→I RNA editing	0,042739	1,33	0,089249
CCND2	Cell cycle	0,008373	1,47	0,029725
CD44	MSCs marker	0,004531	2,06	0,018922
CCNE1	Cell cycle	0,002891	2,45	0,015789
HDAC2	Histone deacethylase	0,018523	2,53	0,045350
PARD6A	Cell polarity	0,016895	3,01	0,042842
TUBB3	Cell polarity	0,002173	3,19	0,014024
FZD1	Wnt pathway	0,002543	3,20	0,015048
CCND1	Cell Cycle	0,001493	3,31	0,011784
FGFR1	Angiogenesis	0,010671	3,73	0,031569
FGF2	Angiogenesis	0,004297	4,86	0,019068
COL2A1	Extracellular Matrix	0,014215	9,98	0,038819
ALDH1A1	Stemness	0,000098	10,01	0,002320
DLL1	Notch pathway	0,003143	10,71	0,015942
DHH	Mesenchyme differentiation	0,019533	12,44	0,046228
CDH2	N-cadherin, neurogenesis	0,011550	13,61	0,032801
CXCL12	Angiogenesis	0,003988	25,48	0,018877
COL1A1	Extra cellular matrix	0,000792	32,27	0,007033

Gene	Main function	*P* value	Fold Induction V *vs.* C	FDR
BMP2	Mesenchyme differentiation	0,008608	3,68	0,027781
ABCG2	Stemness	0,051252	5,34	0,106239
T (Brachyury)	Mesoderm programming	0,007826	7,56	0,029243
CD8A	Hematopoietic marker	0,000414	9,61	0,004902
DLL3	Notch pathway	0,000071	9,91	0,002516
WNT1	Wnt pathway	0,015975	9,97	0,042008
GDF2	Mesenchyme differentiation	0,001962	12,24	0,013927
GDF3	Stem cells self renewal	0,010303	25,43	0,031806
MSX1	Mesenchyme differentiation	0,000001	79,96	0,000078
NCAM1	Neurogenesis	0,000253	142,31	0,003599

*P* values are calculated by a multivariate paired *t*-test, using 100 permutations and limiting the false discovery rate (FDR) proportion to <0.1 (see Supplementary Methods). C: CD34^+^ control cells; V: VPA-treated CD34^+^ cells.

**Table 2:** Identity of miRNAs modulated by 7-days VPA treatment in CD34^bright^ cells.

miRNA	*P* value	Fold change V *vs.* C	FDR
miR-196b	0,000833	−9,79	0,0288
miR-133a	0,006061	−3,65	0,0589
miR-509-3p	0,013360	−3,49	0,105
miR-150	0,022776	−2,74	0,122
miR-186	0,027408	−2,64	0,139
miR-628-5p	0,020783	−2,57	0,121
miR-145	0,046001	2,13	0,181
miR-99b	0,045319	2,25	0,181
miR-135a*	0,031687	2,34	0,144
miR-376c	0,038989	2,57	0,165
miR-487b	0,031111	2,66	0,144
miR-30e*	0,036257	2,97	0,161
miR-565	0,018084	3,24	0,119
miR-539	0,022368	3,41	0,122
miR-433	0,009603	3,46	0,0791
miR-296-5p	0,006839	3,46	0,0592
miR-432	0,018695	3,64	0,119
miR-532-5p	0,016699	3,80	0,116
miR-532-3p	0,004757	3,90	0,0514
miR-411	0,028407	4,07	0,139
miR-485-3p	0,016160	4,11	0,116
miR-371-3p	0,038970	4,26	0,165
miR-768-3p	0,006841	4,30	0,0592
miR-886-3p	0,021024	4,59	0,121
miR-409-3p	0,040590	4,80	0,167
miR-323-3p	0,019185	4,86	0,119
miR-193a-5p	0,006131	5,36	0,0589
miR-148a	0,003356	5,42	0,0447
miR-335	0,004402	5,43	0,0508
miR-379	0,023320	5,64	0,122
miR-127-3p	0,001998	5,84	0,0339
miR-365	0,001980	8,05	0,0339
miR-30a*	0,001435	10,20	0,0339
miR-193b	0,002153	11,21	0,0339
miR-642	0,002023	12,34	0,0339
miR-483-5p	0,003274	16,96	0,0447
miR-149	0,001449	22,48	0,0339

miRNA	*P* value	Fold induction V *vs.* C	FDR
miR-375	0,029018	4,19	0,139
miR-370	0,014111	10,55	0,106
miR-654-3p	0,004255	12,12	0,0508
miR-193b*	0,000377	21,25	0,0163
miR-9	0,000019	64,59	0,0011
miR-129-3p	0,000002	343,25	0,000329
miR-935	0,000006	717,68	0,000554

*P* values are calculated using a random-variance model for univariate significance paired *t*-test, based on all available permutations. The maximum proportion of FDR was <0.2 (see Supplementary Methods). C: CD34^+^ control cells; V: VPA-treated CD34^+^ cells.

### Heart repair potency of control and HDACi-treated CD34^+^ cells

**Figure 6:**
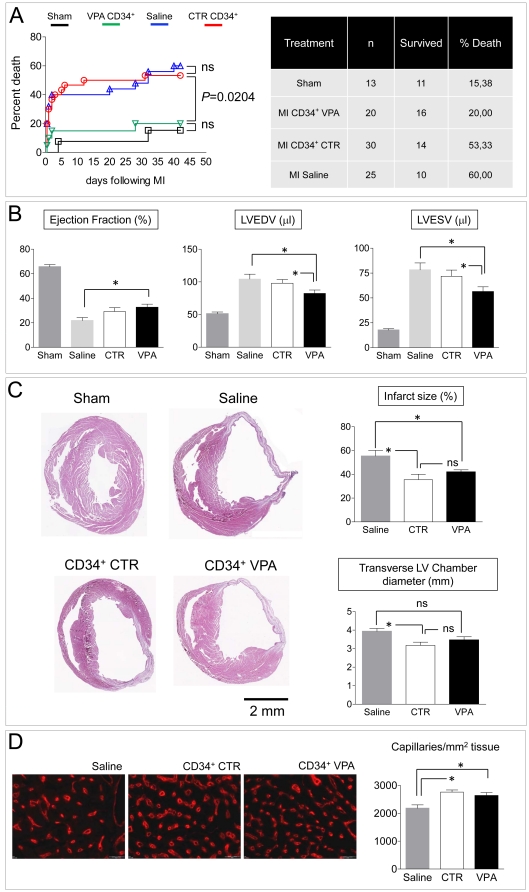
Effect of 7 days cultured CTR and VPA-treated CD34^+^ cells on survival, left ventricle function *neo*-vascularization and ventricular remodeling in an immunodeficient mouse model of myocardial infarction. (A) Mortality Kaplan-Meier curve of sham operated, saline-injected, control and VPA CD34^+^ cells-injected mice. The mortality in mice injected with VPA-treated CD34^+^ cells was not significantly different from that of sham operated mice (see enclosed table). Significance calculated by log-rank (Mantel-Cox) test. (B) Echocardiographic assessment of ejection fraction, left ventricular end-diastolic and end-systolic volumes showed an improved ventricular function in VPA treated CD34^+^ cells, but not in control CD34^+^ cells-injected animals. (C) Representative images of transversal sections of diastole-arrested hearts, used to evaluate the infarct scar size and LV morphometric values. * indicate *P*<0.05 by one way ANOVA with Newman Keuls post hoc analysis (n≥7). (D) Representative images of Rhodamine-labeled Griffonia simplicifolia Lectin 1 staining of LV histological sections for capillary density determination. The bar graph indicates the density of these vessels at the infarct border zone.

Seven days cultured CTR and VPA cells were injected in the ischemic left ventricle of SCID^beige^ mice 15 min after coronary artery ligation (CAL). Six weeks after CAL and cell injection, mice that survived and reached the end of the follow up period were analyzed for heart function and morphometry. Mortality data during the follow up period (Figure 6A) showed a dramatic difference between animals receiving VPA cells compared to those injected with CTR cells or saline. Echocardiography revealed that in animals injected with VPA cells Left Ventricular Ejection Fraction (LVEF) was significantly increased (33% improvement) compared with saline-injected mice; in addition VPA cells reduced Left Ventricular End Diastolic/Systolic volumes (LVEDV, LVESV) compared with CTR cells and saline (Figure 6B, Tables S1, Figure S9). To further assess efficacy of CTR and VPA cells, diastole-arrested hearts from sham operated, saline-, CTR- and VPA cells-injected mice were analyzed by histology to obtain LV morphometric parameters and by fluorescent lectin staining to measure capillary density (Figure 6C, D; Table S2). Surprisingly, VPA-treated cells neither reduced infarct size nor increased capillary density, compared with CTR cells.

Human cells survival at 6 wks after transplantation was evaluated using a qPCR method tailored to detect a human polymorphism with an efficiency of 0.1–1 equivalent human cell genome (0.6–6 pg) in an unrelated DNA mixture [21] (Figure S10). By this, it was found that survival of human cells *in vivo* was low (about 10^2^ cells out of the 1.5×10^5^ injected cells) and comparable in VPA and CTR cells-injected mice.

**Figure 7:**
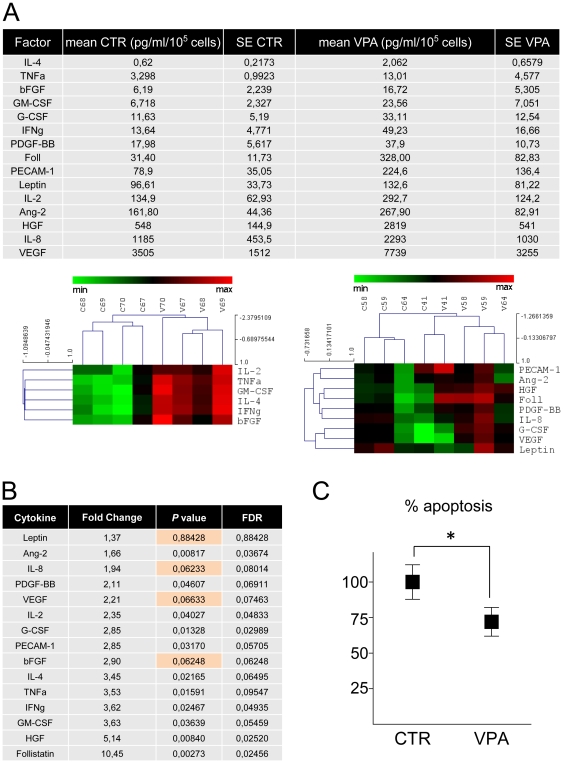
Enhanced cytokine release and higher hypoxia suppression by 7 days VPA-treated CD34^+^ cells conditioned medium. (A) Analysis of *pro*-inflammatory and *pro*-angiogenic factors present in the control and VPA-treated CD34^+^ cells secretome. Table shows means and standard error of each cytokine released in the culture supernatants. Heat maps indicate a treatment-related coherent upregulation of most of the secreted factors in four independent CD34^+^ cells samples. (B) Statistical analysis of the cytokine concentrations in culture supernatants by paired *t*-test. The fold change in the release of these cytokines from VPA-treated *vs.* CTR CD34^+^ cells is shown. With the exception of Leptin, IL-8, VEGF, and bFGF (orange color), all the other cytokines were significantly up-regulated in HDACi preconditioned cells, indicating an enhancement of their paracrine effect. (C) Effect of CTR and VPA CD34^+^ cells conditioned medium on rescue from apoptosis of HL-1 cardiomyocytes cell line exposed to hypoxia conditions. Data in the graph represent the percent variation in apoptotic death of HL-1 cells exposed to hypoxia in the presence of VPA treated CD34^+^ cells conditioned medium in comparison with medium conditioned by CTR cells; * indicate *P*<0.05 by paired t-test (n = 5).

It has been proposed that cytokines released from cells injected into the ischemic myocardium may interfere, at least in part, with progression of cell death due to hypoxia or may metabolically sustain ischemic myocardium [22]. To better characterize effects of VPA *vs.* CTR cells, release of inflammatory/*pro*-angiogenic cytokines was measured in conditioned medium. This tests showed a significantly higher expression of 11 out of 15 tested cytokines in VPA *vs.* CTR cells (Figure 7A, B). To assess whether modified cytokine expression in VPA *vs.* CTR-treated CD34^+^ cells protects against hypoxia-induced apoptosis, HL-1 cardiomyocyte-like cells [23] were exposed to medium conditioned from VPA and CTR cells in an *in vitro* hypoxia model. As shown in Figure 7C, VPA cells conditioned medium exerted higher protection of these cells from cell death consequent to hypoxia.

**Figure 8:**
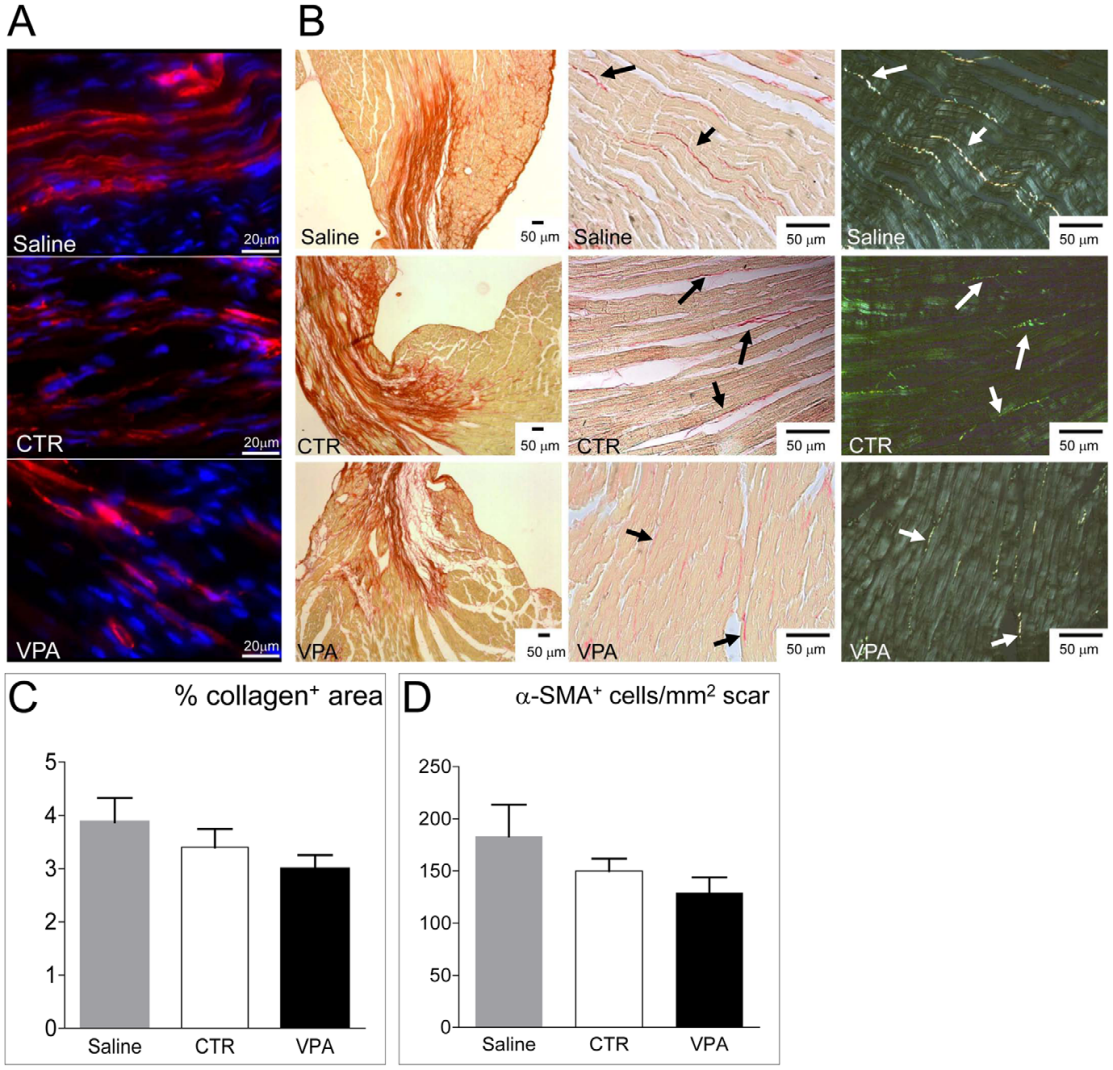
Effect of VPA and CTR cells on myocardial healing. (A) Representative images of α-SMA staining (red fluorescence) of the infarct zone to reveal the presence of myo-fibroblasts. (B) Representative low and high power views of picrosirius red staining of the myocardium to reveal the collagen deposition. Pictures on the right show polarized light imaging of the same microscopic fields in the center of the panel, to show that birefringence of collagen bundles was not affected by saline or CTR and VPA CD34^+^ cells treatments. (C–D) Quantification of Collagen deposition and myofibroblasts. Collagen data are shown as percentage of the areas containing collagen normalized to total area sections, while myofibroblasts were determined by counting the number of α-SMA^+^ cells in the infarct zone (n≥6). Statistical analysis of these data by one-way ANOVA with Newman-Keuls post-hoc test did not reveal differences between treatment groups, although lower amount of Collagen and smaller myofibroblasts number were found in VPA cells-injected mice.

Increased paracrine activity of injected cells might interfere with myocardial adverse remodeling following infarction. To verify this, collagen deposition and myofibroblasts number were determined by picrosirius red staining [24], [25] and α-smooth muscle actin (α-SMA) immunofluorescence [26], [27] in hearts injected with saline, CTR and VPA-treated cells. Results (Figure 8) showed that collagen deposition and presence of α-SMA^+^ myofibroblasts were (although not significantly) reduced in VPA cells-injected compared with CTR cells and saline-injected mice, suggesting lower adverse remodelling in hearts injected with VPA-treated cells.

## Discussion

### Direct and pleiotropic roles of HDACi blockade on CD34^+^ cells markers expression, cell cycle and phenotype

For years recognized as an useful treatment for inducing differentiation of transformed cells [28], histone deacetylase (HDAC) pharmacologic inhibition has been more recently indicated to promote self renewal for expanding the number of immature stem cells in bone marrow repopulation assays [14], [29], [30], [31], [32]. In line with these investigations, our experiments show that CD34^+^ cells were functionally modified by HDACi pretreatment and induced to maintain a “slow dividing” phenotype [33] compared with CTR cells. Increased immaturity and retarded growth were not the unique consequences of VPA treatment. In fact we observed: 1) negative and positive modulation of various stem cells and differentiated cells markers such as CD14, VEGFR2/KDR, CD31, CD38, CD48 and uptake ability of Ac-LDL (Figures 4, S7), 2) *de novo* expression of mesenchymal (CD90, CD130) and ECFCs/mature endothelial cells (CD146) [19], [34], [35] markers (Figures 4, S7) and, 3) enhanced ability of VPA CD34^+^ cells to produce ECFCs clones (Figure 4). Taken together, these results suggest that HDAC blockade causes a phenotype change of CD34^+^ cells due to activation of different, but likely interdependent, molecular pathways targeting at the same time cell proliferation and clonogenicity. It is striking that upregulation of stem cells markers (CD34, CD38, CD133 and KDR) occurred in concert with *de novo* expression of markers typical of differentiated (CD48, CD146) or mesenchymal (CD90, CD130) cells. This is in line with the evidence that exposure to drugs with genome-wide effects such as generalized histone acetylation has global consequences for cell (re)programming [36], [37], but also for fate determination of primitive cells [38], [39], [40].

### mRNA and miRNA profiling of HDACi-treated CD34^+^ cells reveals a streamlined epigenetic supervision of immature CD34^+^ cells phenotype

The clusterization of mRNAs and miRNAs expressed in VPA-treated cells (Figure 5) allowed clearly recognize specific gene expression signatures distinguishing them from CTR cells. For mRNAs it was possible to derive functional annotation charts describing occurrence of HDACi-regulated mRNAs into various BIOCARTA/KEGG categories (Table 3). This identified the canonical Wnt- (FZD1, WNT1), Notch- (NOTCH1, DLL3, DLL4) and Hedgehog-activated signaling (DHH, BMP2) as crucial nodes in the generation of the VPA-treated cells phenotype. This is important, as convergence of canonical-Wnt and Notch signaling is recognized to maintain primitive stem cells self renewal in the bone marrow stem cell niche [41], while Notch signaling has specific roles in EPCs *neo*-vascularization activity [42].

**Table 3:** Gene-enrichment analysis and functional annotation clustering of cell stem related genes in 7-days VPA-treated CD34^+^ cells.

CATEGORY	PATHWAY SPECS (TERM)	COUNT	%	*P*-value	GENES INVOLVED
BIOCARTA	h_ps1Pathway:Presenilin action in Notch and Wnt signaling	3	8,823529	0,009788	WNT1,FZD1,DLL1
BIOCARTA	h_cellcyclePathway:Cyclins and Cell Cycle Regulation	3	8,823529	0,0300447	CCNE1,CCND1,CCND2
BIOCARTA	h_wntPathway:WNT Signaling Pathway	3	8,823529	0,0300447	WNT1,CCND1,FZD1
KEGG_PATHWAY	hsa04110:Cell cycle	5	14,70588	0,0029498	CCNE1,CCND1,HDAC2,CCND2,CCNA2
KEGG_PATHWAY	hsa04330:Notch signaling pathway	3	8,823529	0,0219152	HDAC2,DLL3,DLL1
KEGG_PATHWAY	hsa04340:Hedgehog signaling pathway	3	8,823529	0,0303929	DHH,WNT1,BMP2
KEGG_PATHWAY	hsa04310:Wnt signaling pathway	4	11,76471	0,0365996	WNT1,CCND1,CCND2,FZD1
KEGG_PATHWAY	hsa04115:p53 signaling pathway	3	8,823529	0,04338	CCNE1,CCND1,CCND2

Our observations also reveal a possible interplay between positive and negative stimuli controlling the immature and slow dividing phenotype of VPA-treated cells. In fact, VPA caused downregulation of CDH1 (E-cadherin) and upregulation of FDZ1, WNT1, CCND1, DLL1/3 and mir-9 in VPA-treated CD34^+^ cells, suggesting enhancement of β-catenin-mediated transactivation and positive effects on CD34^+^ cells proliferation [43]. On the other hand, HDAC inhibition also determined coherent up- or down-modulation of several miRNAs directly involved in positive (e.g. mir-129-3p, mir-193b, mir-370) or negative (e.g. mir-196b, mir-335, mir-370) control of cell cycle [44], [45], [46], [47], [48], [49], thus suggesting the existence of an epigenetically regulated negative loop protecting CD34^+^ cells from unrepressed cellular growth, and reinforcing the anti proliferative effect exerted by small cyclin/CDK inhibitors such as p14^ARF^, p16^INK4^ and p21^Cip1/Waf1^ gene products (Figure 2E). Negative effects on cell proliferation may also depend on an HDACi-related modification of the DNA methylation status. This is suggested by the finding that VPA induced mir148, a miRNA targeting DNMT3b methyltransferase, and by the evidence that several of the miRNAs over- or under-expressed in VPA-treated cells are transcriptionally regulated by CpG islands methylation [50], [51], [52].

### Cardiac protection by HDACi-treated CD34^+^ cells is independent of CD34^+^ cells regeneration enhancement

HDAC inhibitors potently reduce *in vitro* and *in vivo* angiogenesis by repressing the ability of mature endothelial cells to form vascular structures [53] or by inhibiting EPCs maturation into endothelium [54]. In addition, HDAC genes targeting (e.g. SIRT-1 or HDAC4,7) impairs vascular development [55], [56], [57]. Therefore, preconditioning with HDACi should reduce and not increase CD34^+^ cells *pro*-angiogenic function into ischemic tissues. Injection of CTR and VPA-treated cells in the ischemic heart (Figure 6) showed a remarkable effect of HDACi cellular preconditioning on the survival of treated mice. This was associated to a significant enhancement of cardiac function but, surprisingly, neither corresponded to a more efficient reduction of the infarct size, nor to a significant improvement of myocardial tissue regeneration compared with CTR cells (Figure 6, Tables S1, S2). Furthermore, as shown by survival of similar, but low, amount of living human cells in the host myocardium (Figure S10), this effect was not due to a higher engraftment ability of VPA-treated cells.

How to reconcile these data? Our results call for a generalized increase of CD34^+^ cells cardioprotection ability or an improved “paracrine effect” that may sustain cardiac contractility or interfere with myocardial cells apoptosis as short times after infarction. In support of this hypothesis is the finding that VPA-treated cells showed an enhanced expression of several *pro*-angiogenic/*pro*-inflammatory cytokines at mRNA (FGF2, CXCL12/SDF-1) or protein (bFGF, IL-8, VEGF, Ang-2, IFN-γ, TNFα) levels, of cardioprotective factors such as Follistatin [58] and of resident progenitors activating factors such as HGF [59]. Therefore, generalized upregulation of all these gene products in the secretome of CD34^+^ cells may lead to functional preservation of the left ventricle by sustaining cardiac metabolism and contractility, even in the absence of a net reduction of infarct size compared with control CD34^+^ cells (discussed in [22]). The *in vitro* data showing significantly higher rescue of HL-1 cardiac myocytes from hypoxia in the presence of VPA cells conditioned medium (Figure 7C) supports this conclusion.

Presence of bone marrow-derived or resident mesenchymal cells-derived myofibroblasts during early stages after infarction is an important component of the innate immunity response to ischemia [60], [61], [62]. By contrast, chronic permanence of these cells after MI [63], or their increased number due to aging [64], is associated to enhanced fibrosis and ventricular dysfunction. In the present study, specific experiments to assess the dynamics of myofibroblasts accumulation at early time points after MI were not performed. However, the lower amount of collagen and the reduced myo-fibroblasts number observed at six weeks in the infarct zone (Figure 8), suggest that injection of VPA-treated cells partially prevented chronic scarring of the myocardial tissue, thus justifying the observed reduction of ventricular dysfunction. Again, this may depend on enhanced cytokine secretion observed in VPA-treated *vs.* CTR cells.

In summary, the data shown in the present study identify a possible novel strategy to enhance the protective ability of human CD34^+^ cells against the consequences of myocardial infarction. Future studies will be needed to clarify the molecular mechanisms underlying the interplay between VPA-treated CD34^+^ cells and the ischemic micro-environment, thus allowing to draw a clear scenario for their enhanced repair function.

## Materials and Methods

### Ethics statement

Collection of cord blood samples was performed upon written consent and on a voluntary basis. An Institutional Review Board formal approval for cord blood collection at Melzo Hospital was obtained to this aim (**December 12, 2008; authorization no 843**). All animal studies conformed to national and international “Guide for the Care and Use of Laboratory Animals” and the Helsinki declaration. The protocols were reviewed and approved by the Animal Care and Use Committee at Mario Negri Institute, and by the Italian Health Ministry (**protocol 0804 base1**); they were further compliant with European directives and guidelines **(Legislative Decree September 19, 1994, n. 626 (89/391/CEE, 89/654/CEE, 89/655/CEE, 89/656/CEE, 90/269/CEE, 90/270/CEE, 90/394/CEE, 90/679/CEE).**

### Cord blood collection, expansion and phenotype analysis of CD34^+^ cells

Isolation and culture of CD34^+^ cells were performed using a magnetic beads-based method (MINI-MACS) and a serum-free expansion medium [11] as detailed in Materials and Methods S1. At the indicated time points, cells were incubated with suitable combinations of monoclonal antibodies recognizing stem cell-specific, endothelial, hematopoietic or mesenchymal specific markers (1–10 µg/ml final concentration). Cell growth was assessed by incubating cells with CFSE or Propidium Iodide staining and by Ki-67 specific antibodies. To assess stem cell phenotype of VPA *vs.* control treated CD34^+^ cells, Rhodamine123 extrusion by MDR-1 gene product and ALDH enzyme activity were measured by flow cytometry. Methodology and reagents used for these tests are described in the Materials and Methods S1.

### ECFC Clonogenic expansion

ECFC clonogenic assay was performed as already described [20]. Briefly, fresh, CTR and VPA-treated CD34^+^ cells were plated at low density into collagen-coated dishes. Clusters of rapidly expanding endothelial-like cells were counted and further expanded for phenotype and functional analyses, as described in Materials and Methods S1.

### Chromatin immunoprecipitation

Chromatin immunoprectipitation was performed as described in Nanni et al., 2009 [65], using ChIP-IT Express Enzymatic kit, according manufacture's instruction (Active Motif). Enrichment of DNA sequences associated to segments of the CD34 gene promoter was tested using a quantitative method based on real-time PCR. Further details are provided in Materials and Methods S1.

### Transcript and miRNA profiling

For detection of stem cells-associated transcripts, total RNA was extracted from control *vs.* VPA-treated cells after which the RT^2^ Profiler PCR Array system (human stem cell-specific card, catalogue PAHS-405E,Version 4.26; SABiosciences) was used. For microRNAs profiling, total RNA was extracted from high throughput-sorted CD34^bright^ cells by flow cytometry. Profiling was performed by TaqMan Human MicroRNA A and B Arrays, version 2.0 (Applied Biosystems, USA). Both procedures were performed in a 7900HT (Applied Biosystems, USA) fast real time cycler. A complete description of cell sorting and RNA extraction procedures as well as of raw data (Tables S3, S4 and S5) normalization and statistical handling is provided in Materials and Methods S1.

### Animal studies

A SCID^beige^ mouse model of myocardial infarction by permanent left coronary artery ligation was used. Control and VPA-treated cells (1.5×10^5^ cells/animal) were injected in the left ventricle (LV) at the infarct border zone 15^min^ after CAL. As controls, sham operated and saline-injected animals were used. After a 6 weeks follow up period, heart functional analyses were performed by transthoracic ultra-imaging echocardiography (VisualSonics, Vevo 770), followed by mice sacrifice for histological analysis and immunofluorescence. Further details are provided in the Materials and Methods S1.

### Cytokine, chemokine and growth factor detection; apoptosis in HL-1 cell line

Bio-Plex assay (Bio-Rad Laboratories, Italy), a bead-based multiplex immunoassay, was used to quantify cytokines, chemokines and growth factors secreted in culture supernatant by Control and VPA-treated cells. Results are expressed as pg/ml/10^5^ cells. Conditioned medium from these cells was also used to assess protection from hypoxia-induced apoptosis in HL-1 cardiomyocyte cell line [23]. Further details are provided in Materials and Methods S1.

### Cardiac engraftment analysis

Analysis of CD34^+^ cells engraftment was performed using a qPCR method allowing detection of a human-specific polymorphism in an excess mouse DNA [21]. Further details are provided in Materials and Methods S1.

### Data analysis

All results are expressed as mean ± standard error. Statistical significance was determined with paired or unpaired Student's *t*-test, one-way Anova with Newman-Keuls *post*-hoc analysis or Two-ways Anova with Bonferroni post-hoc analysis.

## Supporting Information

Figure S1 **Morphology of CD34^+^ cells cultured in the absence (Control) and the presence of 2.5 mM VPA at 7 days of culture.** Note the presence of blast-colonies indicative of rapid proliferation/differentiation events in control condition (green arrows) and that of elongated and mild adherent cells (red arrows) in VPA-treated cells.
(TIF)

Figure S2 **Effect of increasing doses of VPA and TSA on side scatter (SSC) increase of CD34^+^ cells at 7 days of culture.** Note progressive shift toward high SSC values of HDACi-treated cells (open black histogram) overlaid onto control cells plots. Statistical evaluation by Kolomogorov-Smirnov test showed significant divergence (D≥0.20) of the histogram plots for TSA at 25 ng/ml and VPA at 2.5 and 5 mM concentrations.
(TIF)

Figure S3 **CD133 antigen expression profile in control and VPA-treated cells at 5 days in culture.** The three regions corresponding to CD133^neg^, CD133 ^dim^ and CD133^bright^ cells are shown.
(TIF)

Figure S4 **(A) Mathematical deconvolution by ModFit software of CFSE profiles in three independent experiments of CD34^+^ cells culture in the presence and the absence of VPA.** The different generations are indicated by different colours. Histogram plots on the right indicate cells distribution in the various cellular generations at days 7 of culture. (B) Example of CFSE profile in the CD34^bright^ gating of seven days cultured CTR and VPA cells. It is evident that VPA-treated cells were shifted toward the right side of the plot, indicating brighter CFSE fluorescence and consistent growth retardation. Inset shows the result of Kolmogorov-Smirnov test, indicating a statistically significant (D>0.20) divergence of the two curves.
(TIF)

Figure S5 **Experimental flowchart describing the main actions and time points of the phenotype and** ***in vivo*** **function analyses of control and VPA-treated CD34^+^ cells.**
(TIF)

Figure S6 **Stem cell activity in CD34^+^ cells after isolation from cord blood.** Contour plots are designed as in Figure 4.
(TIF)

Figure S7 **Marker analysis in CTR and VPA CD34^+^ cells at 14 days of culture.** As observed at day 7, a number of stem cell (CD34, CD133 and KDR), endothelial (CD31, CD146, LDL uptake) and mesenchymal (CD90) markers were upregulated.
(TIF)

Figure S8 **CD34 expression profile in CD34^+^ cells cultured for 7 days in the presence or the absence of VPA.** Contour plots on the top show CD34 expression in cultured cells before high throughput sorting by flow cytometry; plots on the bottom indicate the purity control after sorting.
(TIF)

Figure S9 **Parasternal long axis (pLAX, left) and short-axis (pSAX, right) views by echocardiography.** Left side of the figure: end diastolic (left) and end-systolic frames (right) of sham operated or, Saline, CTR CD34^+^ cells and VPA-treated cells-injected mice. Ao: aorta, LA: left atrium, LV: left ventricle, MV: mitral valve, IVS: inter-ventricular septum, PW: posterior wall. Arrows indicate the extension of the infarcted wall. Note the increased wall thinning, chamber dilatation, and systolic expansion of saline injected compared to CD34^+^ cells (CTR or VPA) injected mice. Right side of the figure. M-mode echocardiogram of the left ventricle of the same mice shown in pLAX view. LV: left ventricle, ASW: anteroseptal wall, IPW: inferior-posterior wall. Arrows indicate the infarcted wall.
(TIF)

Figure S10 **Determination of the human cells survival in the mouse heart.** qPCR was performed by a Taqman amplification protocols to detect the human SNP C/T (rs6625561 Reference: NCBI SNP). Amplification plots in the upper right show the threshold cycle of increasing (10× higher at each dilution) amounts of human DNA into a fixed amount of mouse DNA, while the other amplification plots are derived from amplification of DNA extracted by saline injected or cells injected mice. Graph on the bottom shows the approximate linearity between threshold cycle and 10× increasing amount human of human DNA into the fixed mouse DNA excess. Red and green lines indicate the CT values for amplification of heart DNA from one mouse receiving CTR cells and another receiving VPA-treated cells.
(TIF)

Table S1 **Mean±SE of various heart functional parameters derived from echocardiography.** HR: heart rate, LVIDd: left ventricular end diastolic diameter, LVIDs: left ventricular end systolic diameter; SF: shortening fraction, LVEF: left ventricular ejection fraction, LVEDV: left ventricular end diastolic volume, LVESV: left ventricular end systolic volume, AWThd: anterior wall diastolic thickness, PWThd: posterior wall diastolic thickness. LVEF, LVEDV and LVESV were calculated from PLAX view.
(DOCX)

Table S2 **Morphometric data.** HW: heart weight. BW: body weight. MI: Myocardial Infarction. VTh: Ventricular Thickness.
(DOCX)

Table S3 **Ct raw data of RT^2^ Profiler PCR Arrays (stem cells related transcripts).** HGDC: Human Genomic DNA contamination Control; RTC = Reverse Transcription Control; PPC = Positive PCR Control.
(DOCX)

Table S4 **Ct raw data of TaqMan Human MicroRNA Arrays Card A.** ath-miR159a: negative control.
(DOCX)

Table S5 **Ct raw data of TaqMan Human MicroRNA Arrays Card B.** ath-miR159a: negative control.
(DOCX)

Materials and Methods S1 Detailed description of experimental procedures.
(DOCX)
