# Supplementary figures and images for: Histone Deacetylase Inhibition Enhances Self Renewal and Cardioprotection by Human Cord Blood-Derived CD34+ Cells
Source: PLoS One. 2011 Jul 18;6(7):e22158. doi: 10.1371/journal.pone.0022158 (PMC3138768; doi:10.1371/journal.pone.0022158)

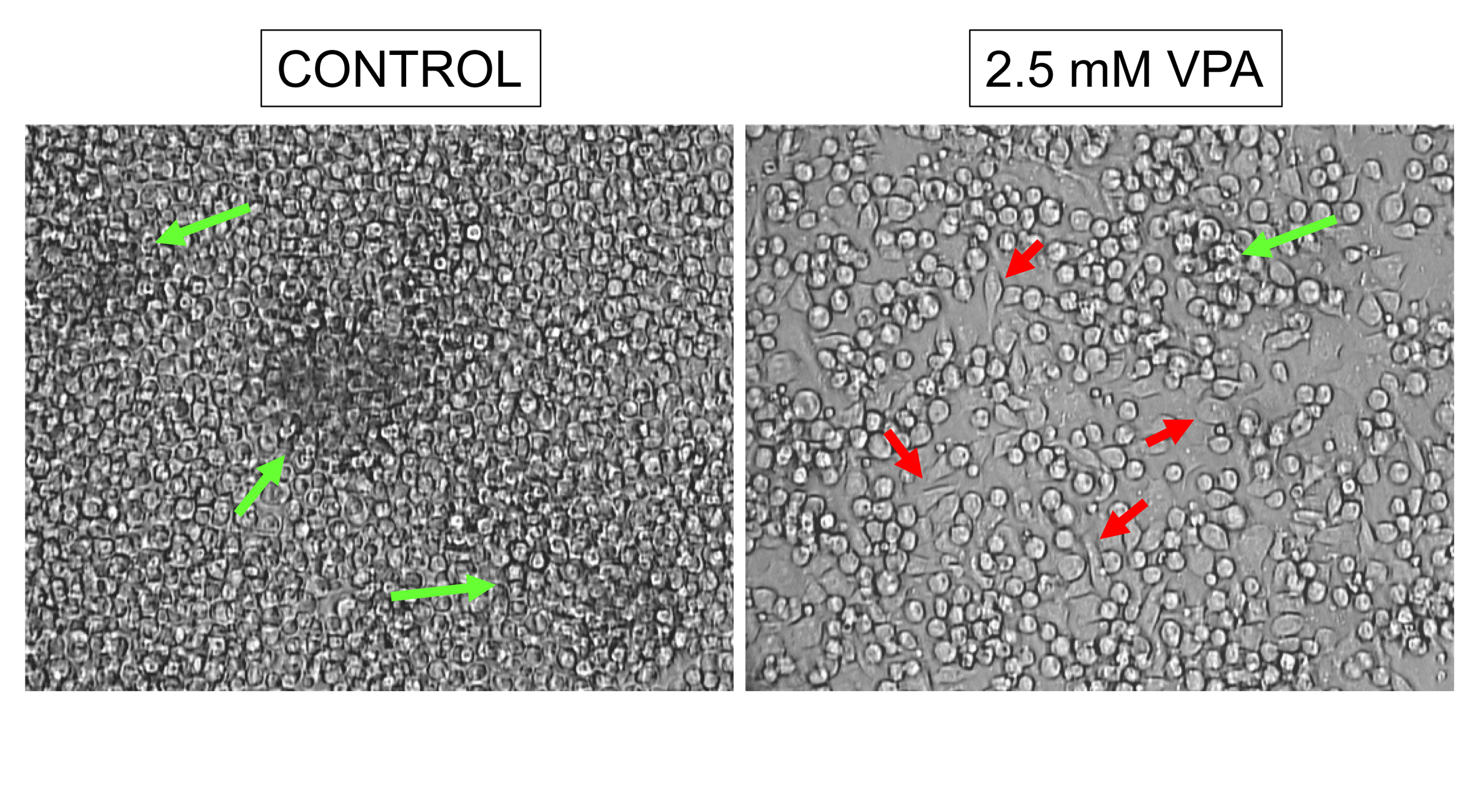

Supplement: Figure S1 — Morphology of CD34+ cells cultured in the absence (Control) and the presence of 2.5 mM VPA at 7 days of culture. Note the presence of blast-colonies indicative of rapid proliferation/differentiation events in control condition (green arrows) and that of elongated and mild adherent cells (red arrows) in VPA-treated cells. (TIF) [file pone.0022158.s001.tif]

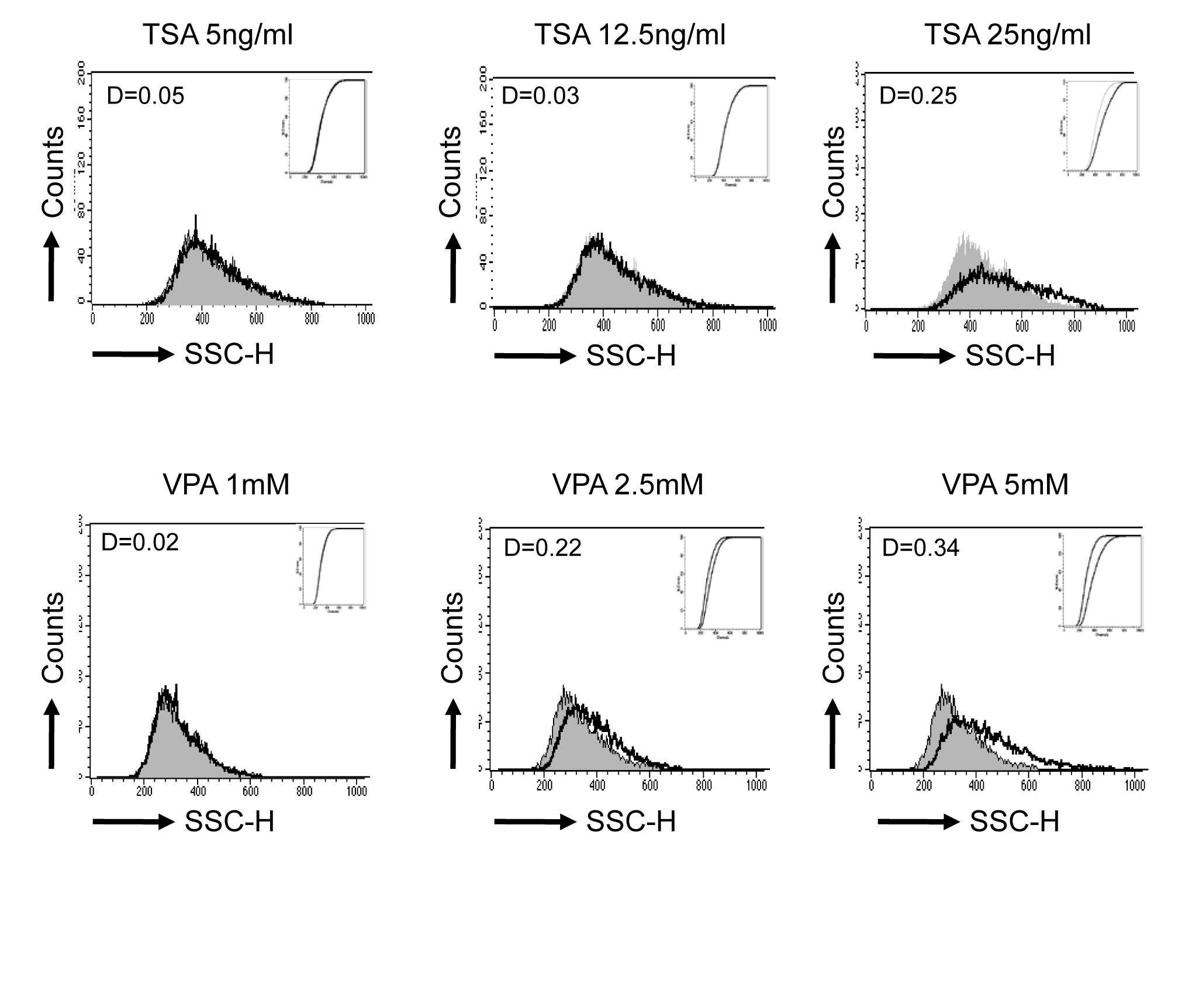

Supplement: Figure S2 — Effect of increasing doses of VPA and TSA on side scatter (SSC) increase of CD34+ cells at 7 days of culture. Note progressive shift toward high SSC values of HDACi-treated cells (open black histogram) overlaid onto control cells plots. Statistical evaluation by Kolomogorov-Smirnov test showed significant divergence (D≥0.20) of the histogram plots for TSA at 25 ng/ml and VPA at 2.5 and 5 mM concentrations. (TIF) [file pone.0022158.s002.tif]

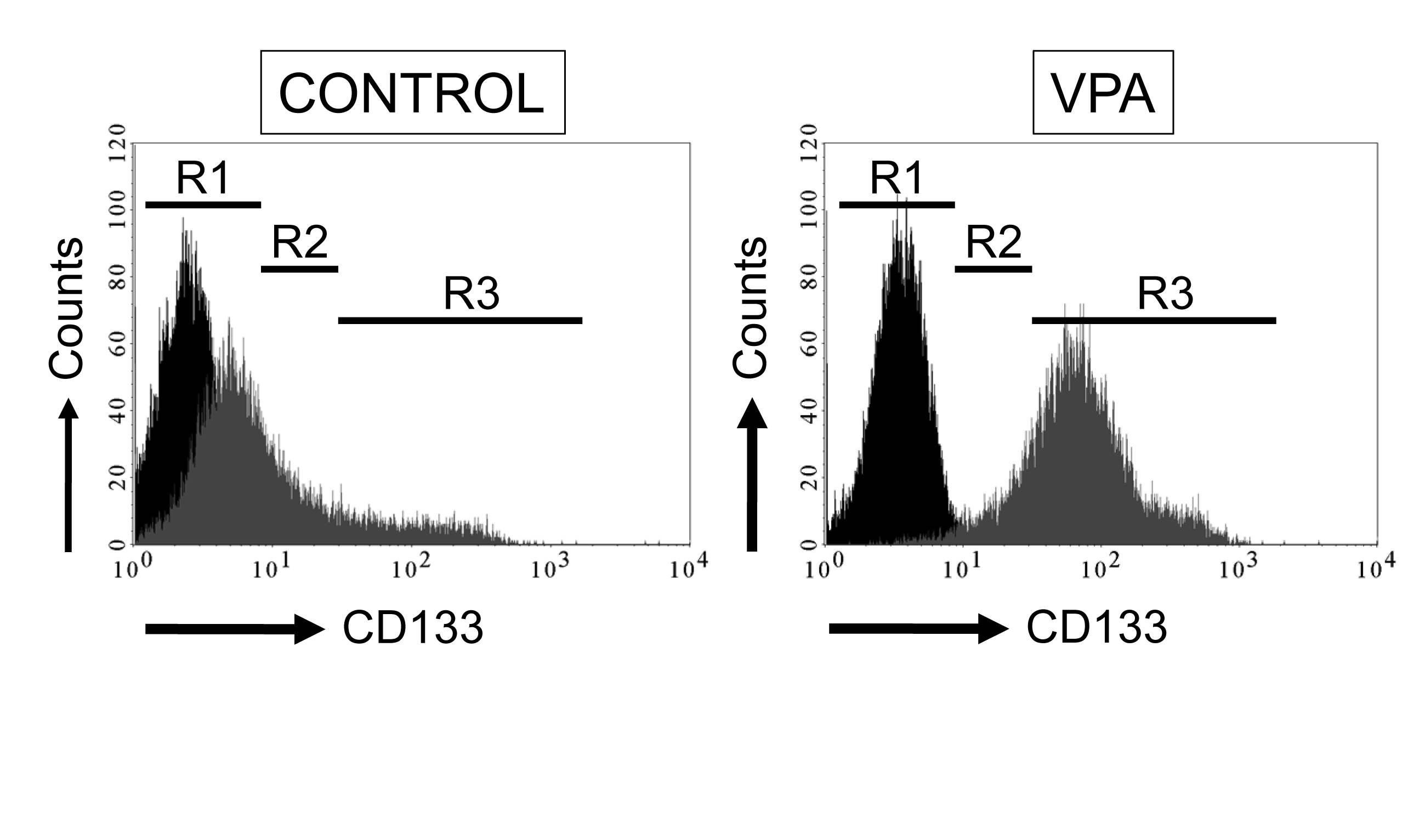

Supplement: Figure S3 — CD133 antigen expression profile in control and VPA-treated cells at 5 days in culture. The three regions corresponding to CD133neg, CD133 dim and CD133bright cells are shown. (TIF) [file pone.0022158.s003.tif]

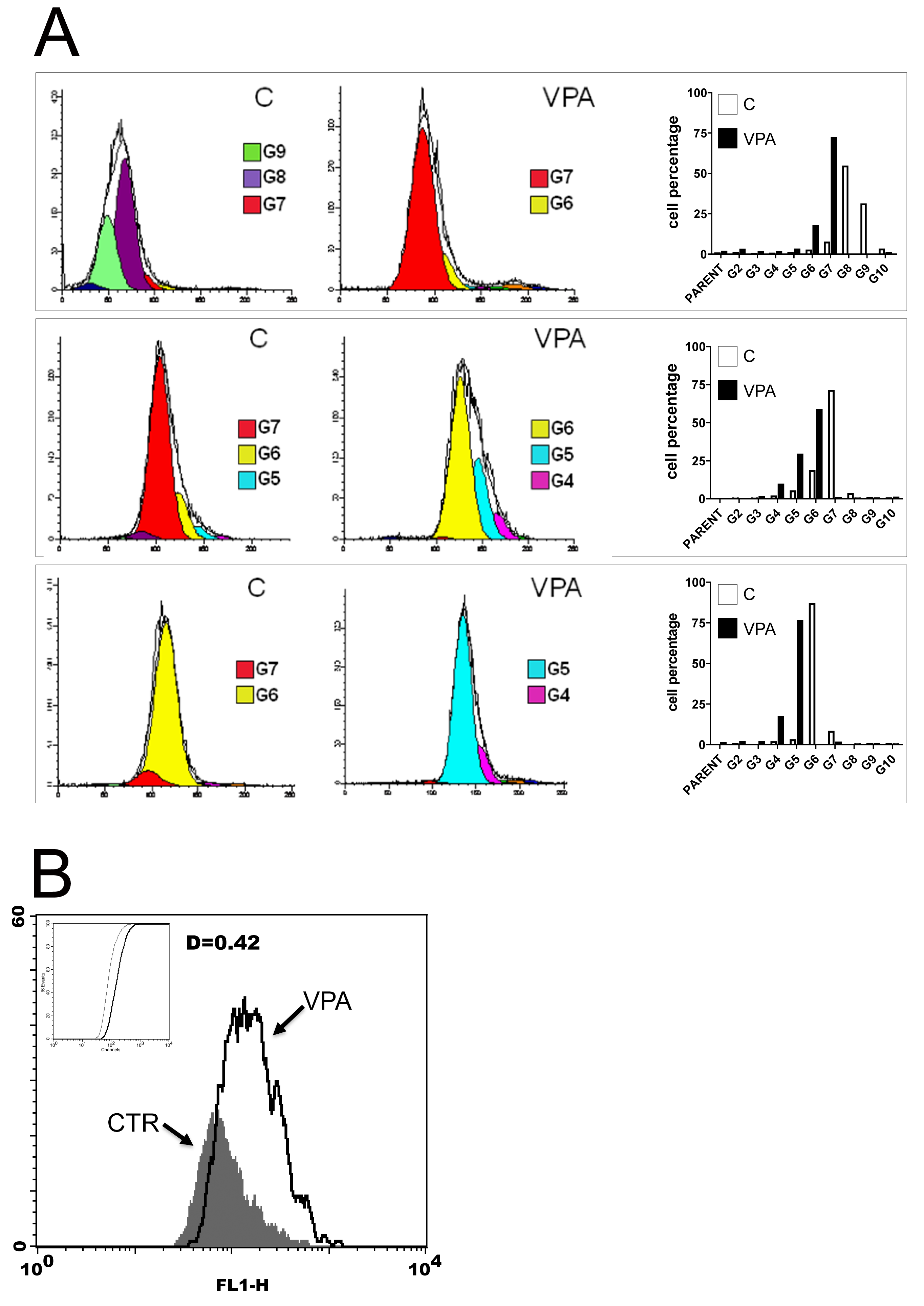

Supplement: Figure S4 — (A) Mathematical deconvolution by ModFit software of CFSE profiles in three independent experiments of CD34+ cells culture in the presence and the absence of VPA. The different generations are indicated by different colours. Histogram plots on the right indicate cells distribution in the various cellular generations at days 7 of culture. (B) Example of CFSE profile in the CD34bright gating of seven days cultured CTR and VPA cells. It is evident that VPA-treated cells were shifted toward the right side of the plot, indicating brighter CFSE fluorescence and consistent growth retardation. Inset shows the result of Kolmogorov-Smirnov test, indicating a statistically significant (D>0.20) divergence of the two curves. (TIF) [file pone.0022158.s004.tif]

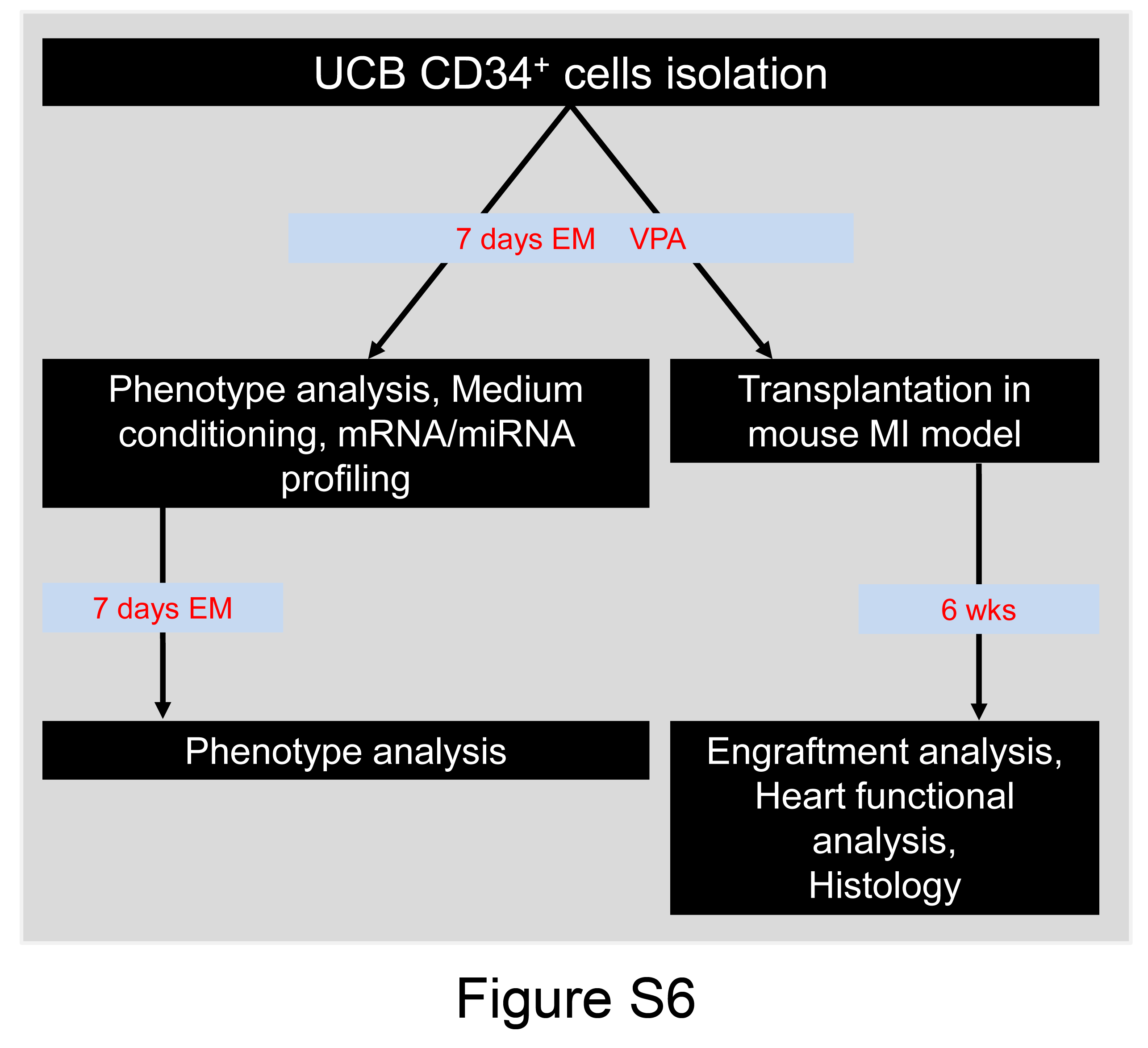

Supplement: Figure S5 — Experimental flowchart describing the main actions and time points of the phenotype and in vivo function analyses of control and VPA-treated CD34+ cells. (TIF) [file pone.0022158.s005.tif]

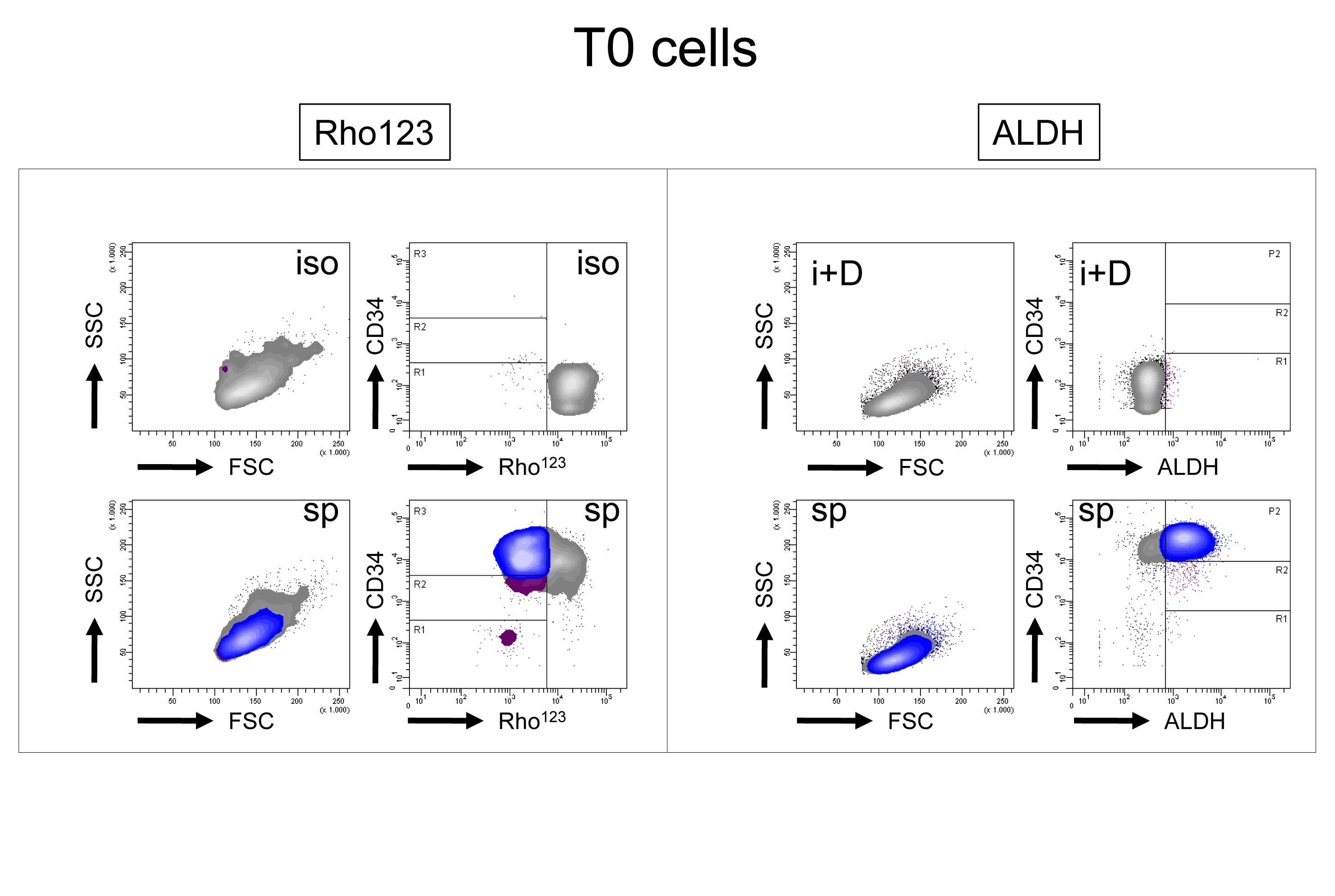

Supplement: Figure S6 — Stem cell activity in CD34+ cells after isolation from cord blood. Contour plots are designed as in Figure 4. (TIF) [file pone.0022158.s006.tif]

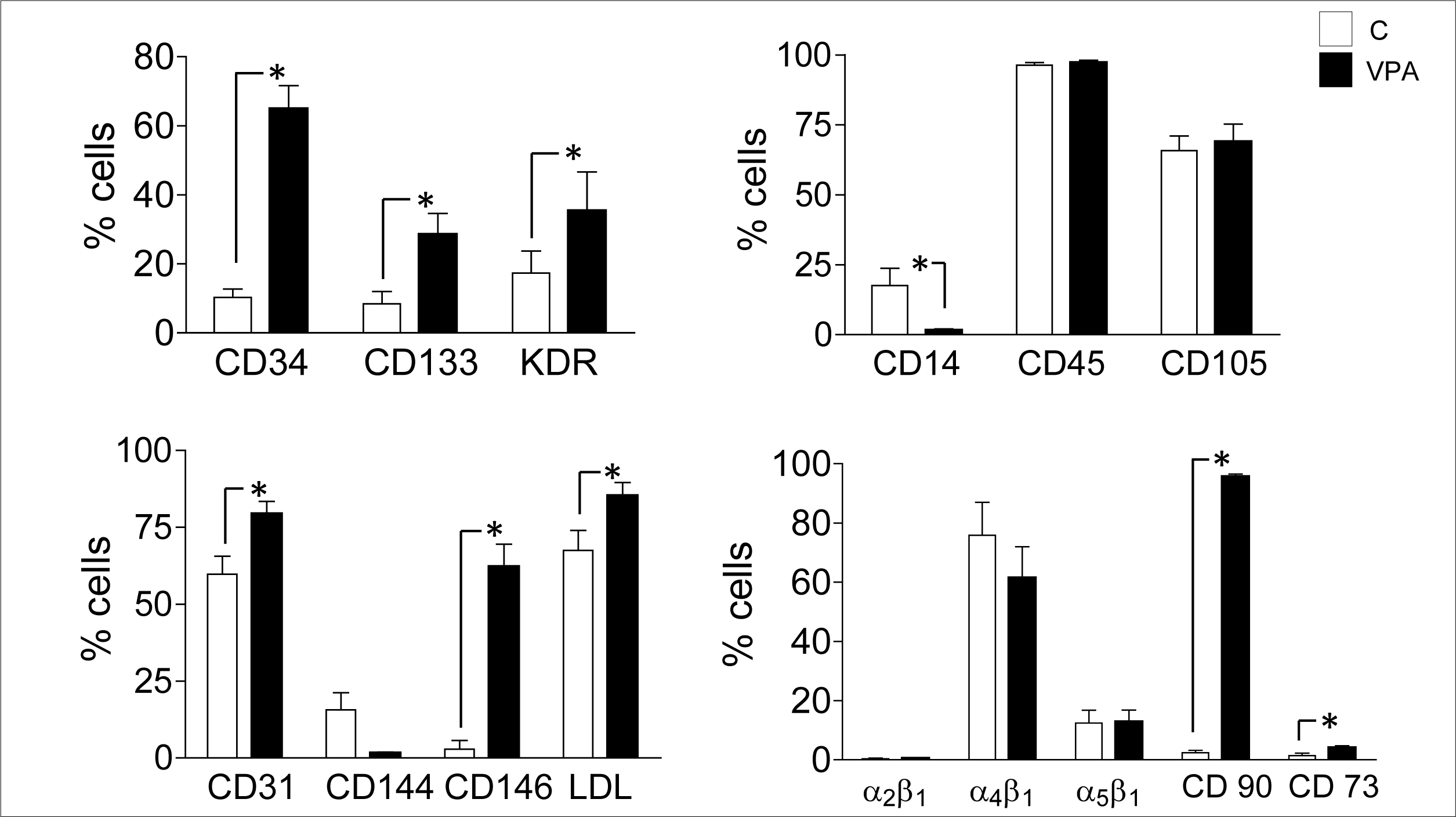

Supplement: Figure S7 — Marker analysis in CTR and VPA CD34+ cells at 14 days of culture. As observed at day 7, a number of stem cell (CD34, CD133 and KDR), endothelial (CD31, CD146, LDL uptake) and mesenchymal (CD90) markers were upregulated. (TIF) [file pone.0022158.s007.tif]

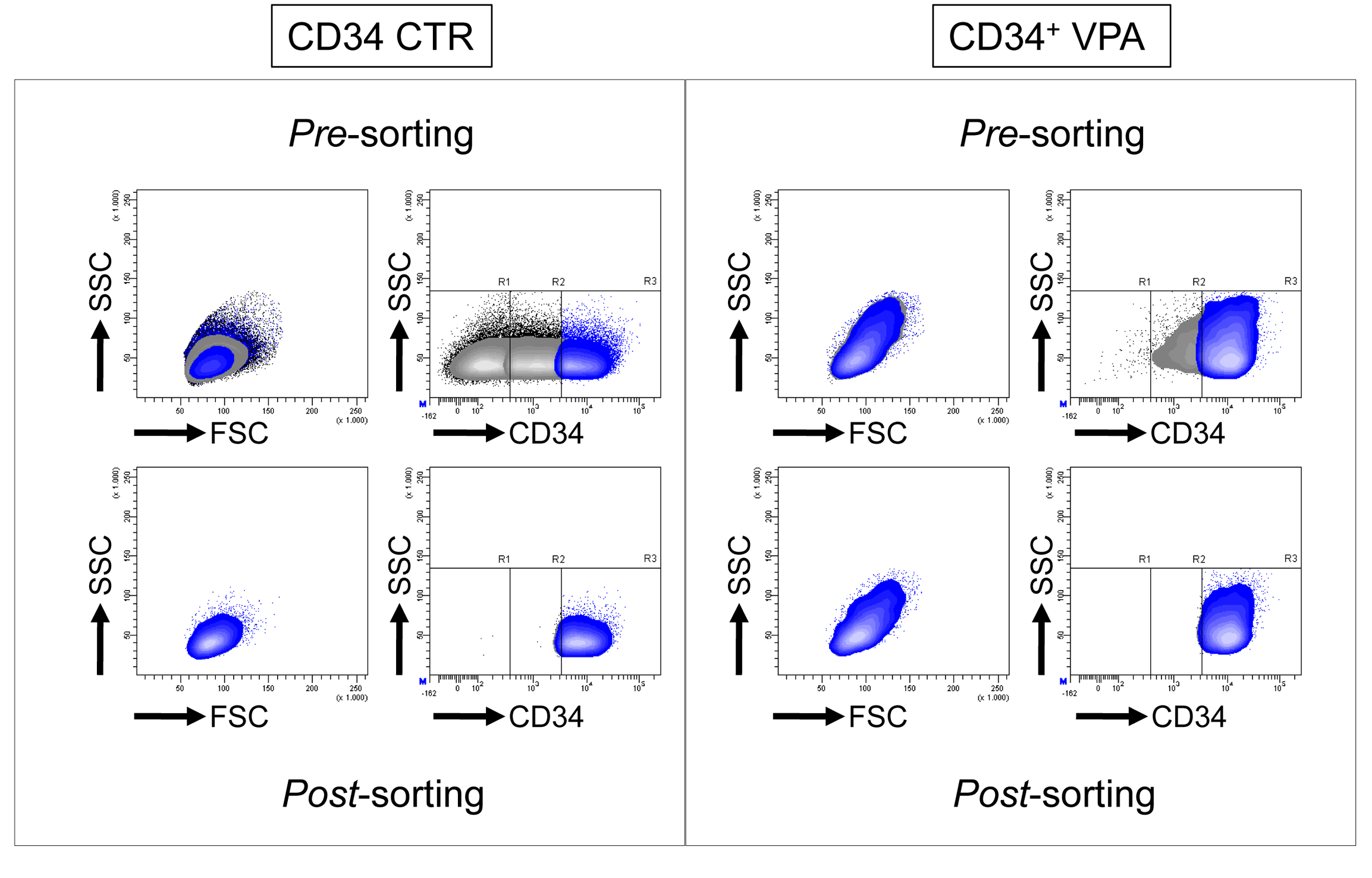

Supplement: Figure S8 — CD34 expression profile in CD34+ cells cultured for 7 days in the presence or the absence of VPA. Contour plots on the top show CD34 expression in cultured cells before high throughput sorting by flow cytometry; plots on the bottom indicate the purity control after sorting. (TIF) [file pone.0022158.s008.tif]

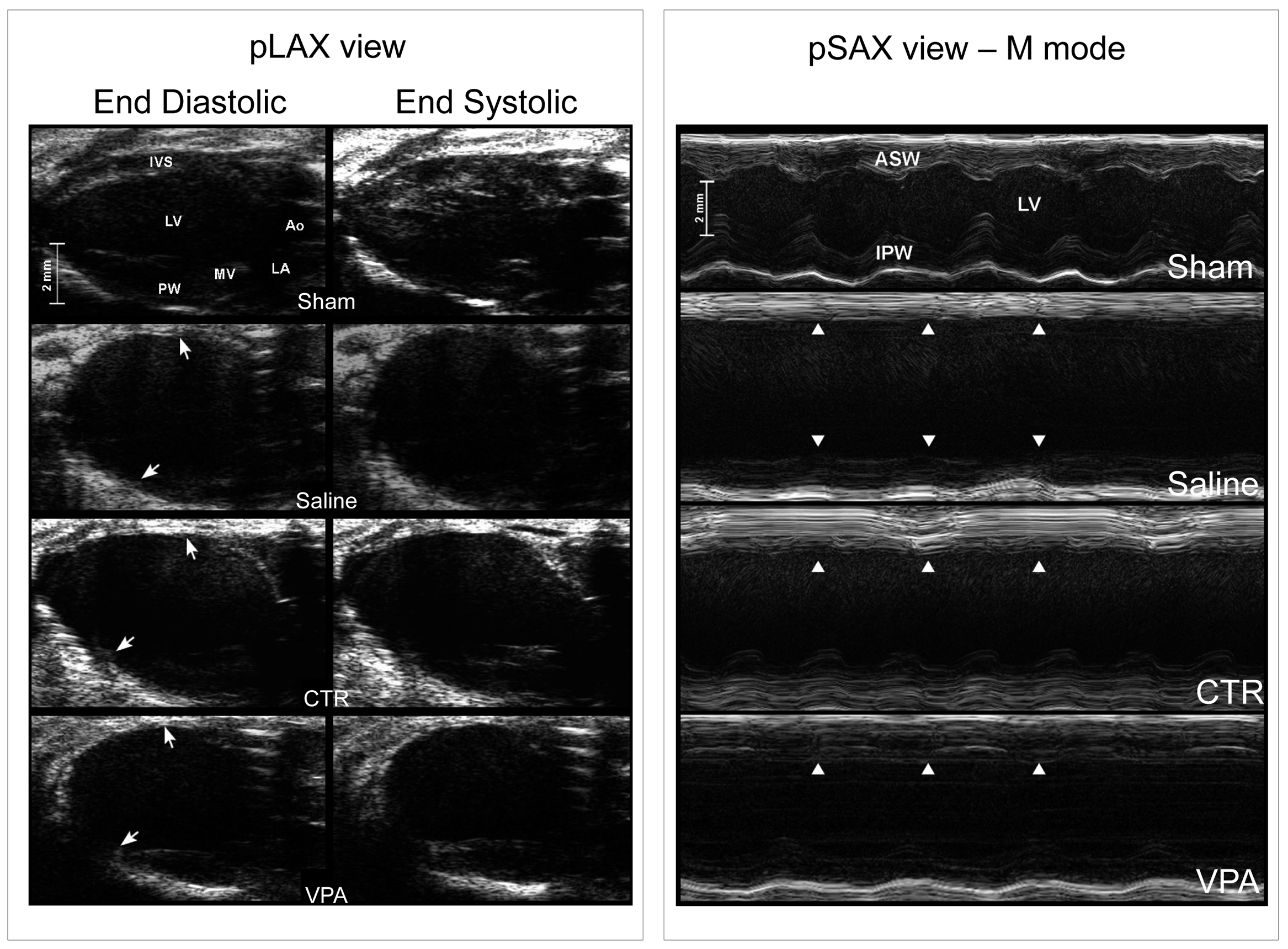

Supplement: Figure S9 — Parasternal long axis (pLAX, left) and short-axis (pSAX, right) views by echocardiography. Left side of the figure: end diastolic (left) and end-systolic frames (right) of sham operated or, Saline, CTR CD34+ cells and VPA-treated cells-injected mice. Ao: aorta, LA: left atrium, LV: left ventricle, MV: mitral valve, IVS: inter-ventricular septum, PW: posterior wall. Arrows indicate the extension of the infarcted wall. Note the increased wall thinning, chamber dilatation, and systolic expansion of saline injected compared to CD34+ cells (CTR or VPA) injected mice. Right side of the figure. M-mode echocardiogram of the left ventricle of the same mice shown in pLAX view. LV: left ventricle, ASW: anteroseptal wall, IPW: inferior-posterior wall. Arrows indicate the infarcted wall. (TIF) [file pone.0022158.s009.tif]

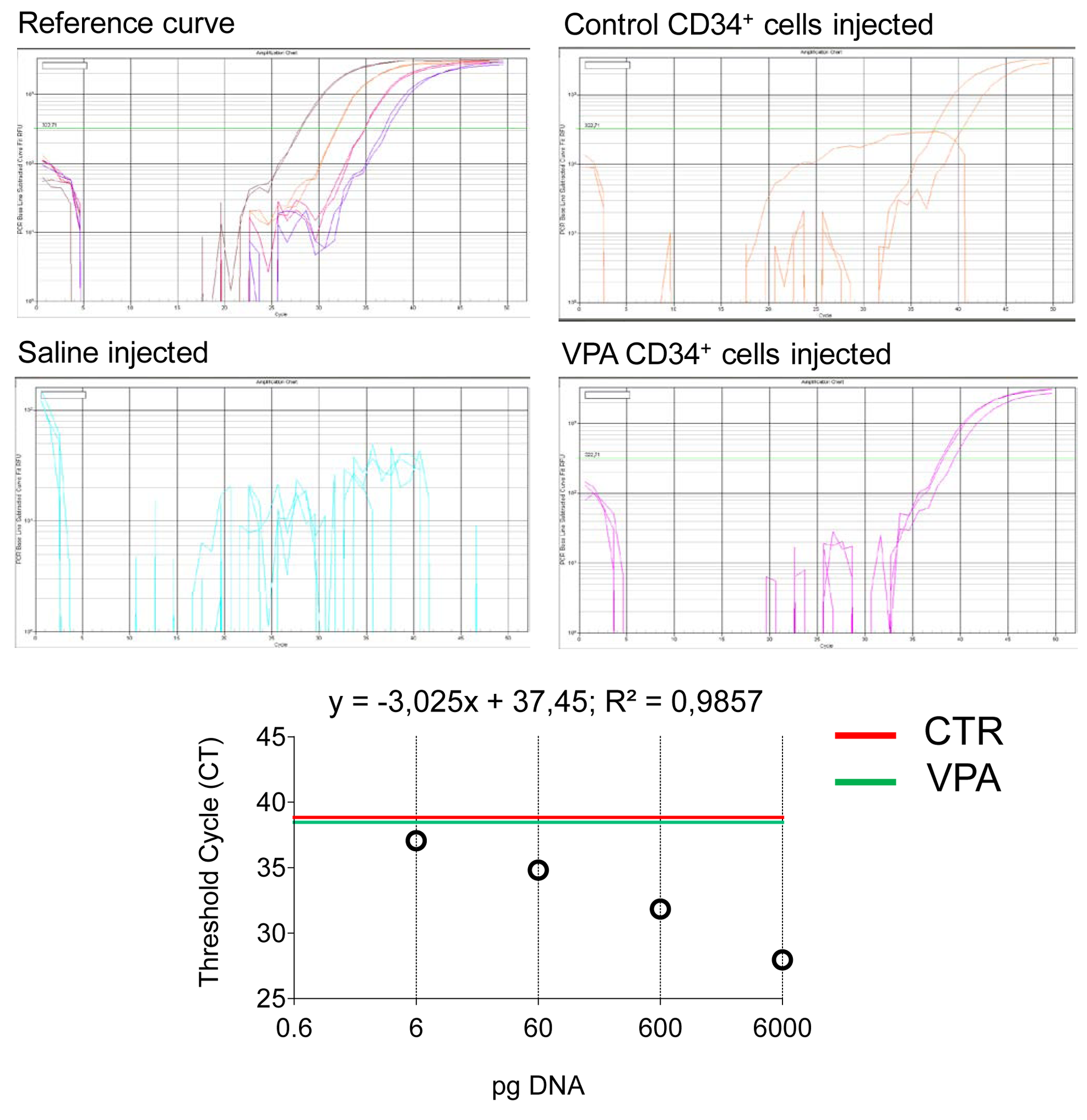

Supplement: Figure S10 — Determination of the human cells survival in the mouse heart. qPCR was performed by a Taqman amplification protocols to detect the human SNP C/T (rs6625561 Reference: NCBI SNP). Amplification plots in the upper right show the threshold cycle of increasing (10× higher at each dilution) amounts of human DNA into a fixed amount of mouse DNA, while the other amplification plots are derived from amplification of DNA extracted by saline injected or cells injected mice. Graph on the bottom shows the approximate linearity between threshold cycle and 10× increasing amount human of human DNA into the fixed mouse DNA excess. Red and green lines indicate the CT values for amplification of heart DNA from one mouse receiving CTR cells and another receiving VPA-treated cells. (TIF) [file pone.0022158.s010.tif]
