# Supplementary material for: Histone Deacetylase Inhibition Enhances Self Renewal and Cardioprotection by Human Cord Blood-Derived CD34+ Cells
Source: PLoS One. 2011 Jul 18;6(7):e22158. doi: 10.1371/journal.pone.0022158 (PMC3138768; doi:10.1371/journal.pone.0022158)
Supplement: Table S1 — Mean±SE of various heart functional parameters derived from echocardiography. HR: heart rate, LVIDd: left ventricular end diastolic diameter, LVIDs: left ventricular end systolic diameter; SF: shortening fraction, LVEF: left ventricular ejection fraction, LVEDV: left ventricular end diastolic volume, LVESV: left ventricular end systolic volume, AWThd: anterior wall diastolic thickness, PWThd: posterior wall diastolic thickness. LVEF, LVEDV and LVESV were calculated from PLAX view. (DOCX) [file pone.0022158.s011.docx]

| Parameter | Sham | MI Saline | MI CD34 ^+^ CTR | MI CD34^+^ VPA |
| --- | --- | --- | --- | --- |
|  | n=12 | n=9 | n=12 | n=15 |
| HR (bpm) | 391±14 | 457±14 | 443±23 | 434±16 |
| LVIDd (mm) | 3.5±0.1 | 5.1±0.3 | 4.7±0.3 | 4.6±0.2 |
| LVIDs (mm) | 2.2±0.1 | 4.2±0.6 | 3.8±0.4 | 3.5±0.2 |
| SF (%) | 38.1±1.5 | 19.4±3.7 | 19.7±2.6 | 22.1±1.8 |
| LVEF (%) | 65.7±1.6 | 21.8±2.3 | 29.2±3.0 | 32.5±2.6 |
| LVEDV (ml) | 51±3 | 104±7 | 98±6 | 82±5 |
| LVESV (ml) | 18±1 | 78±7 | 72±6 | 56±5 |
| AWThd (mm) | 0.85±0.03 | 0.61±0.10 | 0.58±0.07 | 0.65±0.05 |
| PWThd (mm) | 0.93±0.03 | 0.78±0.05 | 0.85±0.04 | 0.93±0.04 |

**TABLE S1.**
