# Supplementary material for: Histone Deacetylase Inhibition Enhances Self Renewal and Cardioprotection by Human Cord Blood-Derived CD34+ Cells
Source: PLoS One. 2011 Jul 18;6(7):e22158. doi: 10.1371/journal.pone.0022158 (PMC3138768; doi:10.1371/journal.pone.0022158)
Supplement: Table S2 — Morphometric data. HW: heart weight. BW: body weight. MI: Myocardial Infarction. VTh: Ventricular Thickness. (DOCX) [file pone.0022158.s012.docx]

| Parameter | Sham | MI Saline | MI CD34 ^+^ CTR | MI CD34^+^ VPA |
| --- | --- | --- | --- | --- |
|  | n=10 | n=7 | n=9 | n=14 |
| BW(g) | 25.4±0.6 | 23.9±0.5 | 23.1±0.91 | 22.9±0.57 |
| HW(mg) | 102.8±4.9 | 139.8±17.7 | 108.8±4.57 | 116.8±4.86 |
| HW/BW(mg/g) | 4.1±0.2 | 5.9±0.8 | 4.8±0.3 | 5.1±0.2 |
| VTh (mm) | 0.90±0.05 | 0.47±0.06 | 0.71±0.07 | 0.60±0.05 |
| Septum Thickness(mm) | 1.10±0.04 | 1.10 ±0.28 | 0.98±0.08 | 1.02±0.04 |
| VTh/LV Chamber Radius | 0.86±0.10 | 0.24 ± 0.04 | 0.47±0.06 | 0.37±0.10 |
| Septum Thickness/LV Chamber radius | 1.04±0.10 | 0.54±0.10 | 0.63±0.07 | 0.61±0.10 |
| Transverse Chamber Diameter (mm) | 2.3±0.2 | 3.9±0.2 | 3.2±0.2 | 3.5±0.1 |
| LV Chamber Area(mm²) | 4.2±0.5 | 12.3±1.0 | 8.1±0.9 | 9.7±0.5 |

**TABLE S2**
