# Supplementary material for: Histone Deacetylase Inhibition Enhances Self Renewal and Cardioprotection by Human Cord Blood-Derived CD34+ Cells
Source: PLoS One. 2011 Jul 18;6(7):e22158. doi: 10.1371/journal.pone.0022158 (PMC3138768; doi:10.1371/journal.pone.0022158)
Supplement: Table S3 — Ct raw data of RT2 Profiler PCR Arrays (stem cells related transcripts). HGDC: Human Genomic DNA contamination Control; RTC = Reverse Transcription Control; PPC = Positive PCR Control. (DOCX) [file pone.0022158.s013.docx]

**TABLE S3**

| **Gene Name** | **C147** | **C148** | **C149** | **C91** | **V147** | **V148** | **V149** | **V91** |
| --- | --- | --- | --- | --- | --- | --- | --- | --- |
| **ABCG2** | 32.7436 | und | und | und | 32.6532 | 31.6995 | 31.2034 | 31.6206 |
| **ACTC1** | 36.5098 | und | und | und | und | und | und | und |
| **ADAR** | 24.0110 | 24.1521 | 23.6903 | 24.6134 | 23.8197 | 23.7976 | 23.4489 | 24.3465 |
| **ACAN** | und | und | und | und | und | und | und | und |
| **ALDH1A1** | 24.8432 | 24.3439 | 24.5989 | 26.0030 | 21.6783 | 21.4288 | 21.4779 | 22.2352 |
| **ALDH2** | 25.7333 | 25.8882 | 25.6018 | 25.6251 | 25.5346 | 25.2947 | 25.1613 | 25.6578 |
| **ALPI** | und | und | und | und | und | und | und | und |
| **APC** | 29.5574 | 29.8713 | 29.1326 | 30.6087 | 29.9947 | 30.0037 | 29.1284 | 32.5814 |
| **ASCL2** | 30.0109 | 30.3289 | 29.5354 | 30.9482 | 28.3535 | 27.9806 | 28.2705 | 30.8504 |
| **AXIN1** | 25.4675 | 25.7925 | 25.3652 | 25.9404 | 26.1531 | 25.9708 | 26.1903 | 26.5832 |
| **BGLAP** | 29.2288 | 28.9751 | 28.8816 | 29.8181 | 29.8063 | 29.6652 | 29.2654 | 30.5003 |
| **BMP1** | 27.6440 | 27.5925 | 27.2823 | 27.5249 | 27.6392 | 27.0136 | 27.0195 | 27.2706 |
| **BMP2** | und | 35.1940 | und | und | 33.4637 | 33.4793 | 34.0478 | 32.5090 |
| **BMP3** | und | und | und | und | und | und | 36.2266 | 33.6167 |
| **BTRC** | 27.5109 | 27.8455 | 28.2229 | 28.6474 | 27.9439 | 27.9980 | 28.5670 | 30.8730 |
| **CCNA2** | 22.8704 | 22.7421 | 23.0869 | 23.5805 | 25.1429 | 24.1333 | 24.6814 | 25.5612 |
| **CCND1** | 28.6459 | 28.8591 | 28.8550 | 28.8561 | 27.1316 | 27.8198 | 26.8899 | 26.9947 |
| **CCND2** | 22.6920 | 22.8178 | 23.0930 | 23.1155 | 22.3578 | 22.5763 | 22.4266 | 22.5969 |
| **CCNE1** | 26.6634 | 26.8723 | 26.5809 | 26.6620 | 25.6558 | 25.5599 | 25.6233 | 25.3139 |
| **CD3D** | 30.9614 | 29.9230 | 29.8786 | 28.6059 | 29.6246 | 29.4809 | 30.9449 | 28.7603 |
| **CD4** | 27.5917 | 27.7192 | 26.6928 | 27.5527 | 26.3184 | 26.8947 | 26.2492 | 27.3510 |
| **CD44** | 23.3029 | 23.1684 | 22.7405 | 23.1859 | 22.3293 | 22.3668 | 22.2190 | 21.8758 |
| **CD8A** | und | 36.2326 | 34.2681 | und | 31.6816 | 31.8304 | 31.6908 | 31.5000 |
| **CD8B** | 34.5907 | 30.9078 | 31.2620 | 31.2242 | 31.4291 | 30.3327 | 30.8807 | 29.2473 |
| **CDC2** | 23.0257 | 23.1555 | 23.2353 | 23.7462 | 24.5567 | 23.5099 | 23.9692 | 25.2196 |
| **CDC42** | 22.3009 | 22.3685 | 22.0081 | 22.3649 | 22.3491 | 22.4064 | 22.2529 | 22.1866 |
| **CDH1** | 27.2814 | 27.2297 | 26.9547 | 27.6629 | 31.8499 | 30.2005 | 29.2372 | und |
| **CDH2** | 34.2441 | 35.3316 | und | 32.7074 | 29.8253 | 29.7539 | 31.7387 | 30.3674 |
| **COL1A1** | 33.6079 | 33.3936 | 33.2470 | 33.6094 | 28.9739 | 27.8950 | 28.7999 | 29.2817 |
| **COL2A1** | und | 32.6040 | 33.8888 | und | 29.8216 | 30.2175 | 31.4895 | 32.2520 |
| **COL9A1** | und | und | und | und | und | und | und | und |
| **CTNNA1** | 23.7499 | 23.7888 | 24.2302 | 24.3852 | 23.8488 | 23.8307 | 23.8213 | 24.5829 |
| **CXCL12** | 30.9897 | 30.9110 | 31.7889 | 33.6272 | 27.2846 | 27.2526 | 26.1910 | 27.3755 |
| **DHH** | 31.8786 | 35.0147 | 34.4188 | und | 29.9795 | 29.8302 | 28.9330 | 31.6459 |
| **DLL1** | 31.9386 | 31.2435 | 30.9528 | und | 28.7259 | 27.6316 | 28.5640 | 30.6087 |
| **DLL3** | und | 38.6521 | 38.2065 | 39.1993 | 31.8010 | 32.3190 | 31.5951 | 31.5700 |
| **DTX1** | 33.7442 | 31.6398 | 30.9507 | 31.6228 | 29.4465 | 31.3018 | 29.7568 | 30.9067 |
| **DTX2** | 26.4728 | 26.7674 | 26.1663 | 26.6711 | 26.4402 | 26.5135 | 26.3867 | 27.4639 |
| **DVL1** | 26.5944 | 26.9180 | 26.3597 | 27.3697 | 27.1588 | 26.9532 | 26.6097 | 27.2230 |
| **EP300** | 25.2362 | 25.4407 | 25.1308 | 25.5509 | 25.5460 | 25.4380 | 25.3217 | 25.2721 |
| **FGF1** | und | 34.3111 | und | 33.6090 | und | und | und | und |
| **FGF2** | 30.6250 | 30.7531 | 31.7854 | 31.8580 | 29.1909 | 28.5888 | 29.5100 | 28.9535 |
| **FGF3** | und | und | und | und | und | und | und | und |
| **FGF4** | und | und | und | und | und | und | und | und |
| **FGFR1** | 32.7323 | 32.2547 | 31.4438 | 31.6148 | 30.5806 | 30.4393 | 29.2735 | 30.6687 |
| **FOXA2** | und | und | und | und | 32.6270 | 34.2473 | und | und |
| **FRAT1** | 27.3260 | 27.6570 | 26.9588 | 27.2932 | 27.3149 | 27.5014 | 27.0029 | 27.6398 |
| **FZD1** | 28.8724 | 28.4389 | 29.5687 | 30.5256 | 27.5114 | 27.2877 | 27.5151 | 28.4670 |
| **KAT2A** | 25.3539 | 25.2728 | 25.2135 | 25.5682 | 25.8005 | 25.4826 | 25.4087 | 25.8403 |
| **GDF2** | und | 34.0054 | und | und | 31.1727 | 31.2898 | 32.2643 | 30.6060 |
| **GDF3** | und | und | 33.2163 | und | 29.3747 | 29.4179 | 30.9101 | 30.5554 |
| **GJA1** | 30.7754 | nd | 32.8911 | 31.8376 | 32.2993 | 30.5869 | 30.6610 | 29.6242 |
| **GJB1** | 39.0875 | und | 37.1660 | und | und | 38.0721 | und | 34.6307 |
| **GJB2** | 31.4980 | 33.2717 | 32.2113 | und | und | 32.8897 | 32.4986 | 33.2497 |
| **HDAC2** | 34.0620 | 33.8121 | 34.1359 | und | 33.4787 | 32.7009 | 32.3735 | 33.8871 |
| **HSPA9** | 22.2227 | 22.2020 | 22.2076 | 22.4439 | 22.4092 | 22.2688 | 22.2914 | 22.5328 |
| **IGF1** | 33.8427 | und | und | und | 34.5084 | und | und | und |
| **PDX1** | und | und | und | und | und | 32.3921 | und | und |
| **ISL1** | und | und | und | und | und | und | und | und |
| **JAG1** | 28.7364 | 29.1723 | 27.3248 | 27.8146 | 29.9621 | 29.2215 | 29.5107 | 28.9274 |
| **KRT15** | 31.8157 | und | 33.8285 | 34.4080 | 34.0208 | 34.6002 | und | und |
| **MME** | und | 29.8791 | 30.6411 | 31.8526 | und | 33.7056 | 33.7579 | 32.2054 |
| **MSX1** | und | 35.2062 | und | 36.5778 | 28.8620 | 28.9725 | 28.7344 | 28.6460 |
| **MYC** | 23.6069 | 23.8701 | 23.5721 | 24.1927 | 24.4213 | 24.2449 | 24.2450 | 25.5346 |
| **MYOD1** | und | und | und | und | und | und | und | und |
| **MYST1** | 24.5445 | 24.5900 | 24.3603 | 24.7793 | 25.6102 | 25.4306 | 25.1952 | 25.6503 |
| **MYST2** | 24.1750 | 24.3970 | 24.2465 | 24.3928 | 25.2807 | 24.9659 | 25.1879 | 24.5421 |
| **NCAM1** | und | und | und | und | 28.1540 | 27.6972 | 27.5601 | 28.7736 |
| **NEUROG2** | und | und | und | und | und | und | und | und |
| **NOTCH1** | 28.4346 | 28.7174 | 28.1375 | 28.9555 | 28.3375 | 27.8847 | 28.3314 | 28.9876 |
| **NOTCH2** | 24.7706 | 25.1852 | 24.5549 | 25.8756 | 25.1901 | 24.7906 | 24.6204 | 25.8347 |
| **NUMB** | 24.6224 | 24.3822 | 24.5544 | 25.2620 | 24.6056 | 24.6290 | 24.7053 | 25.3293 |
| **SIGMAR1** | 26.4140 | 26.7778 | 25.7497 | 25.9775 | 26.5549 | 26.5199 | 25.9625 | 26.7389 |
| **PARD6A** | 28.7829 | 29.7348 | 29.2992 | 29.6136 | 27.7781 | 27.5954 | 27.7908 | 28.4005 |
| **PPARD** | 25.5006 | 25.4101 | 25.6665 | 25.8005 | 25.6048 | 25.0402 | 24.7016 | 26.3385 |
| **PPARG** | 32.1017 | und | 32.2151 | 37.1552 | und | und | 33.4032 | und |
| **RB1** | 25.8989 | 26.2022 | 27.3121 | 26.6783 | 26.6612 | 26.1570 | 26.1700 | 27.2724 |
| **S100B** | 24.7873 | 24.8533 | 24.5440 | 25.3748 | 27.8319 | 28.8767 | 27.9181 | 28.6080 |
| **SOX1** | und | und | und | und | und | und | 34.6835 | und |
| **SOX2** | und | und | und | und | 33.8549 | 31.6096 | und | und |
| **T** | 35.8128 | 36.5360 | 34.1932 | 36.2480 | 33.2887 | 31.6051 | 31.9066 | 31.7889 |
| **TERT** | 30.4086 | 29.7775 | 29.9363 | 30.8742 | 32.9237 | 32.4106 | 32.8652 | 33.7610 |
| **TUBB3** | 29.5707 | 28.7050 | 29.2493 | 30.4298 | 28.1764 | 27.4118 | 27.8678 | 28.1841 |
| **WNT1** | und | und | 39.5138 | und | 31.9792 | 31.9933 | 30.7340 | 33.5488 |
| **B2M** | 19.9044 | 19.5860 | 18.6822 | 19.3068 | 19.4685 | 19.2963 | 19.2138 | 19.4209 |
| **HPRT1** | 23.4748 | 23.5119 | 23.3530 | 24.1022 | 24.2588 | 23.8540 | 23.5240 | 23.9781 |
| **RPL13A** | 19.2831 | 19.4148 | 19.1267 | 19.5031 | 19.5172 | 19.7204 | 19.4457 | 19.5630 |
| **GAPDH** | 18.4734 | 18.6324 | 18.2024 | 18.7244 | 18.4407 | 19.1415 | 18.3070 | 18.8241 |
| **ACTB** | 19.6632 | 19.2031 | 19.1887 | 19.5110 | 19.6063 | 19.4858 | 19.1759 | 19.2241 |
| **HGDC** | 39.2225 | 35.8050 | 38.6796 | und | 35.9945 | 35.0007 | und | 36.2049 |
| **RTC** | 21.1141 | 21.1659 | 20.7030 | 20.7364 | 20.8760 | 20.9620 | 21.2199 | 20.9583 |
| **RTC** | 20.9512 | 21.3663 | 20.6466 | 20.7453 | 20.8870 | 20.9176 | 21.1600 | 20.9978 |
| **RTC** | 20.9910 | 21.2074 | 20.6495 | 20.7322 | 20.9047 | 21.1196 | 21.1708 | 21.1068 |
| **PPC** | 16.5704 | 17.1586 | 16.7835 | 16.8950 | 17.1742 | 16.8806 | 16.6428 | 16.8236 |
| **PPC** | 16.6584 | 17.2878 | 16.3467 | 16.7672 | 16.9582 | 16.8917 | 16.7930 | 16.6815 |
| **PPC** | 16.5646 | 17.0122 | 16.3138 | 16.6341 | 16.9048 | 16.7338 | 16.7275 | 16.7299 |
