# Supplementary material for: Histone Deacetylase Inhibition Enhances Self Renewal and Cardioprotection by Human Cord Blood-Derived CD34+ Cells
Source: PLoS One. 2011 Jul 18;6(7):e22158. doi: 10.1371/journal.pone.0022158 (PMC3138768; doi:10.1371/journal.pone.0022158)
Supplement: Table S4 — Ct raw data of TaqMan Human MicroRNA Arrays Card A. ath-miR159a: negative control. (DOCX) [file pone.0022158.s014.docx]

**Table S4**

| **Name** | **C64** | **C68** | **C70** | **C71** | **V64** | **V68** | **V70** | **V71** |
| --- | --- | --- | --- | --- | --- | --- | --- | --- |
| **ath-miR159a** | und | und | und | und | und | und | und | und |
| **let-7a** | und | 28.8032 | und | und | und | und | und | und |
| **let-7b** | 26.2168 | 24.9376 | 25.1570 | 25.6107 | 25.7211 | 26.0099 | 26.4446 | 25.6369 |
| **let-7c** | 26.6680 | 26.5039 | 27.2475 | 28.3488 | 28.8339 | 26.5173 | 27.3065 | 26.7440 |
| **let-7d** | 26.6249 | 25.4767 | 27.0201 | 26.4406 | 28.4298 | 26.3282 | 26.4092 | 25.9262 |
| **let-7e** | 23.7567 | 22.5161 | 23.8419 | 24.2678 | 24.7894 | 23.5779 | 23.7100 | 22.7307 |
| **let-7f** | und | und | und | und | und | und | und | und |
| **let-7g** | 26.6744 | 25.7392 | 27.4359 | 26.5713 | 28.6910 | 26.8319 | 27.0650 | 25.7325 |
| **MammU6** | 19.5608 | 17.4362 | 15.7474 | 13.9599 | 13.9149 | 17.8467 | 17.9783 | 14.3902 |
| **MammU6** | 20.0072 | 17.4348 | 16.2836 | 14.5567 | 14.7224 | 17.3992 | 18.0193 | 14.4794 |
| **MammU6** | 19.2972 | 17.4204 | 16.0860 | 14.3355 | 13.6328 | 17.5040 | 18.0553 | 14.3984 |
| **MammU6** | 19.8847 | 17.0018 | 16.2910 | 14.3376 | 14.5372 | 17.8065 | 17.4933 | 14.3838 |
| **miR-1** | und | und | und | und | und | und | und | 34.6481 |
| **miR-100** | 29.1198 | 28.6150 | und | 32.2173 | 28.5588 | 28.5881 | 29.1589 | 30.1287 |
| **miR-101** | 31.6398 | und | und | und | und | 31.3480 | und | 32.9112 |
| **miR-103** | 27.0066 | 27.4645 | 29.4486 | 29.0871 | 30.4383 | 28.2722 | 29.1523 | 28.2077 |
| **miR-105** | und | und | und | und | und | und | und | und |
| **miR-106a** | 21.5148 | 20.4185 | 22.1907 | 20.8006 | 23.0118 | 22.2085 | 22.3578 | 20.8209 |
| **miR-106b** | 25.2342 | 27.6665 | 27.4521 | 27.6706 | 27.0365 | 26.7961 | 27.7990 | 27.6825 |
| **miR-107** | und | 32.4508 | und | 34.8761 | und | 33.6733 | und | 32.3936 |
| **miR-10a** | 27.2445 | 27.0952 | 28.4574 | 29.2896 | 27.7806 | 26.7898 | 27.1918 | 26.0692 |
| **miR-10b** | und | und | und | und | und | und | und | und |
| **miR-122** | und | 32.1941 | und | und | und | und | und | 34.7393 |
| **miR-124** | 27.9889 | 34.6418 | und | 34.0215 | 30.0550 | und | 30.1757 | 34.3530 |
| **miR-125a-3p** | und | 33.1883 | und | 31.6651 | und | 32.0759 | 31.8883 | 32.3221 |
| **miR-125a-5p** | 25.1319 | 24.0003 | 22.7256 | 23.3620 | 23.0888 | 22.9765 | 23.2714 | 22.9997 |
| **miR-125b** | 28.9885 | 27.4003 | 28.6908 | 31.2703 | 29.5724 | 27.4573 | 28.1604 | 28.1304 |
| **miR-126** | 20.6048 | 20.6163 | 20.5541 | 19.7602 | 20.2968 | 19.2545 | 20.0323 | 19.9149 |
| **miR-127-3p** | 25.8695 | 24.9707 | 27.7029 | 27.5549 | 23.0554 | 23.3610 | 24.2023 | 23.7034 |
| **miR-127-5p** | und | und | und | und | und | und | und | 35.1345 |
| **miR-128** | 30.2084 | 29.4950 | 29.6966 | 30.6040 | 31.9473 | 29.7120 | 31.3672 | 29.4900 |
| **miR-129-3p** | und | und | und | und | 26.8638 | 26.8384 | 26.7835 | 25.5293 |
| **miR-129-5p** | und | und | und | und | und | und | und | 34.6091 |
| **miR-130a** | 28.4686 | 29.1799 | 31.2494 | 31.8224 | 30.8556 | 29.1474 | 31.0866 | 30.0023 |
| **miR-130b** | 30.9251 | 28.3348 | 30.4585 | 32.3271 | 30.7544 | 28.9798 | 29.9099 | 28.1870 |
| **miR-132** | und | 28.3323 | 26.6266 | und | und | 26.8403 | 27.1248 | 26.3655 |
| **miR-133a** | 27.3136 | 26.1958 | 27.0417 | 26.7141 | 29.3408 | 28.3600 | 28.6558 | 27.8754 |
| **miR-133b** | und | 31.4249 | und | 32.6326 | und | 33.9566 | 36.2899 | 34.0102 |
| **miR-134** | und | 29.6212 | 30.1181 | 30.7169 | 29.7983 | 30.5851 | 30.8973 | 29.5538 |
| **miR-135a** | und | und | und | und | und | 37.1631 | und | und |
| **miR-135b** | und | und | und | und | und | 34.1753 | 33.1263 | 34.2961 |
| **miR-136** | und | und | und | und | 35.6910 | und | und | und |
| **miR-137** | und | und | und | und | und | und | und | und |
| **miR-138** | 27.0949 | 33.1816 | und | 30.8768 | 28.8517 | 29.4804 | 30.1531 | 28.7123 |
| **miR-139-3p** | und | und | und | und | und | und | und | und |
| **miR-139-5p** | und | und | und | 29.2090 | und | 27.9638 | 29.4527 | 29.1549 |
| **miR-140-3p** | und | 32.0969 | 31.9112 | 31.1497 | 33.4292 | 34.9936 | 33.6530 | 33.2986 |
| **miR-140-5p** | 26.7771 | 27.6030 | 27.9278 | 28.0230 | 28.8564 | 28.1613 | 28.4161 | 28.2151 |
| **miR-141** | und | und | und | 35.3763 | und | und | und | und |
| **miR-142-3p** | 22.9663 | 26.2513 | 26.7451 | 28.4061 | 25.6943 | 24.3901 | 26.5548 | 26.5463 |
| **miR-142-5p** | 29.1233 | 32.3284 | 31.8543 | 31.6152 | und | 31.6091 | 35.3718 | 32.9450 |
| **miR-143** | und | und | und | und | und | 35.4050 | und | und |
| **miR-145** | 29.0620 | 27.5592 | 28.5950 | 29.0717 | 27.6358 | 26.9366 | 27.3047 | 27.2382 |
| **miR-146a** | 22.0837 | 21.3809 | 21.8676 | 20.0464 | 23.2064 | 22.0255 | 22.0014 | 20.0993 |
| **miR-146b-3p** | 29.9341 | 29.4236 | 29.5889 | 30.1479 | 29.5122 | 27.9989 | 28.2272 | 29.1227 |
| **miR-146b-5p** | 23.6196 | 21.5391 | 21.4662 | 20.1629 | 22.4841 | 21.3754 | 21.5559 | 20.9656 |
| **miR-147** | und | und | und | und | und | und | und | und |
| **miR-147b** | und | und | und | und | und | und | und | und |
| **miR-148a** | und | 30.9995 | und | 31.9483 | 31.9860 | 28.9238 | 30.1879 | 29.2684 |
| **miR-148b** | und | 30.6065 | und | 33.7725 | und | 31.8788 | 30.9995 | 30.9724 |
| **miR-149** | und | 29.8194 | 29.6819 | 28.4839 | 25.6028 | 24.8843 | 25.1021 | 24.2150 |
| **miR-150** | 25.2104 | 23.7370 | 24.1753 | 24.1417 | 26.1866 | 25.1281 | 25.6756 | 25.3298 |
| **miR-152** | 30.0709 | 32.2363 | und | 30.0865 | und | 29.1139 | 30.4620 | 28.7464 |
| **miR-153** | und | und | und | und | und | und | und | und |
| **miR-154** | und | und | und | und | und | und | und | und |
| **miR-155** | und | und | und | und | und | und | und | und |
| **miR-15a** | und | und | und | und | und | und | und | und |
| **miR-15b** | 24.1301 | 23.3738 | 23.3542 | 24.8941 | 24.6274 | 23.0680 | 24.2177 | 23.0647 |
| **miR-16** | 24.0307 | 22.9188 | 23.4209 | 22.7481 | 24.2956 | 23.8338 | 24.0973 | 22.5415 |
| **miR-17** | 21.4461 | 20.4180 | 21.7070 | 20.9259 | 22.6492 | 22.2332 | 22.5859 | 20.9839 |
| **miR-181a** | 26.2550 | 26.5856 | 27.8091 | 29.4388 | 27.3506 | 25.6653 | 27.7645 | 27.1753 |
| **miR-181c** | und | 33.7767 | 33.1681 | 36.2956 | und | 32.9613 | 35.0602 | 34.1096 |
| **miR-182** | und | und | und | und | und | 34.3150 | 32.4820 | 30.6337 |
| **miR-183** | und | und | und | und | und | 32.8753 | 35.1669 | 32.7283 |
| **miR-184** | und | und | und | und | und | und | und | und |
| **miR-185** | und | und | 30.3918 | 30.3997 | und | und | und | 31.0636 |
| **miR-186** | 24.7758 | 24.1442 | 24.9139 | 24.1378 | 25.9572 | 25.6842 | 25.8095 | 25.4488 |
| **miR-187** | und | und | und | und | und | und | und | und |
| **miR-188-3p** | und | und | und | und | und | und | und | und |
| **miR-18a** | 27.2378 | 29.8335 | 30.2273 | 29.6495 | 28.8593 | 29.8799 | 30.9909 | 29.4012 |
| **miR-18b** | und | und | und | und | und | und | und | und |
| **miR-190** | und | und | und | und | und | und | und | und |
| **miR-191** | 23.5320 | 19.4117 | 18.9576 | 18.8153 | 20.0049 | 19.4561 | 18.9273 | 18.7370 |
| **miR-192** | 28.9817 | 31.0988 | 30.4540 | 32.2990 | 29.0735 | 29.4718 | 30.9986 | 29.7841 |
| **miR-193a-3p** | und | und | und | und | und | und | und | 38.0021 |
| **miR-193a-5p** | und | 30.1091 | 31.2838 | 32.3790 | 29.6471 | 28.7168 | 28.9606 | 28.3655 |
| **miR-193b** | 30.6635 | 28.0262 | 25.9873 | 24.9756 | 23.4347 | 22.1687 | 22.8403 | 21.9048 |
| **miR-194** | und | 33.8871 | 28.9023 | 30.9303 | 29.1293 | 33.0050 | 30.8239 | 32.0509 |
| **miR-195** | 29.0784 | 28.2102 | 28.0697 | 28.5004 | 29.4335 | 28.3015 | 29.4405 | 27.9101 |
| **miR-196b** | 26.1475 | 25.0170 | 24.7374 | 26.1939 | 28.8746 | 28.8005 | 28.5689 | 28.0329 |
| **miR-197** | 25.6341 | 23.2080 | 21.4220 | 22.7186 | 21.8498 | 22.4545 | 23.6778 | 23.3400 |
| **miR-198** | und | und | und | und | und | und | und | und |
| **miR-199a-3p** | 30.6007 | 28.5295 | 31.0321 | 29.0058 | 29.6630 | 28.5761 | 29.9696 | 28.9792 |
| **miR-199a-5p** | und | und | und | und | und | und | und | und |
| **miR-199b-5p** | und | und | und | und | und | 36.0401 | 33.9895 | 32.9489 |
| **miR-19a** | 25.6222 | 27.7098 | 26.5277 | 27.0015 | 27.5246 | 26.9428 | 28.0347 | 27.1329 |
| **miR-19b** | 22.2837 | 22.6531 | 22.1101 | 21.9813 | 22.7684 | 22.3344 | 23.4623 | 22.2564 |
| **miR-200a** | und | und | und | und | und | und | und | und |
| **miR-200b** | und | und | und | und | und | und | und | und |
| **miR-200c** | und | 29.0818 | 28.4884 | 28.3470 | 28.3835 | 29.1036 | 29.2461 | 29.0067 |
| **miR-202** | und | und | und | und | und | und | und | und |
| **miR-203** | 27.8163 | 30.2570 | 29.7903 | 30.9615 | 29.3601 | 30.8563 | 30.4294 | 29.5647 |
| **miR-204** | und | und | und | und | und | 31.5514 | 31.6009 | 30.0885 |
| **miR-205** | und | und | und | und | und | und | und | und |
| **miR-208** | und | und | und | und | und | und | und | und |
| **miR-208b** | und | und | und | und | und | und | und | und |
| **miR-20a** | 21.6781 | 23.1305 | 23.8432 | 24.0816 | 23.8875 | 23.1012 | 24.3072 | 23.4848 |
| **miR-20b** | 24.3225 | 23.7693 | 25.1161 | 24.8450 | 23.6007 | 25.1668 | 25.7056 | 24.7469 |
| **miR-21** | 25.8219 | 28.6854 | 27.8977 | 28.9879 | 27.4695 | 27.2468 | 28.7121 | 26.9829 |
| **miR-210** | und | und | und | 28.6434 | und | und | und | und |
| **miR-211** | und | und | und | und | und | 32.4501 | und | und |
| **miR-212** | und | und | 32.8944 | und | 30.0040 | und | 36.4749 | 31.8933 |
| **miR-214** | und | und | und | und | und | und | und | und |
| **miR-215** | und | und | und | 35.6263 | und | und | und | und |
| **miR-216a** | und | und | und | und | und | und | und | und |
| **miR-216b** | und | und | und | und | und | und | und | und |
| **miR-217** | und | und | und | und | und | und | und | und |
| **miR-218** | und | und | und | und | und | 33.5962 | und | 32.7050 |
| **miR-219-1-3p** | und | 33.4483 | und | und | 31.4818 | 35.2233 | 35.3486 | und |
| **miR-219-2-3p** | und | und | und | und | und | und | und | und |
| **miR-219-5p** | und | und | und | und | und | und | und | und |
| **miR-22** | und | und | und | und | und | und | und | und |
| **miR-220** | und | und | und | und | und | und | und | und |
| **miR-220b** | und | und | und | und | und | und | und | und |
| **miR-220c** | und | und | und | und | und | und | und | und |
| **miR-221** | 25.3130 | 24.2179 | 26.1965 | 26.2984 | und | 24.9909 | 24.7346 | 24.8271 |
| **miR-222** | 21.6231 | 19.8056 | 21.2473 | 21.2307 | 21.8143 | 20.7704 | 20.5591 | 20.2882 |
| **miR-223** | 19.2531 | 17.5776 | 19.1701 | 18.3738 | 20.6964 | 19.4812 | 19.3242 | 19.0584 |
| **miR-224** | und | und | und | und | und | 30.4025 | 30.4527 | 32.5496 |
| **miR-23a** | und | und | und | und | und | und | und | und |
| **miR-23b** | und | und | und | und | und | und | und | und |
| **miR-24** | 23.2505 | 22.3418 | 23.7420 | 22.6371 | 23.5506 | 23.2353 | 23.4726 | 21.9951 |
| **miR-25** | 25.9071 | 24.8934 | 26.7573 | 27.3870 | 25.2819 | 25.3393 | 26.1051 | 25.7830 |
| **miR-26a** | 26.1373 | 24.6227 | 25.8343 | 26.0056 | 25.7679 | 24.1727 | 25.3190 | 24.7025 |
| **miR-26b** | 28.6284 | 27.7725 | 28.7855 | 29.0422 | 29.2784 | 27.7576 | 28.5748 | 27.1914 |
| **miR-27a** | 27.7611 | 27.7033 | 28.3236 | 28.8383 | 27.8567 | 27.3467 | 28.6811 | 27.7368 |
| **miR-27b** | und | 30.7893 | 30.4924 | und | und | 30.2737 | 33.4301 | 35.3067 |
| **miR-28-3p** | 28.6695 | 26.2574 | 27.3891 | 25.8677 | 28.8002 | 27.4006 | 27.1649 | 26.1742 |
| **miR-28-5p** | 28.8087 | 29.5269 | 32.1606 | 30.5916 | und | 29.1269 | 29.9605 | 29.5520 |
| **miR-296-3p** | und | und | und | und | 31.3855 | und | und | und |
| **miR-296-5p** | 27.9459 | 28.8511 | 29.8488 | 30.3164 | 26.4820 | 26.9774 | 28.1971 | 27.0971 |
| **miR-298** | und | und | und | und | und | und | und | und |
| **miR-299-3p** | und | und | und | und | und | und | und | und |
| **miR-299-5p** | und | und | und | und | und | und | und | und |
| **miR-29a** | 25.5668 | 25.0157 | 25.9596 | 25.9994 | 26.0268 | 26.5095 | 27.0673 | 26.4128 |
| **miR-29b** | und | und | und | und | und | 35.4210 | und | und |
| **miR-29c** | 29.7338 | 31.5701 | 33.4978 | 32.5652 | 32.5668 | 30.3140 | 32.0153 | 32.1188 |
| **miR-301a** | 29.4754 | 30.8354 | 31.7506 | 32.1454 | 30.7103 | 30.0067 | 31.5332 | 30.6895 |
| **miR-301b** | und | 33.8882 | und | und | und | 33.6794 | 35.4748 | 31.4307 |
| **miR-302a** | und | und | und | und | und | und | und | und |
| **miR-302b** | und | und | und | und | und | und | und | und |
| **miR-302c** | und | und | und | und | und | und | und | und |
| **miR-30b** | 23.7093 | 22.2475 | 23.6127 | 23.4225 | 23.8388 | 22.5726 | 23.1806 | 22.4577 |
| **miR-30c** | 22.8652 | 21.3851 | 23.0921 | 22.4749 | 22.8003 | 21.9649 | 21.9737 | 21.6144 |
| **miR-31** | und | und | und | und | und | 31.7628 | 29.4346 | 28.6895 |
| **miR-32** | 29.6911 | und | und | 34.0390 | und | 35.2029 | und | 32.8420 |
| **miR-320** | 23.4745 | 20.8564 | 21.3223 | 21.0945 | 22.3231 | 22.1952 | 21.5035 | 21.1121 |
| **miR-323-3p** | und | 28.5793 | 29.5571 | 29.9658 | 27.7144 | 26.9665 | 27.4532 | 27.0741 |
| **miR-324-3p** | 28.1013 | 26.6657 | 27.9602 | 28.1406 | 27.7440 | 26.5642 | 27.7572 | 27.1881 |
| **miR-324-5p** | 29.7462 | 31.1746 | und | 32.2990 | und | 29.0964 | 31.8539 | 31.8274 |
| **miR-325** | und | und | und | und | und | und | und | und |
| **miR-326** | und | und | und | und | und | und | und | und |
| **miR-328** | 25.9207 | 24.9160 | 25.6404 | 26.3469 | 24.7274 | 23.6363 | 25.1079 | 24.6422 |
| **miR-329** | und | 39.2081 | und | und | und | 34.5006 | 38.9772 | und |
| **miR-330-3p** | und | 32.3137 | 31.2799 | 33.9749 | 31.7681 | 33.8352 | 32.2304 | 32.4056 |
| **miR-330-5p** | und | und | und | und | und | und | und | und |
| **miR-331-3p** | 25.1061 | 24.5009 | 23.4874 | 24.6665 | 25.3141 | 24.2106 | 24.8897 | 24.0791 |
| **miR-331-5p** | und | und | und | und | und | und | und | und |
| **miR-335** | und | 31.3778 | und | 32.3089 | 30.7137 | 30.2815 | 31.5366 | 28.8482 |
| **miR-337-5p** | und | und | und | und | und | und | und | und |
| **miR-338-3p** | und | und | und | und | und | und | und | und |
| **miR-339-3p** | 30.4651 | 28.2138 | 28.3828 | 28.1452 | 29.7005 | 29.7425 | 29.8242 | 28.6420 |
| **miR-339-5p** | und | und | und | und | und | und | und | und |
| **miR-33b** | und | und | und | und | und | und | und | und |
| **miR-340** | 30.6127 | 30.3555 | 31.1984 | 29.8249 | 29.6391 | 29.2191 | 29.9182 | 29.4164 |
| **miR-342-3p** | 24.3455 | 20.9435 | 20.1604 | 20.3368 | 22.1670 | 22.0609 | 21.6377 | 21.5156 |
| **miR-342-5p** | und | und | und | und | und | und | und | und |
| **miR-345** | 24.9375 | 24.7555 | 25.6143 | 25.1745 | 25.5873 | 26.0444 | 26.7146 | 25.9506 |
| **miR-346** | und | und | und | und | und | und | und | und |
| **miR-34a** | und | und | und | und | und | und | und | und |
| **miR-34c-5p** | und | und | und | und | und | und | und | und |
| **miR-361-5p** | 28.2738 | 28.8414 | und | 30.3123 | und | 33.4833 | 31.5470 | 30.2247 |
| **miR-362-3p** | und | und | und | und | 32.5848 | 33.8831 | 34.9860 | und |
| **miR-362-5p** | 31.4882 | 31.6622 | 32.7844 | 32.5666 | 30.9680 | 31.2958 | 30.4647 | 30.6003 |
| **miR-363** | und | 30.6657 | und | 34.1651 | und | 30.8862 | 31.3424 | 32.6246 |
| **miR-365** | 33.4149 | 29.3161 | 29.3231 | 30.1968 | 27.8216 | 26.7127 | 26.7831 | 26.1848 |
| **miR-367** | und | und | und | und | und | und | und | und |
| **miR-369-3p** | und | und | und | und | und | und | und | und |
| **miR-369-5p** | und | und | und | und | und | 35.8271 | und | 35.2645 |
| **miR-370** | und | 30.1959 | und | und | und | 27.0800 | 30.6233 | 28.6974 |
| **miR-371-3p** | 32.3322 | 32.1488 | und | 33.2105 | 29.3487 | 31.9082 | 33.5964 | 30.9470 |
| **miR-372** | und | und | und | und | und | und | und | und |
| **miR-373** | und | und | und | und | und | und | und | und |
| **miR-374a** | 26.7474 | 27.2185 | 27.4970 | 27.5755 | 27.6135 | 27.1905 | 28.0554 | 26.6376 |
| **miR-374b** | 26.9852 | 24.0216 | 25.5141 | 24.9657 | 28.0814 | 25.9640 | 25.9092 | 25.1772 |
| **miR-375** | und | 34.0128 | und | und | und | 32.2570 | 32.4508 | 31.7022 |
| **miR-376a** | und | 30.4788 | 31.0965 | 29.8093 | 30.6044 | 28.3725 | 30.6269 | 28.9765 |
| **miR-376b** | und | und | und | und | und | und | und | und |
| **miR-376c** | 32.5563 | 29.1620 | 29.4925 | 29.3951 | 28.6423 | 27.7636 | 28.5061 | 27.9793 |
| **miR-377** | und | und | und | und | und | und | und | und |
| **miR-379** | und | 32.4065 | und | 32.9566 | und | 29.4353 | 32.2323 | 30.3559 |
| **miR-380** | und | und | und | und | und | und | und | und |
| **miR-381** | und | und | und | und | und | und | und | und |
| **miR-382** | und | und | und | und | und | und | und | und |
| **miR-383** | und | und | und | und | und | und | und | und |
| **miR-384** | und | und | und | und | und | und | und | und |
| **miR-409-5p** | und | und | und | und | und | 35.2199 | und | und |
| **miR-410** | 30.3034 | 30.9967 | und | 32.2596 | und | 30.3648 | 30.5961 | 30.1132 |
| **miR-411** | und | 30.0142 | 32.0361 | 30.9777 | 28.8000 | 28.6901 | 29.9911 | 29.0765 |
| **miR-412** | und | und | und | und | und | und | und | und |
| **miR-422a** | 30.8586 | 30.4658 | 29.9147 | 32.5087 | und | 31.2503 | 32.0071 | 32.0322 |
| **miR-423-5p** | 31.5799 | 27.6665 | 28.1933 | 29.1378 | 28.1199 | 27.4538 | 28.3080 | 27.9469 |
| **miR-424** | und | und | und | und | und | 34.8511 | 35.7139 | 34.1282 |
| **miR-425** | und | 26.2883 | 26.7500 | 26.0929 | 26.4784 | 26.5844 | 26.0785 | 26.1497 |
| **miR-429** | und | und | und | und | und | und | und | und |
| **miR-431** | und | und | und | und | und | und | und | und |
| **miR-433** | 28.8137 | 27.8611 | 29.5708 | 28.6941 | 27.4379 | 26.3813 | 26.8754 | 26.3519 |
| **miR-448** | und | und | und | und | und | und | und | und |
| **miR-449a** | und | und | und | und | und | und | und | und |
| **miR-449b** | und | und | und | und | und | und | 34.4453 | und |
| **miR-450a** | und | und | und | und | 32.1087 | und | 35.0134 | 35.7736 |
| **miR-450b-3p** | und | und | und | und | und | und | und | und |
| **miR-450b-5p** | und | und | und | und | und | und | und | und |
| **miR-451** | und | und | und | und | und | und | und | und |
| **miR-452** | und | und | und | und | und | und | 33.1303 | 34.9340 |
| **miR-453** | und | und | und | und | und | und | und | und |
| **miR-454** | 26.9884 | 26.8271 | 26.6032 | 24.9594 | 27.4821 | 27.6332 | 27.5866 | 25.8264 |
| **miR-455-3p** | und | und | und | und | und | 31.2839 | 31.7828 | 31.4223 |
| **miR-455-5p** | und | und | und | und | und | und | und | 34.0988 |
| **miR-483-5p** | und | 32.7466 | und | 30.2509 | 26.8698 | 28.3977 | 27.9364 | 27.9739 |
| **miR-484** | 22.6792 | 20.7241 | 20.7582 | 20.6888 | 22.3850 | 21.2111 | 21.4118 | 21.0792 |
| **miR-485-3p** | und | 28.1206 | 29.3738 | 30.7767 | 28.2973 | 26.5769 | 27.5819 | 27.4503 |
| **miR-485-5p** | und | und | und | und | und | und | und | und |
| **miR-486-3p** | und | und | und | 32.9153 | und | und | und | und |
| **miR-486-5p** | und | 29.4268 | 27.6355 | 26.9565 | 29.3621 | 29.4794 | 30.2159 | 29.9573 |
| **miR-487a** | und | und | und | und | und | und | und | und |
| **miR-487b** | und | 30.1921 | und | 31.1699 | 31.0436 | 29.8931 | 30.0822 | 29.5344 |
| **miR-488** | und | und | und | und | und | und | und | und |
| **miR-489** | und | und | und | und | und | und | und | 34.1797 |
| **miR-490-3p** | und | und | und | und | und | und | und | und |
| **miR-491-3p** | und | und | und | und | und | und | und | und |
| **miR-491-5p** | 29.7449 | 28.4069 | 29.0368 | 29.5849 | 27.9306 | 28.0619 | 29.0777 | 29.1969 |
| **miR-492** | und | und | und | und | und | und | und | und |
| **miR-493** | und | 32.8008 | und | 34.7638 | und | 33.1777 | 32.3535 | 31.3993 |
| **miR-494** | 28.1362 | 28.6426 | 30.4261 | 28.0220 | 28.1582 | 29.7794 | 27.1811 | 29.0450 |
| **miR-495** | und | 28.8687 | und | 30.4003 | und | 29.5335 | 31.5106 | 29.0858 |
| **miR-496** | und | und | und | und | und | und | und | und |
| **miR-499-3p** | und | und | und | und | und | und | und | und |
| **miR-499-5p** | und | und | und | und | und | und | und | und |
| **miR-500** | und | 31.4960 | und | und | und | 34.5153 | 33.1905 | 30.7240 |
| **miR-501-3p** | und | und | und | und | und | und | und | und |
| **miR-501-5p** | und | und | und | und | und | und | und | und |
| **miR-502-3p** | 31.8147 | 34.6663 | und | 34.1574 | 33.2604 | 32.9735 | 34.5801 | 32.9646 |
| **miR-502-5p** | und | und | und | und | und | und | 35.4274 | 33.5425 |
| **miR-503** | und | 33.6433 | und | und | und | und | und | und |
| **miR-504** | und | und | und | und | und | und | und | und |
| **miR-505** | und | und | und | und | und | und | und | und |
| **miR-506** | und | 35.1217 | und | und | und | und | und | und |
| **miR-507** | und | und | und | und | und | und | und | und |
| **miR-508-3p** | und | 34.7576 | und | und | und | und | und | und |
| **miR-508-5p** | und | und | und | und | und | und | und | und |
| **miR-509-3-5p** | und | und | und | und | und | und | und | und |
| **miR-509-5p** | und | und | und | 31.8295 | und | und | und | und |
| **miR-510** | und | und | und | 34.7869 | und | und | und | und |
| **miR-511** | und | 31.9380 | und | 32.9037 | und | und | und | und |
| **miR-512-3p** | und | und | und | und | und | und | und | und |
| **miR-512-5p** | und | und | und | und | und | und | und | und |
| **miR-513-5p** | und | und | und | und | und | und | und | und |
| **miR-515-3p** | und | und | und | und | und | und | und | und |
| **miR-515-5p** | und | und | und | und | und | und | und | und |
| **miR-516a-5p** | und | und | und | und | und | und | und | und |
| **miR-516b** | und | und | und | und | und | und | und | und |
| **miR-517a** | und | und | und | 34.5530 | und | 35.0613 | 35.1485 | und |
| **miR-517b** | und | und | und | und | und | und | und | und |
| **miR-517c** | und | 35.3123 | und | und | und | und | 33.5043 | 35.0652 |
| **miR-518a-3p** | und | und | und | und | und | und | und | und |
| **miR-518a-5p** | und | und | und | und | und | und | und | und |
| **miR-518b** | 32.1569 | 36.4698 | und | und | und | und | und | und |
| **miR-518c** | und | und | und | und | und | und | und | und |
| **miR-518d-3p** | und | und | 31.3670 | und | und | und | und | und |
| **miR-518d-5p** | und | und | und | und | und | und | und | und |
| **miR-518e** | und | und | und | und | und | 35.7530 | 33.0867 | 33.2358 |
| **miR-518f** | und | und | und | und | und | und | 35.3672 | und |
| **miR-519a** | und | 37.7314 | und | 34.8797 | und | und | 35.2410 | 34.6867 |
| **miR-519c-3p** | und | und | und | und | und | und | und | und |
| **miR-519d** | und | und | und | und | und | und | 34.7373 | 34.9423 |
| **miR-519e** | und | und | und | und | und | und | und | und |
| **miR-520a-3p** | und | und | und | und | und | und | und | und |
| **miR-520a-5p** | und | und | und | und | und | und | und | und |
| **miR-520b** | 32.8760 | und | und | 36.8399 | und | 38.0141 | und | 38.5503 |
| **miR-520d-5p** | 29.5233 | 31.0512 | 28.4904 | 31.3125 | 28.0871 | 31.1640 | 31.5118 | 31.4958 |
| **miR-520e** | und | und | und | und | und | und | und | und |
| **miR-520f** | und | und | und | und | und | und | und | und |
| **miR-520g** | und | und | und | und | und | und | und | und |
| **miR-521** | und | und | und | und | und | und | und | und |
| **miR-522** | und | und | und | und | und | und | 36.2422 | und |
| **miR-523** | und | und | und | und | und | und | und | und |
| **miR-524-5p** | und | und | und | und | und | und | und | und |
| **miR-525-3p** | und | und | und | und | und | und | und | und |
| **miR-525-5p** | und | und | und | und | und | und | und | und |
| **miR-526b** | und | und | und | und | und | und | und | und |
| **miR-532-3p** | 28.0598 | 26.4441 | 26.5959 | 27.2887 | 25.9605 | 24.2704 | 25.1337 | 24.5023 |
| **miR-532-5p** | und | 28.7080 | und | 29.3557 | 30.3769 | 27.5571 | 27.9736 | 27.2690 |
| **miR-539** | und | 28.5512 | 30.1161 | 30.9517 | 29.6758 | 28.3612 | 27.9010 | 27.3291 |
| **miR-541** | und | und | und | und | und | und | 34.7351 | und |
| **miR-542-3p** | und | und | und | und | und | und | und | und |
| **miR-542-5p** | und | und | und | und | und | und | und | und |
| **miR-544** | und | und | und | und | und | und | und | und |
| **miR-545** | und | 32.2515 | und | und | und | 34.4995 | und | und |
| **miR-548a-3p** | 30.3280 | 38.6404 | 32.5538 | 34.5975 | und | 34.4221 | 34.7910 | 36.4207 |
| **miR-548a-5p** | und | und | und | und | und | und | und | und |
| **miR-548b-3p** | und | und | und | und | und | und | und | und |
| **miR-548b-5p** | und | und | und | und | und | und | und | und |
| **miR-548c-3p** | 32.4405 | und | 34.5969 | und | und | 33.9656 | und | 35.1311 |
| **miR-548c-5p** | und | und | und | und | und | 37.2116 | und | und |
| **miR-548d-3p** | und | und | und | und | und | und | und | und |
| **miR-548d-5p** | und | und | und | und | und | und | und | und |
| **miR-551b** | und | und | und | und | und | und | und | und |
| **miR-556-3p** | und | und | und | und | und | und | und | und |
| **miR-556-5p** | und | und | und | und | und | und | und | und |
| **miR-561** | und | und | und | und | und | und | und | und |
| **miR-570** | und | und | und | und | und | und | und | und |
| **miR-574-3p** | 26.7951 | 24.2231 | 22.7800 | 22.5451 | 24.2194 | 24.6319 | 23.7615 | 23.4945 |
| **miR-576-3p** | und | und | und | und | und | und | und | 35.1888 |
| **miR-576-5p** | und | und | und | und | und | und | 35.9251 | und |
| **miR-579** | und | und | und | und | und | und | 34.8862 | und |
| **miR-582-3p** | und | und | und | und | und | und | und | und |
| **miR-582-5p** | und | und | und | und | und | und | und | und |
| **miR-589** | und | und | und | und | und | 35.0205 | 34.1924 | und |
| **miR-590-5p** | 29.5623 | 30.5370 | 30.3012 | 29.5950 | 31.5171 | 29.0008 | 31.8040 | 31.4424 |
| **miR-597** | 30.7061 | 33.0628 | und | und | und | und | 34.1255 | 33.9865 |
| **miR-598** | und | 30.6089 | 30.4576 | 35.0238 | 30.2778 | 29.6827 | 31.2238 | 29.9668 |
| **miR-615-3p** | und | und | und | und | und | und | und | und |
| **miR-615-5p** | und | und | und | und | und | und | und | und |
| **miR-616** | und | und | und | und | und | und | und | und |
| **miR-618** | und | und | und | und | 34.4914 | und | und | und |
| **miR-624** | und | und | und | und | und | und | und | und |
| **miR-625** | 31.5699 | 30.4704 | 30.6816 | 31.9076 | 30.7736 | 30.8584 | 31.1900 | 31.1266 |
| **miR-627** | und | und | und | und | und | und | und | und |
| **miR-628-5p** | 31.4665 | 31.0932 | 30.7075 | 30.5196 | und | 33.1861 | 31.8733 | 30.7975 |
| **miR-629** | und | 31.7046 | und | und | 31.7270 | 34.8194 | 35.2065 | 31.9878 |
| **miR-636** | und | 30.2343 | 30.7844 | 29.9468 | und | 31.0639 | 32.9144 | 31.0464 |
| **miR-642** | und | 30.1174 | 29.6769 | 30.0599 | 27.9430 | 25.5676 | 26.9070 | 26.5032 |
| **miR-651** | und | und | und | und | und | und | und | und |
| **miR-652** | 28.4123 | 29.8424 | 31.1947 | 30.3696 | 29.5137 | 27.9846 | 28.2799 | 28.5824 |
| **miR-653** | und | und | und | und | und | und | und | und |
| **miR-654-3p** | und | und | und | und | 30.0623 | 31.8795 | 32.2687 | 32.9998 |
| **miR-654-5p** | und | und | und | und | und | und | und | und |
| **miR-655** | 34.7124 | 35.0613 | und | 39.5978 | 33.3509 | 33.7203 | 34.8529 | 32.5299 |
| **miR-660** | und | und | und | und | und | 29.9970 | 30.8657 | 31.1982 |
| **miR-671-3p** | 28.8305 | 26.4653 | 27.7963 | 27.0708 | 27.7462 | 26.6895 | 27.0881 | 26.7658 |
| **miR-672** | und | und | und | und | und | und | und | und |
| **miR-674** | und | und | und | und | und | und | und | und |
| **miR-708** | und | und | und | und | und | und | und | und |
| **miR-744** | 28.0869 | 25.9706 | 28.3129 | 27.1355 | 27.1876 | 26.3050 | 26.9737 | 25.8290 |
| **miR-758** | und | 30.9992 | und | und | und | 31.3651 | 33.3487 | 31.6404 |
| **miR-871** | und | und | und | und | und | und | und | und |
| **miR-872** | und | und | und | und | und | und | und | und |
| **miR-873** | und | und | und | und | und | und | und | und |
| **miR-874** | und | und | und | und | und | und | und | und |
| **miR-875-3p** | und | und | und | und | und | und | und | und |
| **miR-876-3p** | und | und | und | und | und | und | 34.3533 | und |
| **miR-876-5p** | und | und | und | und | und | und | und | und |
| **miR-885-3p** | und | und | und | und | und | und | und | und |
| **miR-885-5p** | 30.2472 | 31.2708 | 28.1852 | 29.1131 | 29.5789 | 28.7758 | 29.0725 | 30.5008 |
| **miR-886-3p** | 27.8923 | 28.6189 | 28.1911 | 26.4665 | 24.5064 | 27.0556 | 24.9074 | 25.5677 |
| **miR-886-5p** | 27.7750 | 26.5154 | 25.3699 | 26.1354 | 25.6895 | 26.8170 | 25.9189 | 26.3073 |
| **miR-887** | und | und | und | und | und | und | und | und |
| **miR-888** | und | und | und | und | und | und | und | und |
| **miR-889** | und | 31.6344 | und | und | und | 31.9685 | 35.7179 | 35.1514 |
| **miR-890** | und | und | und | und | und | und | und | und |
| **miR-891a** | und | und | und | und | und | und | und | und |
| **miR-891b** | und | und | und | und | und | und | und | und |
| **miR-892a** | und | und | und | und | und | und | und | und |
| **miR-9** | und | und | und | und | 29.2465 | 28.3646 | 29.7990 | 28.2162 |
| **miR-92a** | 20.8165 | 19.8324 | 20.7384 | 21.3137 | 21.5453 | 20.3519 | 20.9641 | 20.6228 |
| **miR-93** | 24.1335 | 23.5056 | 25.8236 | 25.5340 | 26.6272 | 25.0425 | 25.6021 | 24.4081 |
| **miR-95** | und | und | und | und | und | 36.4693 | und | 32.6038 |
| **miR-96** | und | und | und | und | und | und | und | und |
| **miR-98** | und | und | und | und | und | und | und | und |
| **miR-99a** | und | 28.4838 | und | 30.7890 | 30.4526 | 28.7986 | 30.2763 | 29.4643 |
| **miR-99b** | 26.1318 | 26.2126 | 26.3617 | 27.7541 | 24.7073 | 25.2798 | 25.7145 | 25.4546 |
| **RNU44** | und | 19.9727 | 20.6655 | 21.0437 | 20.2798 | 18.9022 | 19.0875 | 19.4674 |
| **RNU48** | 18.7492 | 16.6621 | 16.2328 | 15.6586 | 17.0367 | 16.2357 | 16.2542 | 15.5028 |
