# Supplementary material for: Histone Deacetylase Inhibition Enhances Self Renewal and Cardioprotection by Human Cord Blood-Derived CD34+ Cells
Source: PLoS One. 2011 Jul 18;6(7):e22158. doi: 10.1371/journal.pone.0022158 (PMC3138768; doi:10.1371/journal.pone.0022158)
Supplement: Table S5 — Ct raw data of TaqMan Human MicroRNA Arrays Card B. ath-miR159a: negative control. (DOCX) [file pone.0022158.s015.docx]

**Table S5**

| **Name** | **C64** | **C68** | **C70** | **C71** | **V64** | **V68** | **V70** | **V71** |
| --- | --- | --- | --- | --- | --- | --- | --- | --- |
| **ath-miR159a** | und | und | und | und | und | und | und | und |
| **let-7a*** | und | und | und | und | und | und | und | und |
| **let-7b*** | und | und | und | und | und | und | und | und |
| **let-7d*** | und | und | und | und | und | und | und | und |
| **let-7e*** | 35.9799 | 35.9838 | und | und | und | 35.9755 | 34.9656 | und |
| **let-7f-1*** | und | und | und | und | und | und | und | und |
| **let-7f-2*** | und | und | und | und | und | und | und | und |
| **let-7g*** | und | und | und | und | und | und | und | und |
| **let-7i*** | und | und | und | 35.0184 | und | und | und | und |
| **MammU6** | 23.9010 | 16.9643 | 18.5187 | 13.6052 | 16.9103 | 17.4946 | 16.9256 | 13.7009 |
| **MammU6** | 22.7654 | 16.6948 | 18.9776 | 13.9892 | 16.9783 | 18.0005 | 17.6617 | 13.8007 |
| **MammU6** | 22.9574 | 16.8649 | 18.4113 | 13.8230 | 16.4873 | 17.3687 | 16.9467 | 13.8043 |
| **MammU6** | 23.4719 | 16.7080 | 18.5660 | 13.4258 | 16.5080 | 17.4377 | 16.8370 | 13.9654 |
| **miR-100*** | und | und | und | und | und | und | und | und |
| **miR-101*** | und | und | und | und | und | und | und | und |
| **miR-105*** | und | und | und | und | und | und | und | und |
| **miR-106a*** | und | und | und | und | und | und | und | und |
| **miR-106b*** | und | und | und | und | und | und | und | und |
| **miR-10a*** | und | und | und | und | und | 33.9221 | und | und |
| **miR-10b*** | und | 33.9456 | und | und | und | und | und | und |
| **miR-122*** | und | und | und | und | und | und | und | und |
| **miR-124*** | und | und | und | und | und | und | und | und |
| **miR-125b-1*** | und | und | und | und | und | und | und | und |
| **miR-125b-2*** | und | und | und | und | und | und | und | und |
| **miR-126*** | 28.9626 | 26.9744 | 29.9816 | 25.9573 | 28.9805 | 23.9684 | 25.9746 | 24.9679 |
| **miR-130a*** | und | und | und | und | und | und | und | und |
| **miR-130b*** | 32.9514 | 27.0291 | 28.9464 | 26.9715 | 29.9998 | 25.9877 | 26.9783 | 26.9823 |
| **miR-132*** | und | und | und | und | und | und | und | und |
| **miR-135a*** | 24.9685 | 21.9376 | 25.9830 | 23.9533 | 23.9793 | 20.9648 | 21.9558 | 21.9560 |
| **miR-135b*** | und | und | und | und | und | und | und | und |
| **miR-136*** | und | 32.9643 | und | und | 36.0571 | 31.9684 | 35.9739 | 33.9807 |
| **miR-138-1*** | 25.9692 | 25.9663 | 27.9762 | 26.9845 | 25.9757 | 25.9606 | 25.9617 | 25.9833 |
| **miR-138-2*** | und | und | und | und | und | und | und | und |
| **miR-141*** | und | und | und | und | und | und | und | und |
| **miR-143*** | und | und | und | und | und | und | und | und |
| **miR-144*** | und | und | und | 33.9872 | und | 30.9825 | 32.9802 | 32.0718 |
| **miR-145*** | und | 35.9759 | und | und | und | und | und | und |
| **miR-146a*** | und | und | und | und | und | und | und | und |
| **miR-148a*** | und | und | und | und | und | und | und | und |
| **miR-148b*** | und | und | und | 35.9864 | und | 35.9872 | und | 36.9886 |
| **miR-149*** | und | und | und | und | und | 26.9676 | und | und |
| **miR-151-3p** | 35.9004 | 30.9868 | 31.9755 | 29.9746 | 32.9785 | 30.9744 | 30.9746 | 29.9640 |
| **miR-154*** | und | und | und | und | und | und | und | und |
| **miR-155*** | und | 35.9748 | und | und | und | und | und | und |
| **miR-15a*** | und | und | und | und | und | 32.9858 | 36.0196 | und |
| **miR-15b*** | und | und | und | und | und | und | und | und |
| **miR-16-1*** | und | und | und | und | und | 31.9939 | und | und |
| **miR-16-2*** | und | und | und | und | und | und | und | und |
| **miR-17*** | und | 36.0255 | und | und | und | 33.9688 | und | und |
| **miR-181a*** | 31.9726 | 28.9914 | 32.9846 | 31.9800 | 32.9910 | 28.9853 | 29.9784 | 30.0036 |
| **miR-181a-2*** | und | 27.9909 | 30.9265 | 29.9603 | 31.9702 | 29.9872 | 31.0165 | 29.9624 |
| **miR-181c*** | und | und | und | und | und | und | und | und |
| **miR-182*** | und | und | und | und | und | und | und | und |
| **miR-183*** | und | und | und | und | und | und | 30.9533 | und |
| **miR-185*** | und | und | und | und | und | und | und | und |
| **miR-186*** | und | und | und | und | und | und | und | und |
| **miR-188-5p** | und | 28.9784 | 27.0153 | 25.9549 | 27.0600 | 27.9871 | und | und |
| **miR-18a*** | 35.9868 | 29.9709 | 31.9962 | 30.9450 | 33.9912 | 30.9680 | 31.9706 | 30.9996 |
| **miR-18b*** | und | und | und | und | und | und | und | und |
| **miR-190b** | und | 33.9829 | und | und | und | und | 35.9850 | und |
| **miR-192*** | und | und | und | und | und | 34.9715 | und | 35.9632 |
| **miR-193b*** | und | und | und | und | 31.0620 | 28.9879 | 29.9938 | 28.9649 |
| **miR-194*** | und | und | und | und | und | und | und | und |
| **miR-195*** | und | und | und | und | und | und | und | und |
| **miR-19a*** | und | und | und | und | und | und | und | und |
| **miR-19b-1*** | 32.9898 | 30.9736 | 35.9839 | 31.9809 | 33.9720 | 31.9884 | 31.9635 | 31.9643 |
| **miR-19b-2*** | und | und | und | und | und | und | und | und |
| **miR-200a*** | und | und | und | und | und | und | und | und |
| **miR-200b*** | und | und | und | und | und | und | und | und |
| **miR-200c*** | und | und | und | und | und | und | und | und |
| **miR-202*** | und | und | und | und | und | und | und | und |
| **miR-206** | und | und | und | und | und | 34.0097 | und | 35.9698 |
| **miR-20a*** | 35.9993 | 32.9853 | 33.9669 | 32.9831 | 33.9784 | 33.9898 | 34.9927 | 35.9825 |
| **miR-20b*** | und | und | und | und | und | 36.0242 | 35.9638 | und |
| **miR-21*** | und | und | und | 36.0435 | und | und | und | und |
| **miR-214*** | und | und | und | und | und | und | und | und |
| **miR-218-2*** | und | und | und | und | und | und | und | und |
| **miR-22*** | und | 33.9151 | und | und | und | 31.9573 | 32.0049 | und |
| **miR-221*** | und | und | und | und | und | und | und | und |
| **miR-222*** | und | 28.9898 | und | 30.0086 | und | 28.9714 | 28.9593 | 28.9856 |
| **miR-223*** | 32.9853 | 28.9826 | 31.9889 | 28.9644 | 31.9644 | 29.9807 | 29.9793 | 29.9910 |
| **miR-23a*** | 34.9781 | und | und | und | und | und | und | und |
| **miR-23b*** | und | und | und | und | und | und | und | und |
| **miR-24-1*** | und | und | und | und | und | und | und | und |
| **miR-25*** | und | 30.9368 | und | und | und | und | und | und |
| **miR-26a-1*** | und | 32.0005 | 33.9655 | 31.9815 | 35.9831 | 31.9967 | 31.9981 | 31.9683 |
| **miR-26a-2*** | und | 35.9794 | und | und | und | und | und | 35.9839 |
| **miR-26b*** | 33.9873 | 29.9801 | 35.0054 | 30.9772 | 32.9742 | 29.9713 | 30.9658 | 31.9830 |
| **miR-27a*** | 29.9816 | 27.9773 | 30.9649 | 28.9961 | 30.9725 | 29.9841 | 27.9568 | 28.9700 |
| **miR-27b*** | und | und | und | und | und | und | 32.0128 | und |
| **miR-29a*** | und | und | und | und | und | und | und | und |
| **miR-29b-1*** | und | und | und | und | und | und | und | und |
| **miR-29b-2*** | und | und | und | 35.9864 | und | 33.9842 | 35.9577 | und |
| **miR-29c*** | 35.9928 | 34.9787 | und | und | und | 32.9659 | 33.9831 | 33.9726 |
| **miR-302a*** | und | und | und | und | und | und | und | und |
| **miR-302b*** | und | und | und | und | und | und | und | und |
| **miR-302c*** | und | und | und | und | und | und | und | und |
| **miR-302d** | und | und | und | und | und | und | und | und |
| **miR-302d** | und | und | und | und | und | und | und | und |
| **miR-302d*** | und | und | und | und | und | und | und | und |
| **miR-30a** | 30.9665 | 27.9699 | 32.9716 | 30.9647 | 31.9685 | 26.9603 | 28.9913 | 28.9716 |
| **miR-30a*** | 31.9608 | 30.9923 | 33.0189 | 32.0169 | 29.9914 | 26.9733 | 27.9852 | 26.9803 |
| **miR-30b*** | und | und | und | und | und | und | und | und |
| **miR-30c-1*** | und | und | und | und | und | und | und | und |
| **miR-30c-2*** | und | und | und | und | und | und | und | und |
| **miR-30d** | 31.3627 | 28.1776 | 35.9912 | 32.6500 | 33.9584 | 27.5666 | 28.9980 | 30.9813 |
| **miR-30d** | 33.0158 | 27.9765 | und | 31.9402 | 33.6840 | 27.9640 | 29.3819 | 30.9259 |
| **miR-30d*** | und | 31.9804 | und | 33.9977 | und | 32.0141 | 31.9764 | 32.0376 |
| **miR-30e** | 30.9638 | 29.9681 | 30.9913 | 29.9711 | 29.9576 | 28.9584 | 30.0132 | 30.0222 |
| **miR-30e*** | 32.0006 | 27.9528 | 31.9803 | 30.9812 | 29.9699 | 27.9761 | 27.9675 | 26.9613 |
| **miR-32*** | und | und | und | und | und | und | und | und |
| **miR-335*** | und | 28.0076 | und | 28.9940 | und | 28.0194 | 29.0054 | 28.9819 |
| **miR-337-3p** | und | 31.0190 | und | und | und | 30.9511 | und | und |
| **miR-33a*** | und | und | und | und | und | und | und | und |
| **miR-340*** | 32.9906 | 28.9748 | 34.9983 | 30.9713 | 32.9534 | 28.9695 | 29.9694 | 28.9850 |
| **miR-34a*** | und | 32.9902 | und | 34.0036 | und | 33.9834 | und | 34.0178 |
| **miR-34b*** | und | und | und | und | und | und | und | und |
| **miR-361-3p** | und | und | und | und | und | und | und | und |
| **miR-361-3p** | und | und | und | und | und | und | und | und |
| **miR-363*** | und | 32.9721 | 34.9877 | und | und | und | 32.9606 | und |
| **miR-367*** | und | und | und | und | und | und | und | und |
| **miR-373*** | und | und | und | und | und | und | und | und |
| **miR-374a*** | und | und | und | und | und | und | und | und |
| **miR-374b*** | und | und | und | und | und | und | und | und |
| **miR-376a*** | und | und | und | und | und | und | und | und |
| **miR-377*** | und | und | und | und | und | und | und | und |
| **miR-378** | 34.0295 | 30.9823 | 32.9705 | 32.9796 | 34.0100 | 30.9650 | 31.9847 | 31.9767 |
| **miR-378*** | und | 32.9871 | und | 35.9710 | und | 33.9935 | 36.0156 | 36.0432 |
| **miR-379*** | und | und | und | 34.0052 | und | und | und | und |
| **miR-380*** | und | und | und | und | und | 36.0013 | und | und |
| **miR-409-3p** | und | 26.9920 | 34.6914 | 30.0005 | 35.1568 | 25.9997 | und | 25.9590 |
| **miR-409-3p** | und | 26.7400 | 33.0461 | 29.7901 | 34.0212 | 25.6671 | 25.9982 | 25.9727 |
| **miR-411*** | und | 34.0032 | und | 32.9845 | und | und | und | 33.0185 |
| **miR-424*** | und | und | 36.0096 | und | und | und | und | und |
| **miR-425*** | 33.9925 | 27.9675 | 30.9711 | 27.9518 | 32.9844 | 29.9977 | 28.9497 | 28.9693 |
| **miR-431*** | und | und | und | und | und | und | und | und |
| **miR-432** | und | 27.9833 | 29.9776 | 27.9740 | 29.4183 | 26.7915 | 25.9341 | 25.8705 |
| **miR-432** | und | 27.8603 | 30.9829 | 27.5395 | 29.9941 | 26.9705 | 25.9698 | 25.9748 |
| **miR-432*** | und | und | und | und | und | und | und | und |
| **miR-452*** | und | und | und | und | und | und | und | und |
| **miR-454*** | und | 30.9834 | 32.9896 | 30.9954 | 35.9688 | 31.9913 | 31.9760 | 30.9764 |
| **miR-488*** | und | und | und | und | und | und | und | und |
| **miR-493*** | und | 32.9807 | und | und | und | 32.9867 | 33.9912 | 34.0091 |
| **miR-497** | und | und | und | und | und | und | und | und |
| **miR-497** | und | und | und | und | und | und | und | 33.0723 |
| **miR-497*** | und | und | und | und | und | und | und | und |
| **miR-498** | und | und | und | und | und | und | und | und |
| **miR-500*** | und | und | und | und | und | und | und | und |
| **miR-505*** | und | 30.9832 | 31.9565 | 31.9785 | 33.9954 | 30.9850 | 30.9794 | 30.9868 |
| **miR-509-3p** | 29.9719 | 28.9775 | 29.9709 | 27.9615 | 30.9680 | 30.0051 | 30.9568 | 29.9755 |
| **miR-513-3p** | und | und | und | und | und | und | und | und |
| **miR-516a-3p** | und | 35.9614 | und | und | 35.9892 | 34.9735 | 35.9746 | und |
| **miR-517*** | und | und | und | und | und | und | und | und |
| **miR-518c*** | und | und | und | und | und | und | und | und |
| **miR-518e*** | und | und | und | und | und | und | und | und |
| **miR-518f*** | und | und | und | und | und | und | und | und |
| **miR-519b-3p** | und | und | und | und | und | und | und | und |
| **miR-519b-3p** | und | und | und | 35.9917 | und | und | und | und |
| **miR-519e*** | und | und | und | und | und | und | und | und |
| **miR-520c-3p** | und | und | und | und | und | und | und | und |
| **miR-520h** | und | und | und | und | und | und | und | und |
| **miR-524-3p** | und | und | und | und | und | und | und | und |
| **miR-524-3p** | und | und | und | und | und | und | und | und |
| **miR-526b*** | 34.9772 | 36.0320 | 35.9760 | 33.9787 | 35.8773 | 36.0180 | 34.9827 | 34.9821 |
| **miR-541*** | und | und | und | und | und | und | und | und |
| **miR-543** | und | 34.0146 | und | und | und | und | 30.9814 | 32.0098 |
| **miR-545*** | und | und | und | und | und | und | und | und |
| **miR-549** | und | und | und | und | und | und | und | und |
| **miR-550** | und | und | 36.0076 | 35.9791 | und | 35.9962 | 35.9920 | 36.0049 |
| **miR-550*** | und | und | und | 34.0213 | und | und | und | 32.9892 |
| **miR-551a** | und | und | und | und | und | und | und | und |
| **miR-551b*** | und | und | 35.9810 | 35.9950 | 34.9849 | 32.9703 | 32.9862 | 33.9909 |
| **miR-552** | und | und | und | und | und | und | und | und |
| **miR-553** | und | und | und | und | und | und | und | und |
| **miR-554** | und | und | und | und | und | und | und | und |
| **miR-555** | und | und | und | und | und | und | und | und |
| **miR-557** | und | und | und | und | und | und | und | und |
| **miR-558** | und | und | und | und | und | und | und | und |
| **miR-559** | und | und | und | und | und | und | und | und |
| **miR-559** | und | und | und | und | und | und | und | und |
| **miR-562** | und | und | und | und | und | und | und | und |
| **miR-562** | und | und | und | und | und | und | und | und |
| **miR-563** | und | und | und | und | und | und | und | und |
| **miR-564** | und | und | und | 33.9932 | und | und | und | und |
| **miR-565** | 30.9919 | 28.9869 | und | 29.9737 | 28.9819 | 27.9327 | 28.9577 | 27.9659 |
| **miR-566** | und | und | und | und | und | und | und | und |
| **miR-566** | und | und | und | und | und | und | und | und |
| **miR-567** | und | und | und | und | und | und | und | und |
| **miR-569** | und | und | und | und | und | und | und | und |
| **miR-571** | und | und | und | und | und | und | und | und |
| **miR-571** | und | und | und | und | und | und | und | und |
| **miR-572** | und | und | und | und | und | und | 33.0565 | 32.9769 |
| **miR-572** | und | und | und | und | und | und | und | und |
| **miR-573** | und | und | 32.9927 | und | und | und | und | und |
| **miR-575** | und | und | und | und | und | und | und | und |
| **miR-578** | und | und | und | und | und | und | und | und |
| **miR-578** | und | und | und | und | und | und | und | und |
| **miR-580** | und | und | und | und | und | und | und | und |
| **miR-580** | und | und | und | und | und | und | und | und |
| **miR-581** | und | und | und | und | und | und | und | und |
| **miR-581** | und | und | und | und | und | und | und | und |
| **miR-583** | und | und | und | und | und | und | und | und |
| **miR-584** | und | und | und | und | und | und | und | und |
| **miR-584** | und | und | und | und | und | und | und | und |
| **miR-585** | und | und | und | und | und | und | und | und |
| **miR-586** | und | und | und | und | und | und | und | und |
| **miR-587** | und | und | und | und | und | und | und | und |
| **miR-588** | und | und | und | und | und | und | und | und |
| **miR-588** | und | und | und | und | und | und | und | und |
| **miR-589*** | und | 32.0026 | und | 32.0413 | und | und | 31.0077 | 30.0726 |
| **miR-591** | und | und | und | und | und | und | und | und |
| **miR-591** | und | und | und | und | und | und | und | und |
| **miR-592** | und | und | und | und | und | und | und | und |
| **miR-593** | und | und | und | und | und | und | und | und |
| **miR-593*** | und | und | und | und | und | und | und | und |
| **miR-595** | und | und | und | und | und | und | und | und |
| **miR-596** | und | und | und | und | und | und | und | und |
| **miR-596** | und | und | und | und | und | und | und | und |
| **miR-599** | und | und | und | und | und | und | und | und |
| **miR-599** | und | und | und | und | und | und | und | und |
| **miR-600** | und | und | und | und | und | und | und | und |
| **miR-600** | und | und | und | und | und | und | und | und |
| **miR-601** | 35.9424 | 35.9627 | und | 35.9470 | und | 33.9519 | 33.9542 | 33.9546 |
| **miR-603** | und | und | und | und | und | und | und | und |
| **miR-604** | und | und | und | und | und | und | und | und |
| **miR-604** | und | und | und | und | und | und | und | und |
| **miR-605** | und | und | und | und | und | und | und | und |
| **miR-605** | und | und | und | und | und | und | und | und |
| **miR-606** | und | und | und | und | und | und | und | und |
| **miR-606** | und | und | und | und | und | und | und | und |
| **miR-607** | und | und | und | und | und | und | und | und |
| **miR-607** | und | und | und | und | und | und | und | und |
| **miR-608** | und | und | und | und | und | und | und | und |
| **miR-608** | und | und | und | und | und | und | und | und |
| **miR-609** | und | und | und | und | und | und | und | und |
| **miR-609** | und | und | und | und | und | und | und | und |
| **miR-610** | und | 29.9518 | und | und | 30.0101 | 29.9541 | und | und |
| **miR-612** | und | und | und | und | und | und | und | und |
| **miR-613** | und | und | und | und | und | und | und | und |
| **miR-613** | und | und | und | und | und | und | und | und |
| **miR-614** | und | und | und | und | und | und | und | und |
| **miR-614** | und | und | und | und | und | und | und | und |
| **miR-616*** | und | und | 34.9832 | 35.9897 | und | und | 33.9864 | 32.9899 |
| **miR-617** | und | und | und | und | und | und | und | und |
| **miR-619** | und | und | und | und | und | und | und | und |
| **miR-621** | und | und | und | und | und | und | und | und |
| **miR-621** | und | und | und | und | und | und | und | und |
| **miR-622** | und | und | und | und | und | und | und | und |
| **miR-622** | und | und | und | und | und | und | und | und |
| **miR-623** | und | und | und | und | 36.0346 | und | und | und |
| **miR-623** | und | und | und | 36.9719 | und | und | und | und |
| **miR-624*** | und | und | und | und | und | und | und | und |
| **miR-625*** | 32.9906 | 25.9471 | 27.9790 | 23.9496 | 28.9709 | 26.9861 | 25.9701 | 25.9810 |
| **miR-626** | und | und | und | und | und | und | und | und |
| **miR-626** | und | und | und | und | und | und | und | und |
| **miR-628-3p** | und | 32.6657 | und | 30.9120 | und | 34.1093 | 32.5212 | 31.9376 |
| **miR-628-3p** | und | 32.9585 | und | 30.9787 | 34.9992 | und | 34.0150 | 33.0353 |
| **miR-629*** | 33.0122 | 27.9453 | 28.9578 | 27.9943 | 30.9899 | 27.9566 | 27.9816 | 27.9704 |
| **miR-630** | und | und | und | und | und | und | und | und |
| **miR-631** | und | und | und | und | und | und | und | und |
| **miR-631** | und | und | und | und | und | und | und | und |
| **miR-632** | 31.0012 | 30.9830 | 31.9498 | 32.9792 | 30.9781 | 30.9954 | 29.9914 | 31.0020 |
| **miR-633** | und | und | und | und | und | und | und | und |
| **miR-633** | und | und | und | und | und | und | und | und |
| **miR-634** | und | und | und | und | und | und | und | und |
| **miR-634** | und | und | und | und | und | und | und | und |
| **miR-635** | und | und | und | und | und | und | und | und |
| **miR-635** | und | und | und | und | und | und | und | und |
| **miR-637** | und | und | und | und | und | und | und | und |
| **miR-637** | und | und | und | und | und | und | und | und |
| **miR-638** | und | und | und | und | und | und | und | und |
| **miR-639** | und | und | und | und | und | und | 31.0006 | 30.9665 |
| **miR-639** | und | und | und | und | und | und | und | und |
| **miR-640** | und | und | und | und | und | und | und | und |
| **miR-640** | und | und | und | und | und | und | und | und |
| **miR-641** | und | und | und | und | und | und | und | und |
| **miR-641** | und | und | und | und | und | und | und | und |
| **miR-643** | und | und | und | und | und | und | und | und |
| **miR-644** | und | und | und | und | und | und | und | und |
| **miR-644** | und | und | und | und | und | und | und | und |
| **miR-645** | 33.9893 | und | und | und | und | 34.1835 | 35.6843 | und |
| **miR-645** | 34.9185 | und | und | und | und | 33.9795 | 36.9843 | und |
| **miR-646** | und | und | und | und | und | und | und | und |
| **miR-646** | und | und | und | und | und | und | und | und |
| **miR-647** | und | und | und | und | und | und | und | und |
| **miR-647** | und | und | und | und | und | und | und | und |
| **miR-648** | und | und | und | und | und | und | und | und |
| **miR-648** | und | und | und | und | und | und | und | und |
| **miR-649** | und | und | und | und | und | und | und | und |
| **miR-649** | und | und | und | und | und | und | und | und |
| **miR-650** | und | und | und | und | und | und | und | und |
| **miR-650** | und | und | und | und | und | und | und | und |
| **miR-656** | und | und | und | und | und | und | und | 32.0256 |
| **miR-656** | und | und | und | und | und | und | und | 31.4185 |
| **miR-657** | und | und | und | und | und | und | und | und |
| **miR-657** | und | und | und | und | und | und | und | und |
| **miR-658** | und | und | und | und | und | und | und | und |
| **miR-658** | und | und | und | und | und | und | und | und |
| **miR-659** | und | und | und | und | und | und | und | und |
| **miR-661** | und | und | und | und | und | und | und | und |
| **miR-662** | und | und | und | und | und | und | und | und |
| **miR-662** | und | und | und | und | und | und | und | und |
| **miR-668** | und | und | und | und | und | und | und | und |
| **miR-668** | und | und | und | und | und | und | und | und |
| **miR-675** | und | und | und | und | und | und | und | und |
| **miR-675** | und | und | und | und | und | und | und | und |
| **miR-7** | und | und | und | und | und | und | und | und |
| **miR-7** | und | und | und | und | und | und | und | und |
| **miR-708*** | und | und | und | und | und | und | und | und |
| **miR-7-1*** | und | 28.9735 | und | 29.0143 | und | 33.9833 | und | 29.0185 |
| **miR-7-2*** | und | und | und | und | und | und | und | und |
| **miR-744*** | und | und | und | und | und | und | und | und |
| **miR-760** | und | und | und | und | und | 26.9185 | und | und |
| **miR-766** | 28.9750 | 23.9590 | 25.9738 | 23.9829 | 26.9766 | 23.9643 | 23.9754 | 23.9957 |
| **miR-766** | 28.8773 | 23.8405 | 25.6520 | 23.8609 | 26.9776 | 23.8675 | 23.8591 | 23.7385 |
| **miR-767-3p** | und | und | und | und | und | und | und | und |
| **miR-767-3p** | und | und | und | und | und | und | und | und |
| **miR-767-5p** | und | und | und | und | und | und | und | und |
| **miR-767-5p** | und | und | und | und | und | und | und | und |
| **miR-768-3p** | 30.9691 | 24.8121 | 28.9147 | 26.8385 | 26.9516 | 23.9625 | 23.9505 | 23.8552 |
| **miR-768-3p** | 31.4858 | 24.9512 | 28.9676 | 26.9634 | 26.8613 | 23.9517 | 23.7591 | 23.9607 |
| **miR-769-3p** | und | und | und | und | und | und | und | und |
| **miR-769-3p** | und | und | und | und | und | und | und | und |
| **miR-769-5p** | und | 29.7611 | und | 34.0041 | 32.6449 | 30.0449 | 30.2277 | 30.9850 |
| **miR-769-5p** | und | 30.0013 | und | 32.0382 | 32.9850 | 30.9309 | 30.9905 | 30.6401 |
| **miR-770-5p** | und | und | und | und | und | und | und | und |
| **miR-801** | 28.0022 | 26.9974 | 36.0178 | 22.9901 | 25.9981 | 29.9917 | 22.9372 | 23.9817 |
| **miR-801** | 28.1454 | 27.0403 | und | 22.9524 | 25.9873 | 29.4609 | 22.9794 | 23.7822 |
| **miR-875-5p** | und | und | und | und | und | und | und | und |
| **miR-877** | 30.9949 | 27.9620 | 28.9741 | 29.0079 | 29.9828 | 28.9863 | 28.9886 | 29.0086 |
| **miR-888*** | und | und | und | und | und | und | und | und |
| **miR-892b** | und | und | und | und | und | und | und | und |
| **miR-9*** | und | und | und | und | und | 31.9676 | 33.9642 | 31.9768 |
| **miR-920** | und | und | und | und | und | und | und | und |
| **miR-920** | und | und | und | und | und | und | und | und |
| **miR-921** | und | und | und | und | und | und | und | und |
| **miR-921** | und | und | und | und | und | und | und | und |
| **miR-922** | und | und | und | und | und | und | 35.8867 | 35.9864 |
| **miR-922** | und | und | und | und | und | und | 35.8884 | 34.5489 |
| **miR-923** | 23.9568 | 22.8776 | 25.3525 | 20.0516 | und | 22.9816 | 20.9849 | 19.9877 |
| **miR-923** | und | 23.0479 | 24.9932 | 20.0054 | 21.0192 | 22.4786 | 20.7753 | 20.0035 |
| **miR-924** | und | und | und | und | und | und | und | und |
| **miR-924** | und | und | und | und | und | und | und | und |
| **miR-92a-1*** | 35.9582 | 28.9574 | 31.9446 | 30.9549 | 32.9558 | 29.9772 | 30.9679 | 29.9647 |
| **miR-92a-2*** | und | 35.9889 | und | und | und | und | und | und |
| **miR-92b*** | und | und | und | und | und | und | und | und |
| **miR-93*** | 31.9765 | 27.9565 | 30.9711 | 27.9680 | 31.9862 | 29.9689 | 28.9665 | 28.9651 |
| **miR-933** | und | und | und | und | und | und | und | und |
| **miR-933** | und | und | und | und | und | und | und | und |
| **miR-934** | und | und | und | und | und | und | und | und |
| **miR-934** | und | und | und | und | und | und | und | und |
| **miR-935** | und | und | und | und | 26.9197 | 23.9764 | 23.9685 | 24.0177 |
| **miR-935** | und | und | und | und | 27.0141 | 24.0259 | 23.8617 | 23.9913 |
| **miR-936** | und | und | und | und | und | und | und | und |
| **miR-937** | und | 33.9894 | und | 33.9808 | und | 33.9759 | 33.9713 | 32.9878 |
| **miR-938** | und | und | und | und | und | und | und | und |
| **miR-939** | und | und | und | und | und | und | und | und |
| **miR-941** | und | 30.9573 | 33.9878 | 31.9643 | und | 30.0239 | 30.9969 | 30.9587 |
| **miR-942** | 35.9755 | 28.9927 | 32.0108 | 30.0059 | 36.0036 | 29.9710 | 30.9460 | 30.0032 |
| **miR-943** | und | und | und | und | und | und | und | und |
| **miR-944** | und | und | und | und | und | und | und | und |
| **miR-96*** | und | und | und | und | und | und | und | und |
| **miR-99a*** | und | und | und | und | und | und | und | und |
| **miR-99b*** | und | 30.9843 | 34.0127 | 29.9895 | und | 31.0166 | 30.9660 | 30.9680 |
| **RNU24** | 31.7555 | 21.9591 | 24.9391 | 22.9453 | 24.9675 | 21.8937 | 21.9756 | 20.9449 |
| **RNU24** | 31.5788 | 21.8661 | 24.9688 | 22.8406 | 24.9725 | 21.9576 | 21.9565 | 20.6737 |
| **RNU24** | 31.3262 | 21.7453 | 24.9114 | 22.8669 | 24.9513 | 21.9258 | 21.9549 | 20.6197 |
| **RNU24** | 30.9503 | 21.9104 | 24.7875 | 22.6962 | 24.9374 | 21.9670 | 21.9854 | 20.7940 |
| **RNU43** | und | und | und | und | und | und | und | und |
| **RNU43** | und | und | und | und | und | und | und | und |
| **RNU43** | und | und | und | und | und | und | und | und |
| **RNU43** | und | und | und | und | und | und | und | und |
| **RNU44** | und | 19.6850 | 22.5618 | 20.5844 | 21.6459 | 18.8982 | 19.6492 | 18.9550 |
| **RNU44** | und | 19.7405 | 22.6412 | 20.9429 | 21.9327 | 18.9587 | 19.9432 | 18.9540 |
| **RNU44** | und | 19.6065 | 22.3120 | 20.7672 | 21.4799 | 18.6153 | 19.8569 | 18.3878 |
| **RNU44** | und | 19.9173 | 22.9589 | 20.7094 | 21.4366 | 18.6098 | 18.8992 | 18.7860 |
| **RNU48** | 22.9515 | 17.7896 | 19.7338 | 16.6764 | 19.8883 | 16.6812 | 16.8637 | 16.9111 |
| **RNU48** | 23.0299 | 17.9832 | 19.9807 | 16.9700 | 19.9886 | 16.9656 | 16.9849 | 16.9764 |
| **RNU48** | 22.9350 | 17.7143 | 19.6948 | 16.7337 | 19.8277 | 16.5549 | 16.9450 | 16.9174 |
| **RNU48** | 22.8805 | 17.6269 | 19.7372 | 16.6722 | 19.7228 | 16.6273 | 16.8010 | 16.7180 |
| **RNU6B** | und | 27.9832 | 29.8615 | 28.4952 | 28.9119 | 26.9428 | 27.5561 | 26.8904 |
| **RNU6B** | und | 27.5658 | 29.4150 | 28.8634 | 28.9373 | 26.9182 | 27.9410 | 26.5720 |
| **RNU6B** | und | 27.4724 | 30.1982 | 28.3998 | 28.1386 | 26.6303 | 27.6052 | 26.4659 |
| **RNU6B** | und | 27.5347 | 29.9416 | 28.4086 | 28.5887 | 26.7437 | 27.7780 | 26.8795 |
