# Supplementary material for: Histone Deacetylase Inhibition Enhances Self Renewal and Cardioprotection by Human Cord Blood-Derived CD34+ Cells
Source: PLoS One. 2011 Jul 18;6(7):e22158. doi: 10.1371/journal.pone.0022158 (PMC3138768; doi:10.1371/journal.pone.0022158)
Supplement: Materials and Methods S1 — Detailed description of experimental procedures. (DOCX) [file pone.0022158.s016.docx]

**Supplementary Materials and Methods**

Cord blood collection, selection and expansion of CD34^+^ cells

Mononuclear cells (MNCs) were separated by Ficoll density gradient centrifugation and CD34^+^ cells were selected by immunomagnetic separation using direct MiniMACS (Miltenyi Biotec) CD34 isolation kit according to the manufacturer’s instruction. Cells (1x10^5^/well) were seeded into 96-well plates and expanded in Stem Span (StemCell Technologies Inc.) containing a cytokine mixture with IL-3 (Biodesign) and IL-6 (Endogen; both at 20 ng/ml), Flt3-Ligand (FLT3L; Meridian LifeScience) and Stem cell factor (SCF; Thermo Scientific; both at 100 ng/ml). Cells were incubated at 37° C and 5% CO_2_; culture medium was changed every second day with fresh complete medium. In VPA and TSA (both from Sigma Aldrich, Italy) cultures the drug was added at each medium change. To assess HDACi effect on cell expansion we performed a set of experiments comparing VPA (1mM, 2.5mM and 5mM) and TSA (5ng/ml, 12.5ng/ml and 25ng/ml) to control (CTR) condition. After 3 and 5 days of expansion we evaluated cell number, viability and immunophenotype. Using VPA 2.5mM cells were plated for 7, 14 and 21 days to evaluate the drug effect over a longer period.

Immunophenotype, cell growth, stem cell activity analysis by flow cytometry

The antibodies used to perform immunophenotype of control and HDACi-treated CD34^+^ cells were as follows: CD29-PECy5, CD31-FITC, CD34-PE, CD45-PE, CD49b-FITC, CD73-PE, CD90-PE, CD146-PE (all from BD Pharmingen, Italy), CD14-PE, CD133-PE (from Miltenyi Biotec), KDR-PE, CD105-PE (from R&D Systems, USA), CD144-PE (from eBioscience, UK), CD49d-FITC and CD49e-FITC (from Beckman Coulter). For dioctadecyl-tetramethylindo-carbocyanine perchlorate (DiI)-labeling, DiIAcLDL (Biomedical Technologies, 2 µg/ml) uptake assay was performed by incubating cells for 4 hours at 37°C. After staining procedures, cells were immediately analyzed using a FACSCalibur cytometer (Becton Dickinson) and then analyzed. Data plotting was performed using FACSDiva software (Becton Dickinson).

CFSE staining was performed using the CFSE Cell Proliferation kit (Invitrogen) according to manufacturer instructions. Briefly cell (4x10^6^/ml concentration) were stained with 1μM CFSE for 10 minutes in the dark at RT. The reaction was stopped by adding 1/5 of the volume of FBS and 10 volumes of complete medium. After 5 minutes, cells were washed and seeded. Due to CFSE bright fluorescence intensity immediately after staining day 0 FACS analyses were performed 12 hours after labelling. To detect CD34 expression in CFSE stained cells, co-staining with CD34-PE was performed as previously described. The analyses were repeated after 5 and 7 days of expansion. Data were analyzed with software CellQuest and ModFit; they were plotted with FACSDiva.

Cell cycle analysis of CTR and VPA-treated cells was performed by using a conventional propidium Iodide stainin procedure. Cells (2x10^5^) cells were washed and resuspended in 500 μL of PI/RNase Staining Buffer (Becton Dickinson). After 20 minutes samples were transferred into FACS tubes and immediately analyzed using FACScalibur flow cytometer. Data were finally analyzed by ModFit software. To discern the different distribution of CD34^+^cells between G0 and G1 phases, a co-staining with a Ki67-specific antibody and CD34-PE antibody was performed. Staining for kKi67 was performed as follows: cells were first incubated with with CD34 PE antibody and then fixed in 2% PFA for 10 minutes at room temperature. After washing they were permeabilized with Perm/Wash solution (staining buffer, Becton Dickinson) for 10^min^. Cells were resuspended in 100 μl of staining buffer at concentration of 1x10^6^ cells/ml and finally stained with 10 μl of anti-human Ki67 FITC (Beckton Dickinson) for 1h at RT. After a washing in staining buffer and before analysis in a FACScalibur flow cytometer (Beckton Dickinson), cells were suspended in 300 μl PBS.

To assess the activity of *MDR1* gene product, cells (1x10^6^cells/ml) were incubated with Rhodamine123 (Sigma-Aldrich), at a 0.2 μg/ml for 2 hours at 4°C in the dark in complete culture medium. After incubation, cells were washed and resuspended in fresh complete medium. To detect specific extrusion of the fluorescent dye, one half of the cells in each replicate was incubated with the MDR1 Pgp channel inhibitor Verapamil (150 μM; Sigma Aldrich Italy). All samples were then incubated at 37°C in the dark for 2^h^15^min^. Before analysis in FACSCalibur flow cytometer, cells incubated with Rho123 ± Verapamil were finally co-stained with isotype-APC or anti CD34-APC (Becton Dickinson) antibodies for 40 minutes in ice to avoid extrusion of the Rho123 dye.

For ALDH activity detection, the Aldefluor staining kit was used (Stem Cell Technologies). Briefly, up to 1x10^6^ cells were re-suspended in 1 ml Aldefluor Staining Buffer and incubated with 5 μL activated Aldefluor reagent. Half of the sample volume was transferred in a tube containing 2.5 μL the reaction inhibitor, DEAB. All samples were incubated for 1 hour at 37°C in the dark and then stained with anti CD34-APC or the relative isotype control as described for Rho123.

Culture of Endothelial Colony-Forming Cells (ECFCs)

To obtain formation of ECFC clusters the method described in [1] was followed. After 7 days of culture in CTR and VPA conditions, CD34^+^ cells were washed and resuspended in complete EGM-2 medium (Lonza) containing 10% Fetal Bovine Serum (FBS, Euroclone) and 1% antibiotics (Penicillin-Streptomycin, Lonza). Cells were seeded in a 6-well tissue culture plate pre-coated with type 1 rat tail collagen (BD Biosciences) at a 2.5x10^5^cells/ml density. After two days, adherent cells were washed with complete medium. Colonies appeared after further 13-20 days of culture. Cells were then expanded in T75 flasks pre-coated with type 1 rat tail collagen (BD Biosciences). After two passages in culture (P2), cells were collected, washed and stained with the following antibodies: CD34-PE, CD31-FITC, CD45-PE, CD146-PE (from BD Biosciences), CD133-PE (Miltenyi Biotech) and KDR-PE (R&D Systems). Corresponding isotype staining was used as a negative control. After staining, cells were immediately analyzed using a FACSCalibur cytometer (Becton Dickinson). Data plotting was performed using Cell Quest software (Becton Dickinson). The formation of capillary-like structures by ECFCs in vitro was performed using Cultrex Basement Membrane Extract (Cultrex BME, R&D Systems) in 96-well culture plates. To this aim, 2x10^4^ P2 cells/well were seeded in 250μl of complete medium. The same amount of HUVEC cells were used as positive control. The formation of capillary-like structures was observed after 16 hours culture at 37°C.

qRT-PCR for small Cyclin/CDK inhibitors detection

Total RNA was purified from day 7 CTR and VPA-treated cells using Trizol protocol (Invitrogen, CA). For each sample, 1 μg of total RNA was treated with DNase I and it was reverse transcribed (RT) using SuperScriptIII cDNA synthesis kit (both from Invitrogen, CA).

Real time q-RT-PCR analysis was performed using iQ5 Real Time PCR System (Bio-Rad, Italy). Each primer pair was tested in duplicate using 5ng of the cDNA. Primers were designed from available human sequences by using the primer analysis software Primer Express v3.0 (Applied Biosystems, CA). The PCR-reaction included 5ng of template cDNA, 0.2μM of each (forward and reverse) primers, and 12.5 μl of iQ SYBR Green Supermix (Bio-Rad, Italy), conjugated with the fluorescent dye FAM in a total volume of 25 μl. Cycling conditions were as follows: 95°C enzyme activation for 10 min, followed by 40 cycles of amplification (95°C 15’’ denaturation, 60°C 1 min annealing/elongation). Quantified values were normalized against the input determined by the housekeeping human gene β-actin. Data are expressed and plotted as relative expression. The primer sequences are:

**p14 ^ARF^**  Fw GTTTTCGTGGTTCACACT and Rv CCTCAGTAGCATCAGCAC

**p16 ^INK4^** Fw ATGGAGCCTTCGGCTGACT and Rv CGTAACTATTCGGTGCGTTG

**p21^Cip1/Waf1^**  Fw CAGCATGACAGATTTCTACCACTCC and Rv ATGTAGAGCGGGCCTTTGAG

**p27** Fw ACCTGCAACCGACGATTCTTC and Rv GGGCGTCTGCTCCACAGA

Chromatin Immunoprecipitation

Formaldehyde cross-linking of exponentially growing cells and immunoprecipitation of chromatin (ChIP) were performed using ChIP-IT Express Enzymatic kit according manufacture’s instruction (Active Motif). DNA fragments were recovered and analyzed by quantitative PCR (qPCR) as previously described[2]. Briefly, standard curves were generated by serial dilution of the input (5-log dilutions in triplicate) and the data, normalized to corresponding DNA input control, are represented as relative enrichment. The following primers pairs to amplify specific human *CD34* gene promoter for qPCR were as follow (from distal to proximal region relative to the TSS, as indicated in the Figure 4D).

**687Fw** 5’-TTCGCCCTTGCTCCTTGTAC-3’ and **756Rev** 5’-CATGCACACTGAGGTTCAAGAAG-3’

**1502Fw** 5’-GAGTGTCTGCGGAGAAATCAGAA-3’ and **1599Rev** 5’-CAGGCTCTTCCATGATGTCTGTAC-3’

**2390Fw** 5’-GCAAGGCTGCCACAAAGG-3’ and **2520Rev** 5’-GCCGTCGAGGCCAAAAA-3’

**3055Fw** 5’-GCGTTAGCCGAGGTGTGAA-3’ and **3113Rev** 5’-CGCTTTATCTCAGTCCATTGGAA-3’

Transcript and miRNA profiling

For RT^2^ Profiler PCR Arrays, total RNA from cells cultured for 7 days (n=4, Control and VPA-treated) was isolated using TRIzol (Invitrogen). RNA was cleaned up using RNeasy Mini Kit (Qiagen). RNA quality was evaluated by capillary electrophoresis (Experion System, Bio-Rad Laboratories). Before reverse transcription (RT), 150 ng RNA/sample was subject to a second genomic DNA elimination step. Quantitative PCR (qPCR) cocktails were loaded using provided 384EZLoad Covers (SABiosciences) and performed using into 7900HT Fast Real-Time PCR System (Applied Biosystems).

MicroRNAs profiling was performed in CD34^bright^ cells. Cell sorting was performed by incubation of control and VPA-treated cells with isotype-APC and CD34-APC antibody followed by sorting into a FACSAria flow cytometer (Beckton Dickinson). A purity checking was performed after cell sorting to control the purity of isolated cells before mRNA extraction. Total RNA from high throughput-sorted cells (n=4, n=4, Control and VPA-treated cells) using TRIzol protocol (Invitrogen). RNA quality was evaluated by capillary electrophoresis using Experion System (Bio-Rad Laboratories). TaqMan Human MicroRNA A and B Arrays, version 2.0 (Applied Biosystems, USA), were used for the contemporary detection of 733 human miRNAs. According to the manufacturer’s protocol, (RT) reaction were performed using 98.4 ng total RNA from each sample, while in pre-amplification steps, 2.5 µl of each RT product were used. All steps were performed using a 7900HT Fast Real-Time PCR System (Applied Biosystems).

Analysis of stem cells-related transcripts

RT^2^ Profiler PCR Arrays were analyzed using the software ABI Prism SDS version 2.3. Optimal baseline was determined automatically by the software algorithm for each amplification curve and threshold for Ct (cycle threshold) was set manually at the same value in all plates, in order to obtain closely similar Ct for RT and PCR positive controls across the different plates. This allowed comparison of multiple arrays. All Ct values reported as greater than 35 or as not detected were changed to 35 and considered a negative call. Three sets of replicate control wells (genomic DNA, RT, and positive PCR controls) were used to assess each groups' level of genomic DNA contamination, reverse transcription efficiency, and PCR reproducibility, as well as to test for inter-well and intra-plate consistency.

Raw expression intensities of target mRNAs were normalized for differences in the amount of total RNA added to each reaction using a set of five reference genes (β-actin, ACTB; β-2-microglobulin, B2M; glyceraldehyde-3-phosphate dehydrogenase, GAPDH; hypoxanthine guanine phosphoribosyl transferase 1, HPRT1; and ribosomal protein L13a, RPL13A). Analysis of gene expression stability and selection of the best reference genes were performed using the NormFinder version 0.953 Excel Add-In[3].

Relative quantitation of gene expression was performed using the comparative Ct method (∆Ct). ∆Ct value was defined as the difference between the Ct of a gene (either target or housekeeping) in the calibrator sample (the sample with the highest expression, i.e. lowest Ct value) and the Ct of the same gene in experimental sample. The Ct values were transformed to raw, not-normalized quantities using the formula η^ΔCt^, where amplification efficiency (η) was set arbitrarily to 2 (100%). Gene expression normalization factors were obtained calculating the geometric mean of the raw quantities of the three most stable reference genes (ACTB, GAPDH, and RPL13A). The normalized expression of each target mRNA was finally calculated by dividing the raw gene-of-interest quantity by the appropriate normalization factor. Finally, the mean fold change of VPA-treated CD34^+^ *vs.* control cells was calculated, by dividing the normalized mean expression value in CD34^+^ cells by the mean expression value in control cells.

The MultiExperiment Viewer (MeV) software version 4.6[4] was used for high-level analysis. Genes were deemed as non informative and filtered out from all plates when the percent of negative calls exceeded 50%. Unsupervised hierarchical cluster analysis was performed to assess the similarity and differences in gene expression profiles among the samples and whether expression profiles discriminates treatment groups (Figure 6A). The similarity of gene expression among arrays and probes was assessed by calculating Pearson’s correlation coefficient using an algorithm first described by Eisen *et al* [5]. Normalized gene expression values were log transformed (log base 2), mean centered by gene mean and clustered by correlation (centered) average linkage, using leaf order optimization. Differentially expressed genes among treatment groups were identified using a multivariate paired *t*-test, using Welch approximation (assuming unequal group variances), computing p-values based on 100 permutations with a confidence level of 80% (1-α), and a limiting the false discovery rate (FDR) proportion to <0.1. Differences in gene expression were considered statistically significant if their p-value was less than 0.05. Differentially expressed genes were then clustered using the same distance metric and linkage method, in order to visualize and confirm the existence of the gene signature.

Gene enrichment analysis

Gene functional classification was performed using the web-based application Database for Annotation, Visualization and Integrated Discovery (DAVID 6.7, <http://david.abcc.ncifcrf.gov>)[6]. DAVID functional annotation clustering and chart tools were used to query Biocarta and KEGG pathway databases, in order to identify significantly non-redundant over-represented biological themes in the differentially expressed gene set. Gene-enrichment analysis was performed using the EASE algorithm, which compares the representation of functional classes for genes within differentially regulated subset to the entirety represented on the array. EASE performs a conservative adjustment of the Fisher’s exact test to calculate the probability that the composition of the differentially expressed set occurs by chance. A pathway was considered differentially regulated if the significance level was < 0.05 (see Table 3).

Analysis of miRNA profiles

Low level analysis and quality control of TaqMan Human MicroRNA A and B Arrays were performed with the software ABI Prism SDS version 2.3. All Ct values reported as greater than 35 or as not detected were changed to 35 and considered a negative call.

Raw expression intensities of target miRNAs were normalized for differences in the amount of total RNA added to each reaction using the mean expression value of all expressed miRNAs in a given sample, following the method recently described by Mestdagh *et al* [7]. Relative quantitation of miRNA expression was performed using the comparative Ct method (∆Ct) described above. The mean fold change of VPA-treated CD34^+^ *vs.* control cells was calculated as well.

The BRB-ArrayTools software package version 3.81, developed by Dr. Richard Simon and BRB-ArrayTools Development Team, was used for high-level statistical analysis and the MeV 4.6 software for clustering analysis. Normalized gene expression values were log_2_ transformed. Genes were deemed as non informative and filtered out from all plates when the percentile of negative calls exceeded 50 and when the percentile of the log-ratio variation in less than 10. Unsupervised hierarchical cluster analysis was performed to assess whether expression profiles discriminates treatment groups (Figure 6B). The distance metric used for calculating the similarity of gene expression among arrays and probes was the Spearman rank correlation. Log_2_ transformed gene expression values were mean centered by gene mean; gene and sample vectors were normalized and clustered by average linkage, using leaf order optimization. Differentially expressed genes among treatment groups were identified using a random-variance model for univariate significance paired *t*-test. The random-variance *t*-test is an improvement over the standard separate *t*-test as it permits sharing information among genes about within-class variation without assuming that all genes have the same variance[8]. A univariate permutation test was also performed to confirm the statistical significance of the findings, based on all available permutations. The maximum proportion of FDR was <0.2. Differences in gene expression were considered statistically significant if their p-value was less than 0.05. Differentially expressed genes were then clustered using Pearson’s correlation (centered) and average linkage method.

Analysis of cell secretome

Unsupervised hierarchical clustering and statistical analysis of the two bead-based multiplex immunoassay data were performed using the MeV 4.6 software. Log_2_ transformed protein concentrations were centered by mean or median values, and clustered using Pearson’s correlation (centered) and average linkage method, with leaf order optimization. Differentially expressed proteins among treatment groups were identified using a paired *t*-test, controlling for the FDR proportion (<0.1). Differences in gene expression were considered statistically significant if their p-value was less than 0.05.

Analysis of apoptosis in HL-1 cells exposed to hypoxia

After 7 days of culture in CTR and VPA conditions, CTR and VPA treated cells (10^5^ cells/well) were seeded into a 96-well culture plate in Dulbecco's Modified Eagle Medium (DMEM, Lonza) without citokines. After 48 hours, conditioned media (CM) were collected. HL1 cardiac cell line derived from the AT-1 mouse atrial myocyte tumour strain [9], were incubated at 37°C in 5%CO_2_ with CM or DMEM for two hours before the hypoxic treatment. In separate experimental groups, cells were no treated (normoxia control: 95% air and 5% CO_2_) or were exposed to hypoxia in an atmosphere containing 5% CO_2_ and 95% N_2_ into a hermetically closed chamber for 16 h.

Apoptosis immunoassay

At the end of the incubation period, quantification of histone-complexed DNA fragments (mono- and oligonucleosomes) in HL-1 cells was performed by one-step sandwich immunoassay (Roche Diagnostics, Mannheim Germany) followed by normalization to total cell proteins determined by Bradford assay. Data are expressed as percentage of apoptosis in CTR *vs.* VPA cells under hypoxia conditions.

Animal model

All procedures were conducted in conformity with the institutional guidelines that are in compliance with national and international laws and policies. 130 SCID Beige male mice with an initial body weight of 20-23 were anesthetized with isoflurane 1.5% and ventilated with a mechanical ventilator (Ugo Basile 28026 mouse ventilator tidal volume 0.2 mL; 120 strokes/min) through an endotracheal cannula. The left anterior descending coronary artery was ligated with a 7-O silk (Ethicon) suture after exteriorization of the heart through a 15-mm opening at the fourth intercostal space. 15 minutes after ligation, PBS, CTR or VPA cells were injected in the left ventricular (LV) wall in the infarct border zone (1.5x10^5^ cells/2.5 μl). The chest was then closed under negative pressure and mice were weaned from mechanical ventilation. Post surgical analgesia was achieved by buprenorphine (0.1mg/kg s.c. q12h for 1 day).

Echocardiography

Four weeks after CAL, transthoracic ultra-imaging echocardiography (VisualSonics, Vevo 770) was performed on anesthetized mice (isoflurane: 0.5-1.5% in O_2_). Animals were positioned on a rail system for maintaining body temperature and scan head position under ECG and respiration monitoring.

A parasternal long-axis B-mode image was acquired with appropriate positioning of the probe to obtain the maximum left ventricular (LV) length, necessary for LV volumes measurements and calculations. Short-axis B-mode images of the LV were recorded from the base to the apex by translating the mouse platform by 1 mm increments along the LV long-axis. M-Mode tracings were recorded at the mid papillary level with two-dimensional image guidance. All images were stored in DICOM format to be processed off-line. LV measurements and calculations were performed as recommended by the American and European Societies of Ecocardiography. LV internal diastolic and systolic diameters (LVIDd and LVIDs), LV wall thicknesses (septal, antero-septal and posterior), and fractional shortening (FS) were evaluated at the mid papillary level from the M-Mode modality in both PLAX and PSAX views; LV volumes (LVEDV and LVESV) were measured and calculated from the PLAX view with the modified Simpson method (ComPACS software, Medimatic S.R.L), as usually performed in echocardiography in small rodents[10]. Serial consecutive slices (1mm each) were obtained from the SAX view and volumes were calculated from manually drawn diastolic and systolic areas using the disk summation method[11].

Perfusion fixation and tissue sampling

Six weeks after surgery, animals were anesthetized with an i.v. Avertin® injection and sacrificed by iv injection of 0.5 ml of 2.5N KCl, to arrest the heart in diastole. Retrograde perfusion fixation of the heart via the abdominal aorta was performed using 2% buffered paraformaldehyde solution. The hearts were then excised, quickly rinsed in saline, blotted; ventricles were weighted, and put in 4% buffered formalin for 24 h before processing for inclusion in paraffin (Sakura, Tissue-Tek VIP ).

Morphometric analysis

Infarct size and ventricular dimensions were measured immediately below the coronary ligation on a 5 μm section stained with hematoxylin-eosin, using image analysis software (*Image Tool software,* freely available online). Infarct size was calculated by manually tracing perimeters and expressed as percentage of the left ventricle as follows:

*[(external infarct perimeter+internal infarct perimeter) x 100]/(total external LV perimeter+total internal LV perimeter)*

External and internal perimeters, and LV wall and septum thickness were traced manually. Perimeters were assumed to be the circumference of a circle and used to calculate transverse chamber diameter, LV chamber area and mean ventricular wall thickness.

Capillary density, collagen deposition and myofibroblast number analyses

Staining for capillaries was carried out with Rhodamine-labelled Griffonia simplicifolia Lectin 1 (Vector Laboratories, Peterborough, UK). Nuclei were counterstained with bisbenzimide. Capillaries were counted in the zone bordering the infarction at a magnification of 600x (Olympus IX51), and expressed as capillary number /mm^2^. Collagen deposition was performed by picrosirius red staining of histologic sections of Saline, CTR and VPA cells-injected hearts. Data are expressed as percentage of picrosisius red^+^ area in the whole section. For myofibroblasts determination, paraffin embedded sections were de-waxed, rinsed twice in H_2_O and microwave-treated in 0.01M Tris, 1mM EDTA, pH 9.1 for 30min below boiling conditions. After 30 min at room temperature, slides were washed with H_2_O and twice with PBS. Sections were incubated overnight at 4°C with anti-α-SMA (kindly donated by Dr Christine Chaponnier, Geneva). Subsequently, tissues were incubated with TRITC-Goat-anti-mouse IgG (Sigma) for 1 hour at room temperature. Nuclei were stained with bisbenzimide. Capillary profiles were counted in the peri-infarct area (LV free wall) in at least 10 high-power fields (600x) in two separate 5 μm sections/mouse[12]. Alpha-SMA positive cells (i.e. myofibroblasts) were counted in the infarct area in at least 20 high power-fields (1000x) in two different 5 μm sections/mouse[13]**.**

Engraftment analysis

DNA from Saline, CTR and VPA hearts was extracted using DNEasy blood and tissues kit (Qiagen); 465 ng of DNA/reaction were used. The DNA amplification was performed by RT PCR using a TaqMan Genotyping protocol (Applied Biosystem). Specific primers and VIC and FAM fluorescent TaqMan probes were purchased. These primers were designed on the human genomic sequence (Xq13.1b) containing the SNP C/T (rs6625561 Reference: NCBI SNP). This sequence is highly conserved in humans and does not have homologues within mouse genome. The amplification protocol consisted in a hot start step for 10 minutes at 95° C, followed by 50 cycles including: 1) denaturation for 15^sec^ at 92°C and 2) annealing/amplification for 1^min^ at 60°C. Calibration curve was performed by amplifying under these conditions a 10-folds dilution series starting from 6 ng and ending to 0.6 pg of human DNA extracted from CD34 cells, mixed with a 465 ng of HL1 mouse cardiomyocytes DNA.

**Supplementary References**

1. Yoder MC, Mead LE, Prater D, Krier TR, Mroueh KN, et al. (2007) Redefining endothelial progenitor cells via clonal analysis and hematopoietic stem/progenitor cell principals. Blood 109: 1801-1809.

2. Nanni S, Benvenuti V, Grasselli A, Priolo C, Aiello A, et al. (2009) Endothelial NOS, estrogen receptor beta, and HIFs cooperate in the activation of a prognostic transcriptional pattern in aggressive human prostate cancer. J Clin Invest 119: 1093-1108.

3. Andersen CL, Jensen JL, Orntoft TF (2004) Normalization of real-time quantitative reverse transcription-PCR data: a model-based variance estimation approach to identify genes suited for normalization, applied to bladder and colon cancer data sets. Cancer Res 64: 5245-5250.

4. Saeed AI, Sharov V, White J, Li J, Liang W, et al. (2003) TM4: a free, open-source system for microarray data management and analysis. Biotechniques 34: 374-378.

5. Eisen MB, Spellman PT, Brown PO, Botstein D (1998) Cluster analysis and display of genome-wide expression patterns. Proc Natl Acad Sci U S A 95: 14863-14868.

6. Dennis G, Jr., Sherman BT, Hosack DA, Yang J, Gao W, et al. (2003) DAVID: Database for Annotation, Visualization, and Integrated Discovery. Genome Biol 4: P3.

7. Mestdagh P, Van Vlierberghe P, De Weer A, Muth D, Westermann F, et al. (2009) A novel and universal method for microRNA RT-qPCR data normalization. Genome Biol 10: R64.

8. Wright GW, Simon RM (2003) A random variance model for detection of differential gene expression in small microarray experiments. Bioinformatics 19: 2448-2455.

9. Claycomb WC, Lanson NA, Jr., Stallworth BS, Egeland DB, Delcarpio JB, et al. (1998) HL-1 cells: a cardiac muscle cell line that contracts and retains phenotypic characteristics of the adult cardiomyocyte. Proc Natl Acad Sci U S A 95: 2979-2984.

10. Yang X-P, Liu Y-H, Rhaleb N-E, Kurihara N, Kim HE, et al. (1999) Echocardiographic assessment of cardiac function in conscious and anesthetized mice. Am J Physiol Heart Circ Physiol 277: H1967-1974.

11. Schiller NB, Shah PM, Crawford M, DeMaria A, Devereux R, et al. (1989) Recommendations for quantitation of the left ventricle by two-dimensional echocardiography. American Society of Echocardiography Committee on Standards, Subcommittee on Quantitation of Two-Dimensional Echocardiograms. J Am Soc Echocardiogr 2: 358-367.

12. Salio M, Chimenti S, De Angelis N, Molla F, Maina V, et al. (2008) Cardioprotective function of the long pentraxin PTX3 in acute myocardial infarction. Circulation 117: 1055-1064.

13. Orlandi A, Hao H, Ferlosio A, Clement S, Hirota S, et al. (2009) Alpha actin isoforms expression in human and rat adult cardiac conduction system. Differentiation 77: 360-368.
